# *N*-alkylation briefly constructs tunable multifunctional sensor materials: Multianalyte detection and reversible adsorption

**DOI:** 10.1016/j.isci.2021.103126

**Published:** 2021-09-14

**Authors:** Chu-Ming Pang, Xi-Ying Cao, Ying Xiao, Shi-He Luo, Qi Chen, Yong-Jun Zhou, Zhao-Yang Wang

**Affiliations:** 1 School of Chemistry, South China Normal University; Key Laboratory of Theoretical Chemistry of Environment, Ministry of Education; Guangzhou Key Laboratory of Analytical Chemistry for Biomedicine, Guangzhou 510006, P. R. China; 2 School of Health Medicine, Guangzhou Huashang College, Guangzhou 511300, P. R. China; 3 Key Laboratory of Functional Molecular Engineering of Guangdong Province, School of Chemistry and Chemical Engineering, South China University of Technology, 381 Wushan Road, Guangzhou 510640, P. R. China

**Keywords:** organic chemistry, Photonics, polymers, Optical materials

## Abstract

A series of *N*-alkyl-substituted polybenzimidazoles (**SPBIs**), synthesized by simple condensation and *N*-alkylation, act as functional materials with tunable microstructures and sensing performance. For their controllable morphologies, the formation of nano-/microspheres is observed at the *n*(RBr)/*n*(PBI) feed ratio of 5:1. Products with different degrees of alkylation can recognize metal ions and nitroaromatic compounds (NACs). For example, **SPBI-c**, obtained at the feed ratio of 1:1, can selectively detect Cu^2+^, Fe^3+^, and NACs. By contrast, **SPBI-a**, obtained at the feed ratio of 0.1:1, can exclusively detect Cu^2+^ with high sensitivity. Their sensing mechanisms have been studied by FT-IR spectroscopy, SEM, XPS, and DFT calculations. Interestingly, the **SPBIs** can adsorb Cu^2+^ in solution and show good recyclability. These results demonstrate that polymeric materials with both sensing and adsorption applications can be realized by regulating the alkylation extent of the main chain, thus providing a new approach for the facile synthesis of multifunctional materials.

## Highlights

• Tunable multifunctional sensor materials were synthesized by simple reaction
• Morphology and sensing property can be controlled by the extent of *N*-alkylation
• **SPBIs** can selectively and sensitively detect multiple analytes by dual channels
• **SPBIs** can reversibly and efficiently adsorb Cu^2+^ as indicated with UV-*vis* signal

## Graphical abstract

**Figure:**
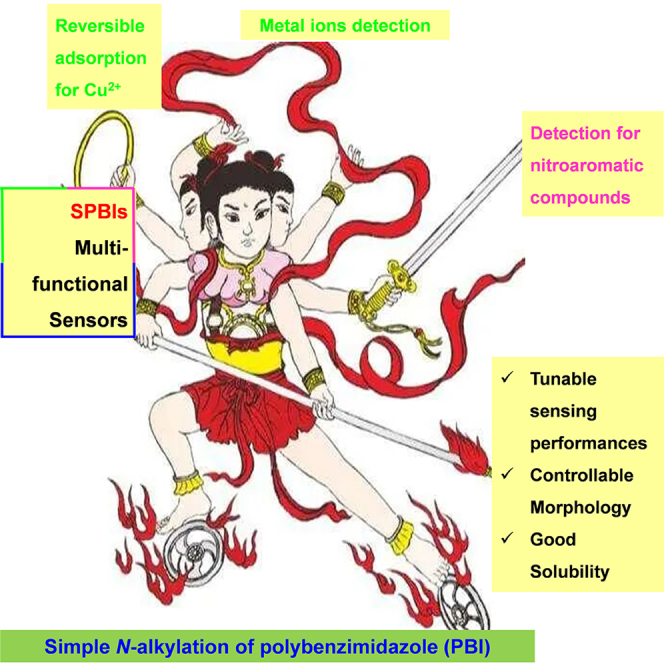

## Introduction

The multianalyte detection concept, which was first proposed by De Silva's group (Magri et al., 2006; Schmittel and Shu, 2012; Chen et al., 2015; Magri, 2021), has become a hotspot in the field of sensors owing to its advantages of high efficiency, rapidity, simultaneous recognition and *in-situ* detection (Xu et al., 2017; Park et al., 2018; Zhang et al., 2018; Chen et al., 2019a; Zhao et al., 2021; Slenders et al., 2021; Huang et al., 2021b; Nakamitsu et al., 2021). Multianalyte sensors were originally designed for multiple metal ions (Magri et al., 2006), but these systems have subsequently been developed for multiple bioactive molecules (Yin et al., 2018; Zhang et al., 2020b; Thanzeel et al., 2020), bacteria (Zheng et al., 2018), etc. In some cases, multifunctional sensors based on fluorescent gels (Zhang et al., 2018; Ozay et al., 2020) or polymers (Ozay et al., 2020; Liu et al., 2020; Huang et al., 2021a) can simultaneously remove analytes, thus reducing the cost of pollutant treatment in practical applications. However, there are still some challenges in the field of multifunctional sensor materials. For example, when recognizing multiple analytes, the sensitivity of sensor may be reduced to some extent and it is slightly lower than that of a traditional sensor (Schmittel and Shu, 2012; Lochman et al., 2019). Moreover, there is an urgent need to develop a simple, universal method for the preparation of multifunctional sensing materials.

**Figure 1:**
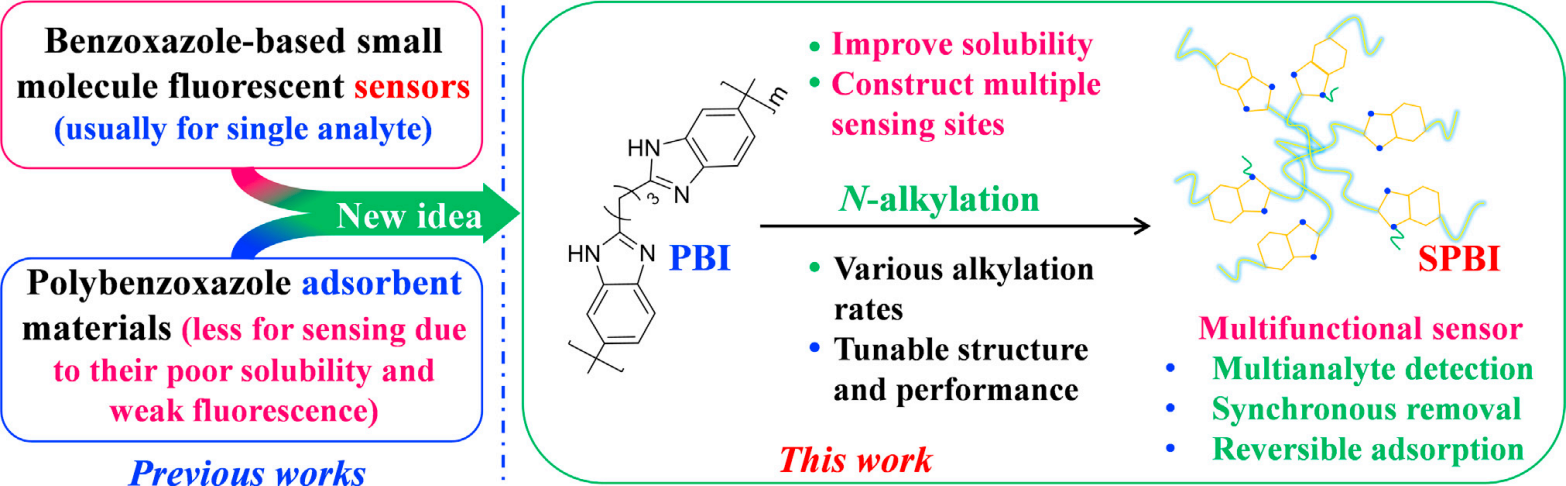
Construction of a multifunctional polymeric sensor based on substituted polybenzimidazole (**SPBI**)

Benzoxazole materials, which show eye-catching fluorescent properties, are not limited to the field of covalent organic frameworks (COFs) (Seo et al., 2019). For example, small benzimidazole fluorescent molecules can sensitively and rapidly detect various analytes through different interactions (Wu et al., 2016, 2018; Ge et al., 2018; Jiang et al., 2019; Chen et al., 2020b). However, the small adsorption capacities of these materials hinder adsorption applications, and thus polymeric or other macromolecular materials are required to achieve simultaneous analyte removal by benzoxazole-based sensors (Rabbani et al., 2017; Lv et al., 2021). Although polybenzimidazole (**PBI**) has been widely applied in a variety of areas (Liang et al., 2019; Geng et al., 2019; Wang et al., 2019; Shan et al., 2019; Cui et al., 2021; Jin et al., 2021), there are only a few reports on sensing (Park and Gong, 2017; Diao et al., 2018; Kaur et al., 2019) owing to the disadvantages of unmodified **PBI**, including poor solubility, weak fluorescence, and difficulties in achieving uniform dispersion in sensing systems. We speculated that the introduction of flexible chains into the backbone of **PBI** through simple *N*-alkylation would weaken π-π stacking between the polymer chains, thus reducing aggregation-caused quenching phenomena and improving the fluorescence properties of **PBI**. Moreover, the introduction of alkyl chains could improve the solubility of **PBI**, which is beneficial for multifunctional sensing (Figure 1).

**Scheme 1:**
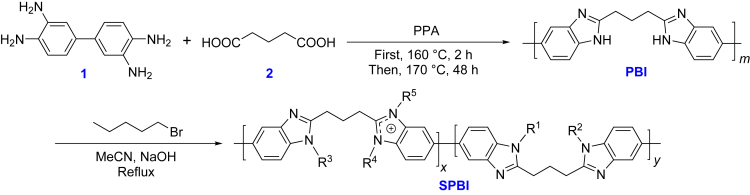
Synthetic route for substituted polybenzimidazole (**SPBI**)

Herein, we report the design and synthesis of a series of substituted polybenzimidazole (**SPBI**) sensing materials *via* a metal-free catalytic route (Scheme 1) as well as the applications of these materials. **PBI** with a linear framework was constructed by dehydration condensation between 3,3′-diaminobenzidine (**1**) and glutaric acid (2), a dicarboxylic fatty acid. Then, a series of **SPBIs** with tunable microstructures and sensing performance was obtained by further modification of **PBI** *via* simple *N*-alkylation at different feed ratios (Table 1).

**Table 1:** Effect of feed ratio on the basic structure of **SPBI**

Sample	Feed ratio (RBr/PBI)	R^1^	R^2^	R^3^	R^4^	R^5^
**SPBI-a**	0.1:1	Pentyl-	H	H	H	–
**SPBI-b**	0.5:1	Pentyl-	H	H	H	–
**SPBI-c**	1:1	Pentyl-	Pentyl-	Pentyl-	H	–
**SPBI-d**	2:1	Pentyl-	Pentyl-	Pentyl-	H	–
**SPBI-e**	3:1	Pentyl-	Pentyl-	Pentyl-	H	–
**SPBI-f**	4:1	Pentyl-	Pentyl-	Pentyl-	Pentyl-	Pentyl-
**SPBI-g**	5:1	Pentyl-	Pentyl-	Pentyl-	Pentyl-	Pentyl-

## Results and discussion

### Characterization and basic properties of SPBIs

**Figure 2:**
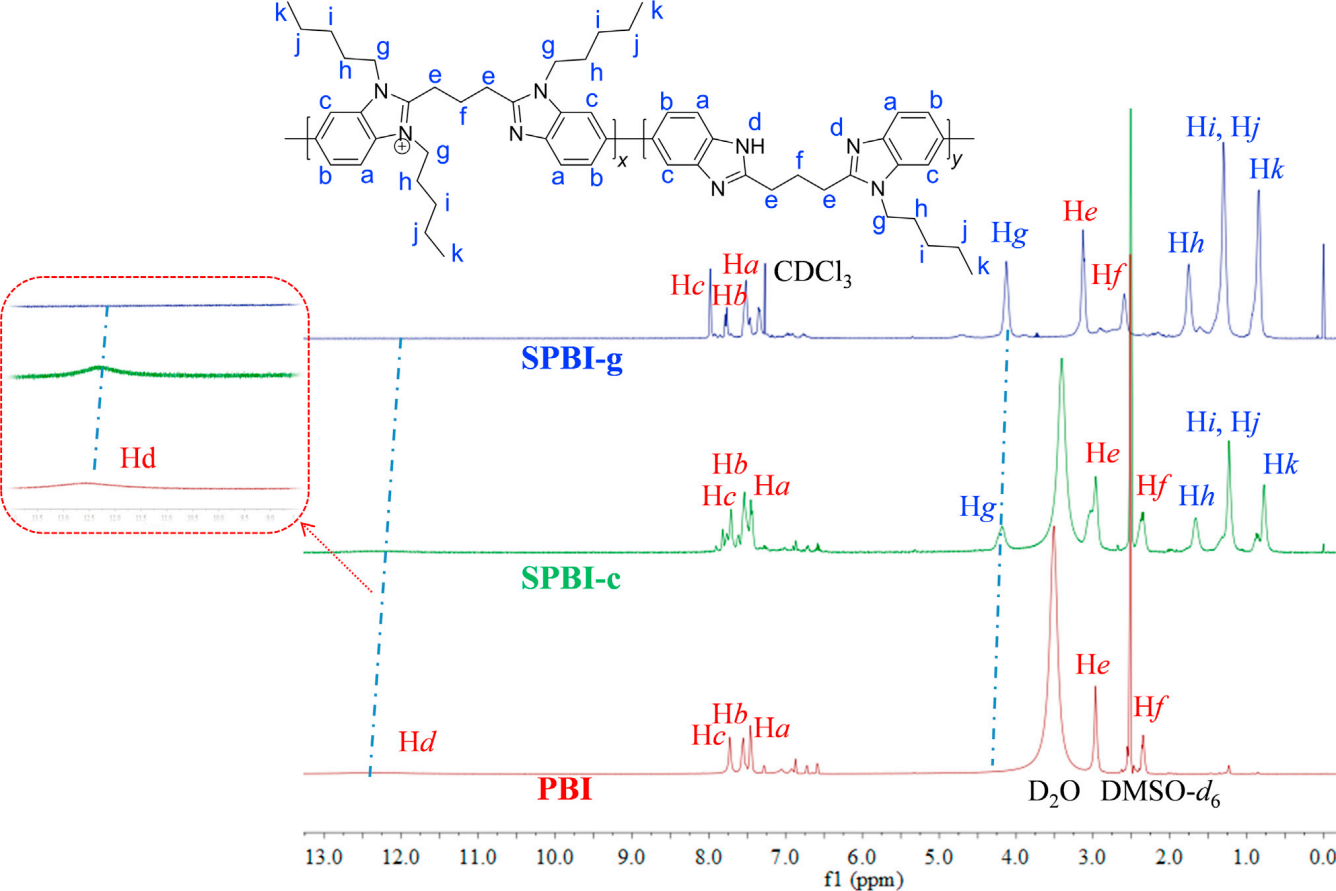
^1^H NMR spectra of **PBI**, **SPBI-c**, and **SPBI-g**

The structures of materials were systematically characterized by ^1^H NMR (Figures S1–S9 and Table S1), FT-IR spectroscopy (Figure S10 and Table S2), PXRD, and XPS. In ^1^H NMR spectrum of **PBI**, the chemical shifts at 7.41–7.48, 7.51–7.61, and 7.65–7.82 ppm were assigned to aromatic hydrogens (H_a_, H_b_, and H_c_) in the repeating unit, and that at 12.35 ppm was assigned to -NH- (H_d_) in the benzimidazole ring. Further, the chemical shifts at 2.31–2.36 and 2.92–2.98 ppm corresponded to the alkyl segment (H_f_ and H_e_) in the repeating unit of **PBI**. The signals for -CH_2_- at the end of polymer chain (1.20–1.50 and 2.54–2.63 ppm; Figure S1) were used to estimate the number-average molecular weight (Mn), and the results are summarized in Table S3. Here, we discuss the results for **SPBI-c** and **SPBI-g** (Table 1) as representative examples [see the Supplemental Information for further characterization data and spectra of **PBI** and **SPBIs** (Figures S1–S8 and Table S1)]. New characteristic signals, including those at 0.69–0.90 ppm (H_k_), 1.10–1.32 ppm (H_i_ and H_j_), 1.57–1.75 ppm (H_h_), and 4.28 ppm (H_g_), were observed in **SPBI** prepared by the *N*-alkylation of **PBI** (Figures 2 and S9). Moreover, the N-H (H_d_) signal was weakened gradually, indicating the successful alkylation of **PBI**.

In the FT-IR spectra, **SPBI** samples exhibited similar characteristic peaks (Figure S10 and Table S2), with a gradual enhancement of the stretching vibrations (at 2957, 2931, and 2855 cm^−1^) of saturated C-H and its bending vibration (at 1414 cm^−1^), and the vibrations of the -CH_2_- groups in alkyl segments (at 724 cm^−1^) as the proportion of C_5_H_11_Br increased. These changes reveal an increase in the alkylation ratio of **SPBI**.

The XPS spectra of **SPBI** products were measured according to a previously reported method (Yu et al., 2019; Li et al., 2021). As shown in Figure S11A, the peaks located at 285.1, 397.6, and 528.1 eV were attributed to C1s, N1s, and O1s, respectively, in the backbone of **SPBI-c**. Moreover, the high-resolution C1s spectrum showed four peaks at 284.6, 285.1, 285.5, and 286.2 eV (Figure S11B), assigned to C-C/C=C, C-N/C-O, C=N, and C=O, respectively, in the terminal group of **SPBI-c**. The N1s spectrum could be divided into two peaks at 399.9 and 401.6 eV (Figure S11C), corresponding to C-N and C=N, respectively (Jiao et al., 2019; Li et al., 2019b). Thus, the XPS data further confirm the construction of a polymer skeleton.

Using reported methods (Geng et al., 2019; Zhang et al., 2019a, 2019b; To et al., 2020; Fan et al., 2021), the crystallinity and thermal stability of each material were investigated. As depicted in Figure S12, the PXRD pattern of **PBI** exhibited a broad peak at 15°–35°, indicating an amorphous state. This broad peak remained after alkylation, demonstrating that the **SPBI** materials are also amorphous. The thermal stabilities of the **SPBI** were investigated under a N_2_ atmosphere. As shown in Figure S13, after being modified by alkyl chains, the **SPBI** began to decompose in the range of 416.6–419.8°C, with the termination of thermal weight loss occurring at 470.0–477.4°C (Table S4), demonstrating the good thermal stability of the **SPBIs**.

**Figure 3:**
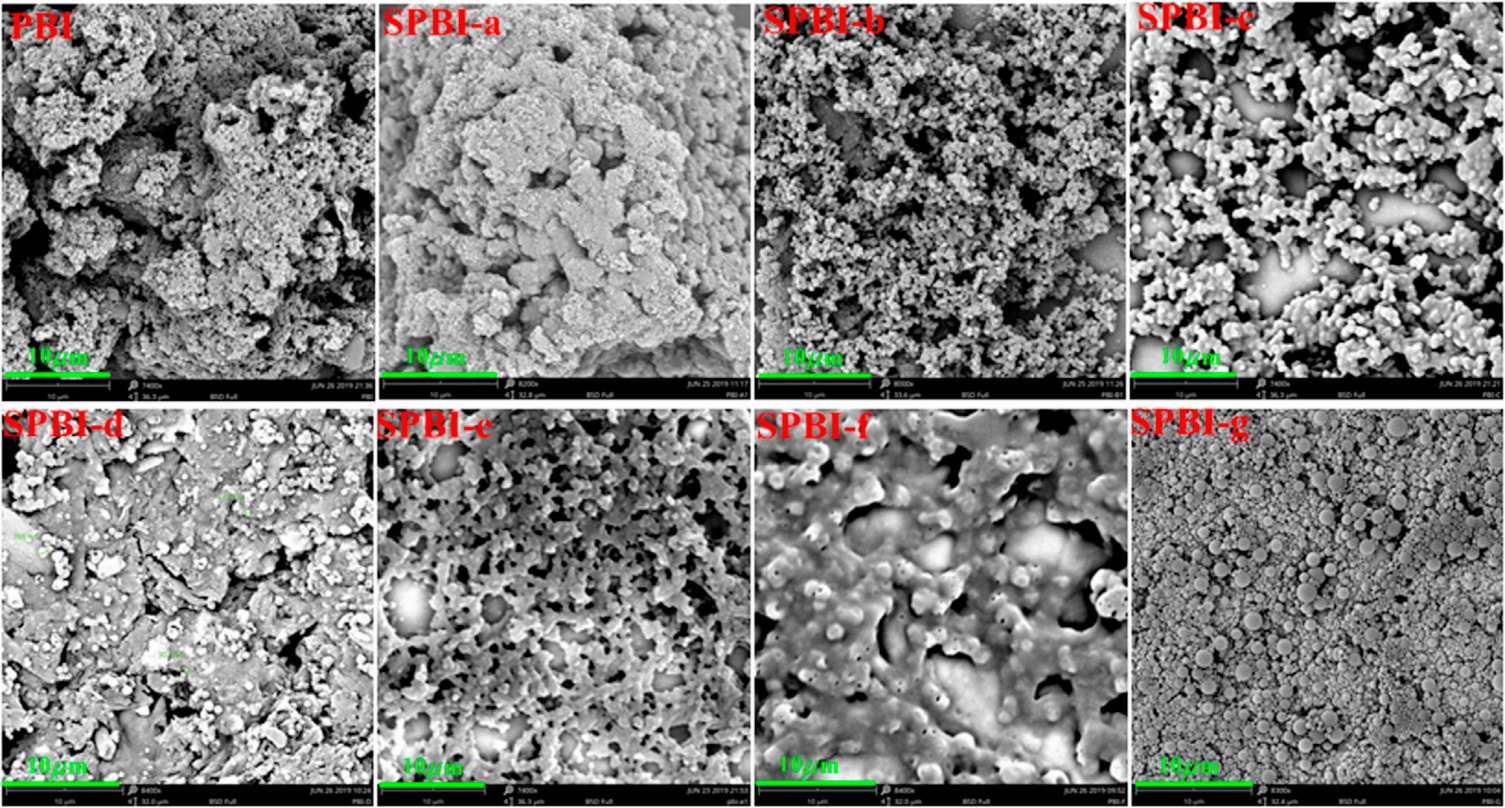
SEM images of **SPBIs** prepared using different feed ratios

Importantly, the morphologies of materials were evaluated by SEM as reported methods (Shan et al., 2019; Zhang et al., 2019a; To et al., 2020; Fan et al., 2021) (Figure 3). The surface of **PBI** was uneven and loose powder with stacked micropores was located between the layers. **SPBI-a**, prepared using an *n*(C_5_H_11_Br)/*n*(**PBI**) feed ratio of 0.1:1, showed a morphology similar to that of **PBI**. In contrast, the morphology of **SPBI-b** (feed ratio of 0.5:1) began to change into small particles, indicating the effective modification with alkyl chains. Furthermore, **SPBI-c** (feed ratio of 1:1) formed a network structure. Some small balls were observed in **SPBI-d** (feed ratio of 2:1) and a regular network was observed for **SPBI-e** (feed ratio of 3:1). Further increasing the feed ratio to 5:1 (**SPBI-g**) resulted in the formation of some regular nanospheres, owing to ionization during the *N*-alkylation of **PBI**. This salt-type product was affected by electrostatic repulsion to form nanoparticles. Thus, not only the extent of **PBI** alkylation but also the morphology of the alkylated products can be adjusted by using the feed ratio of the reactants. In particular, nanoparticles can be formed at a high feed ratio when increasing *n*(C_5_H_11_Br).

Obviously, the *N*-alkylation of **PBI** is random and heterogeneous, but this randomness at different feed ratios automatically regulates the polymer structure due to the existence of the steric hindrance. Therefore, for the preparation process of **SPBI**, it is very simple to wholly regulate the structures and properties of the polymer. And the following researches on the performance and application of **SPBI** further prove this design.

### The photophysical properties of serial SPBIs

The physical and spectral properties of **SPBIs** were investigated. The solubility was improved by the introduction of alkyl, as expected. The **SPBIs** could be dissolved in various common organic solvents, such as dichloromethane (DCM), EtOH, and *N,N-*dimethyl formamide (DMF). The extent of alkylation for the **SPBIs** is summarized in Table S3. As the feed ratio increased, the alkylation rate and yield increased gradually. When the feed ratio was 4:1, ionization began to occur (Table 1). The accompanying slight color change from gray to brown-red (Table S3) implied that the extent of alkylation may have an effect on the spectral properties of **SPBIs**.

The UV-*vis* and fluorescence spectra of **PBI** and **SPBIs** were explored in dimethyl sulfoxide (DMSO) or DMF (with increased alkylation, the solubility of the **SPBIs** in polar DMSO decreases; therefore, DMF was used as the solvent for **SPBI-f** and **SPBI-g**) (Ge et al., 2018; Chen et al., 2020b). As shown in Figure S14, the UV-*vis* absorption spectra of **SPBIs** were similar, with absorption peaks located at approximately 300 nm. The shoulder peak observed at 265 nm was mainly caused by the π-π∗ electronic transition on the conjugated skeleton. As the degree of alkylation increased, the absorbance of **SPBIs** decreased slightly. The fluorescence of **PBI** was so weak under the excitation of 328 nm and there was an obvious enhancement for the fluorescence intensity of **SPBIs** with the emission peaks red-shifted to approximately 447 nm at the same test conditions. The extent of alkylation had little effect on the emission peak position but changed the fluorescence intensity. The Stokes shifts of **SPBI-a** ∼ **SPBI-g** were in the range of 56–87 nm, and the relative fluorescence quantum yields were 34.1%, 31.6%, 32.7%, 43.6%, 28.9%, 50.7%, and 51.2%, respectively. The enhancement of fluorescence might be because of the decrease in π-π interaction between the benzimidazole units in the backbone of **PBI** (Yang et al., 2017; Xie et al., 2017).

According to the SEM analysis mentioned above, the morphologies of **SPBI-c** (network structure) and **SPBI-g** (nano-spheres) were relatively regular. Therefore, they were selected as representative compounds for evaluating the UV-*vis* and fluorescence spectra in THF, MeCN, EtOH, DMF, and DMSO. As shown in Figures S15 and S16, the UV-*vis* absorption spectra of **SPBI-c** and **SPBI-g** in various solvents showed slight differences. In particular, the absorbance of **SPBI-c** was largest in DMSO, and an absorption tail appeared in EtOH. For **SPBI-g**, an absorption tail was observed in MeCN. A comparison of the fluorescence spectra in different solvents showed that the fluorescence intensities of **SPBI-c** and **SPBI-g** were relatively strong in DMF and DMSO.

The N atoms in the **SPBIs** can easily coordinate metal ions, and the products with lower degrees of alkylation (e.g., **SPBI-a**) have strong π-π interactions and short distances between the polymer chains. Therefore, only metal ions with a suitable size can enter the framework, promoting interactions between analytes and the polymeric sensor. As the extent of alkylation increases, the π-π interactions decrease, accompanying the increase of the distance between the polymer chains. These may affect the interaction of metal ions with **SPBIs** and will be beneficial for interactions between the **SPBIs** and nitro-aromatic compounds (NACs) with the larger molecular sizes (Dou et al., 2014; Zeng et al., 2016). To explore the influence of alkylation degree, **SPBI-a**, **SPBI-b**, **SPBI-c**, and **SPBI-g** were used as representative compounds to investigate the sensing performance.

### Sensing performance of SPBIs toward metal ions

Metal ions (e.g., Cu^2+^) play vital roles in human physiological processes. However, trace metal ions can be amplified through the food chain owing to the non-degradability of metal ions and their accumulation in organisms (Chen et al., 2021). As excess amounts of some metal ions in the body may cause various diseases (Jiang et al., 2018; Pang et al., 2019; Khairy and Duerkop, 2019), it is necessary to develop new sensor materials for the convenient detection of metal ions. The selectivity of **SPBI-a** (6.0% alkylation) for 16 metal ions was investigated in the DMSO/H_2_O system.

**Figure 4:**
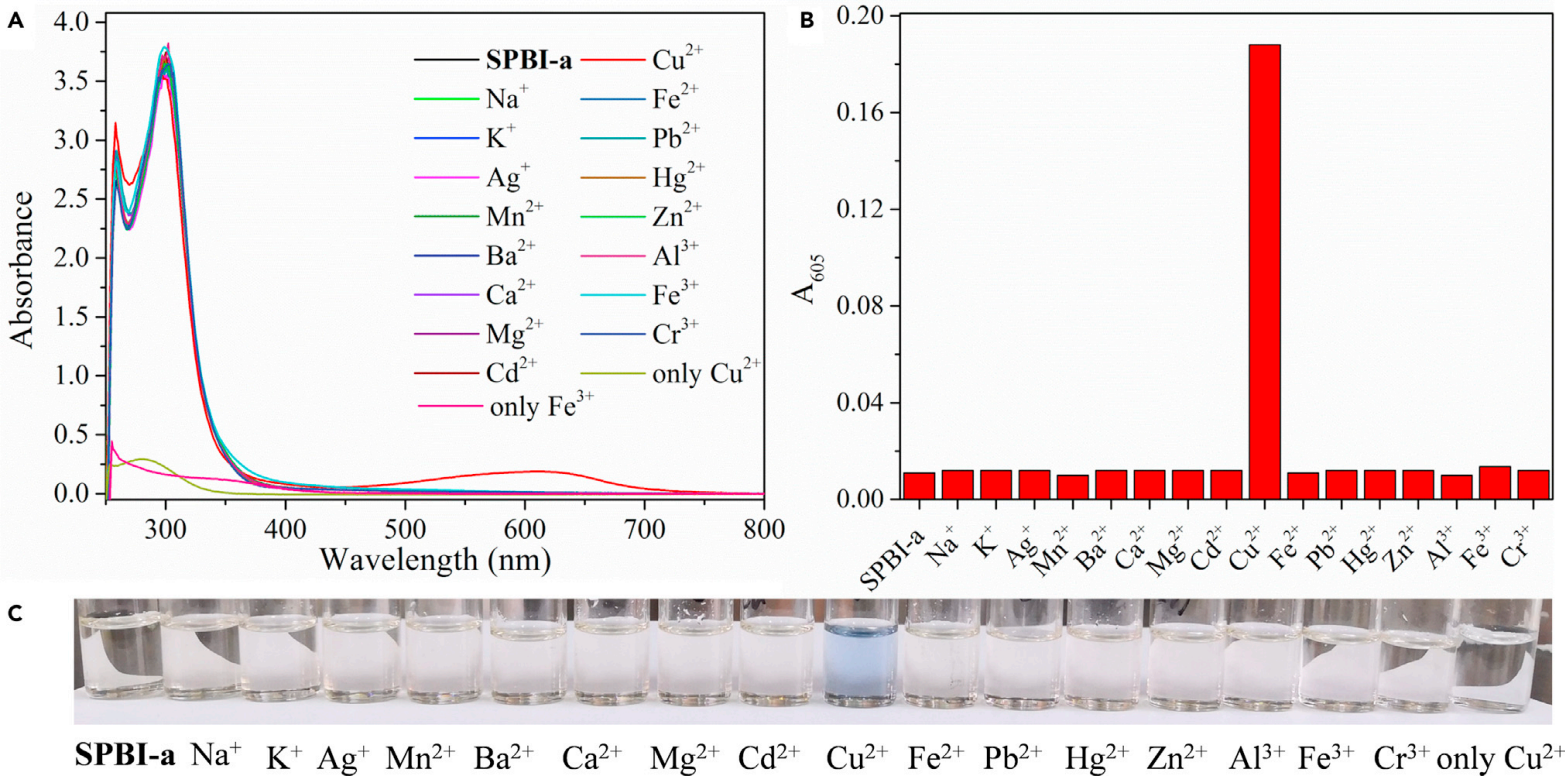
UV-*vis* absorption spectra, absorbance changes at 605 nm, and color changes of **SPBI-a** (DMSO/H_2_O = 99:1, v/v) after the addition of various metal ions (50 *μ*M) (A) UV-vis absorption spectra changes. (B) Absorbance changes at 605 nm. (C) Color changes.

As shown in Figure 4, the UV-*vis* absorption peak of **SPBI-a** was located at 303 nm with a shoulder peak at 265 nm. When Cu^2+^ was added, the absorbance of the sensing system at 303 nm decreased slightly, and the absorbance at 275 and 350–800 nm increased significantly, causing the solution color change from colorless to indigo (Figure 4C). This result indicated that **SPBI-a** shows a colorimetric response to Cu^2+^. To rule out that this change was due to the color of Cu^2+^ itself, the UV-*vis* spectrum of an aqueous solution containing only the same amount of Cu^2+^ was collected. The aqueous solution containing only Cu^2+^ had an absorbance of less than 0.5 and appeared to be colorless. As shown in Figure 4B, the absorbance of the sensing system at 605 nm increased obviously with the addition of Cu^2+^, and the absorption of the system at 605 nm was unchanged with the addition of Fe^3+^. The other metal ions also had few effects on the systems. Therefore, **SPBI-a** can be used as a Cu^2+^ colorimetric sensor.

Interference experiments demonstrated that the UV-*vis* spectra of this system were not affected significantly by other ions, with the exception of Fe^3+^, which had only a small effect (Figure S17), indicating the good anti-interference ability of **SPBI-a** in response to Cu^2+^. Furthermore, the interaction between **SPBI-a** and Cu^2+^ was explored. As shown in Figure S18, with an increase in the Cu^2+^ concentration, the absorbance of **SPBI-a** at 303 nm decreased accompanied by a slight of redshift, and the absorbance at 250–280 and 350–800 nm increased gradually, resulting in a change in the solution color from colorless to blue. These observations indicate that there is a strong interaction between **SPBI-a** and Cu^2+^ (Aysha et al., 2019). Importantly, the system gradually reached a saturated state when the concentration of Cu^2+^ is approximately 80 *μ*M. Using a reported method (Kumar and Chae, 2019; Chen et al., 2019a, 2019b; Zhao et al., 2019), the LOD was calculated as 8.76 × 10^−7^ M (Figure S19), which is equivalent to the sensitivity of some Cu^2+^ sensors reported recently (Table S6) (Han et al., 2019; Wu et al., 2020b; Wei et al., 2020).

Using reported methods (Imase et al., 2003; Kim et al., 2013), the sensing performance of **SPBI** materials obtained at other feed ratios were also investigated. For example, using UV-*vis* absorption spectroscopy, **SPBI-b** was found to selectively recognize two metal ions (Cu^2+^ and Fe^3+^) with LODs of 4.50 × 10^−7^ and 1.49 × 10^−8^ M, respectively (Figures S20–S26). With increased alkylation, the distance between the polymer chains might increases and the interactions of the **SPBI** with analytes might be affected, resulting in changes in sensitivity (Tables S6 and S7). **SPBI-c** (28.5% alkylation) and **SPBI-g** (65.0% alkylation) could also discern Cu^2+^ and Fe^3+^ but with different sensitivities (Figures S27–S39 and further discussions can be seen in the **SI**). The signals of **SPBI-c** were more sensitive to Cu^2+^ than those of **SPBI-g**, and its sensitivity was also better than those of some reported metal ion sensors (Table S6) (Kim et al., 2013; Zhang et al., 2020a; Du et al., 2020). For Fe^3+^, the sensitivity of **SPBI-c** was also higher than that of **SPBI-g** as well as those of some reported Fe^3+^ sensors (Table S7) (Li et al., 2019a, 2019b; Fan et al., 2020; Cui et al., 2020; Zheng et al., 2020; Shia et al., 2020; Jia et al., 2020; Sun et al., 2020). Therefore, the sensing performance of the **SPBI** is controllable, and the most sensitive material must have a suitable size for metal ions to coordinate with N atoms in **SPBI**. Herein, **SPBI-c** obtained at *n*(C_5_H_11_Br)/*n*(**PBI**) = 1:1 was found to exhibit the best performance.

### Application of SPBIs in cyclic adsorption for Cu^2+^

At present, there are many types of materials used for adsorption. Especially, recycling these adsorption materials can save the cost of materials to a large extent and is conducive to environmental protection (Liu et al., 2020, 2021; Huang et al., 2021a; Wang et al., 2020b; Chen et al., 2020a), especially for multifunctional material (Chen et al., 2021). Using Cu^2+^ as an example, the cyclic adsorption application of **SPBIs** was investigated (Figure S40). Treatment with HCl (pH = 2), EDTA solution, and deionized water promoted Cu^2+^ desorption from the **SPBIs**, allowing these materials to be used for the reversible adsorption of Cu^2+^ under the indication by UV-*vis* absorbance spectra. The adsorption rates and adsorption capacities are summarized in Table S5.

Owing to the small distance between the polymer chains of **SPBI-a** with a low alkylation degree, Cu^2+^ could not easily enter into the polymer framework to coordinate (Imase et al., 2003), but the surface adsorption still occurred. The initial Cu^2+^ adsorption rate was only 75.07% with poor recyclability. For **SPBI-g** with a high alkylation degree, the large distance between polymer chains was also not conducive to interactions with metal ions (Imase et al., 2003; Kim et al., 2013), resulting in an adsorption performance similar to that of **SPBI-a**.

**Figure 5:**
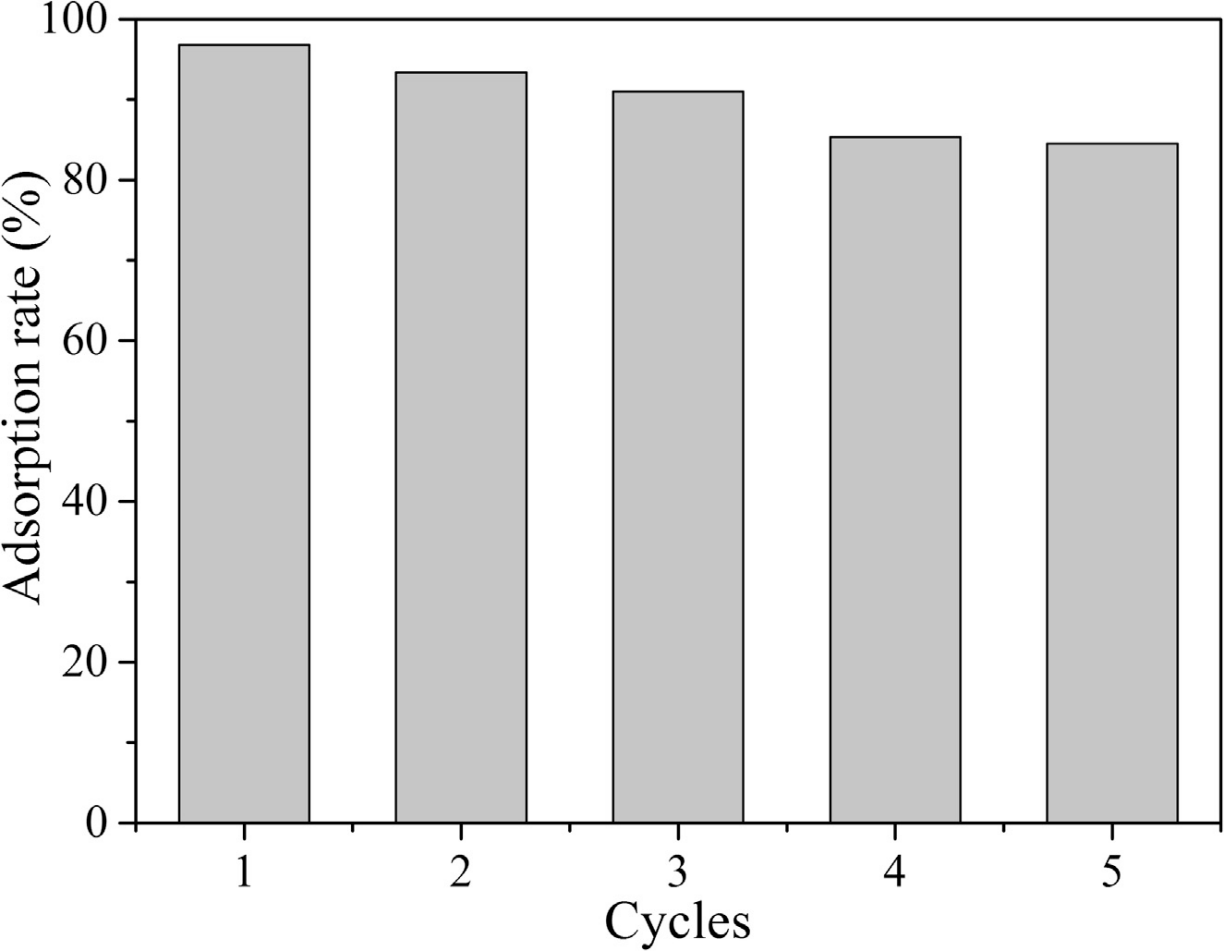
Reversible adsorption for Cu^2+^ by **SPBI-c**

Interestingly, for **SPBI-c**, the moderate alkylation degree provided a suitable distance between polymer chains that allowed Cu^2+^ to enter into the framework and coordinate with N atoms, resulting in good adsorption performance with an initial Cu^2+^ adsorption rate of 96.81%. Moreover, this material maintained an adsorption rate of more than 80% in five cycles (Figure 5). Importantly, the recycling performance of **SPBI** was similar to those of some reported reusable adsorption materials (Table S9) (Liu et al., 2020; Huang et al., 2021a; Wang et al., 2020b; Chen et al., 2020a).

### Sensing performance of SPBIs toward NACs

NACs are important raw materials for some blasting equipment and in the leather industry. Owing to the significant impact of these compounds on public safety, human life, and property, the trace detection of NACs is also a hot topic in the sensor field (Chen et al., 2020c; Wang et al., 2019; Zhuang et al., 2020b; Kasthuri et al., 2019). When the extent of alkylation increases, the π-π interactions in **SPBIs** decrease, accompanying by an increase in distance between polymer chains (Imase et al., 2003; Kim et al., 2013) These may be beneficial to interactions between C=N in **SPBI** and NACs with the larger molecular sizes. Thus, **SPBI** might be applicable to the recognition of electron-deficient NACs, and the selectivity of **SPBI-c** (28.5% alkylation) toward different NACs (the structures are shown in Figure S41) was tested.

**Figure 6:**
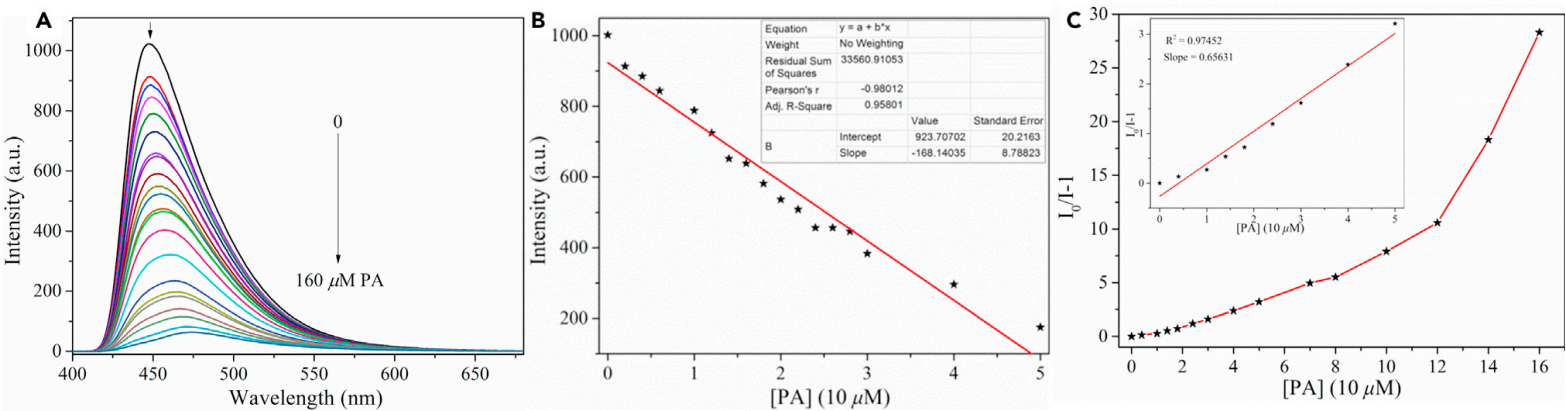
Fluorescence spectra, linear plot, and Stern-Volmer plot for **SPBI-c** (DMSO/H_2_O = 99:1, V/V) upon the addition of different concentrations of PA (A) Fluorescence spectra for **SPBI-c** with the addition of PA (λ_ex_ = 331 nm). (B) Linear plot for **SPBI-c** with the addition of PA. (C) Stern-Volmer plot for **SPBI-c** with the addition of PA

**SPBI-c** was found to selectively detect PA, DNP, and NP by fluorescence quenching (Figure S42). Using a reported method (Jiao et al., 2019; Zhang et al., 2019a), the fluorescence quenching efficiencies of three typical NACs (PA, DNP, and NP) for **SPBI-c** were studied (Figure 6 and S43). With 160 *μ*M PA, a maximum quenching efficiency of 96.8% was obtained, whereas higher concentrations of DNP and NP were required to achieve similar quenching efficiencies, demonstrating the high sensitivity of **SPBI-c** to PA. Further-more, using a reported approach (Gao et al., 2019; Nandi et al., 2020), the LODs of **SPBI-c** for PA, DNP, and NP were calculated to be 1.81 × 10^−7^, 2.29 × 10^−7^ and 2.62 × 10^−7^ M, respectively, which are equivalent to the LODs of some reported NAC sensors (Gao et al., 2019; Nandi et al., 2020; Ghorai et al., 2019; Dutta et al., 2020).

The sensitivity of fluorescence-quenching sensors to analytes can also be judged from the Stern-Volmer (S-V) constant, K_*sv*_, which can be calculated as follows: I_0_/I = 1 + K_*sv*_[Q]. As shown in Figures 6C and S43C, for PA, DNP, or NP, the S-V plot curved upward at higher concentrations, indicating that this process involves a combination of dynamic and static quenching (Goswami et al., 2019; Rajak et al., 2019). In the low concentration range, the S-V plot is linear, and the K_*sv*_ values for **SPBI-c** toward PA, DNP, and NP were 6.5631 × 10^4^, 4.5122 × 10^4^, and 3.8651 × 10^4^ M^−1^, respectively. These K_*sv*_ values are higher than those of the most reported fluorescent polymer sensors for NACs detection (Ghorai et al., 2019; Dutta et al., 2020).

Moreover, **SPBI-g** was also found to sensitively detect PA, DNP, and NP (Figure S44), with LODs of 1.68 × 10^−7^, 1.93 × 10^−7^, and 2.15 × 10^−7^ M, respectively (Figures S45 and S46), and K_*sv*_ values of 3.75 × 10^4^, 3.85 × 10^4^, and 2.68 × 10^4^ M^−1^, respectively (Figure S47). The sensitivities of **SPBI-g** and **SPBI-c** to PA, DNP, and NP were on the same order of magnitude, but **SPBI-g** showed higher sensitivity for the detection of PA (Chen et al., 2020c; Wu et al., 2020a) (for a detailed comparison, see Table S8).

### Sensing mechanism of SPBIs for multianalyte

**Figure 7:**
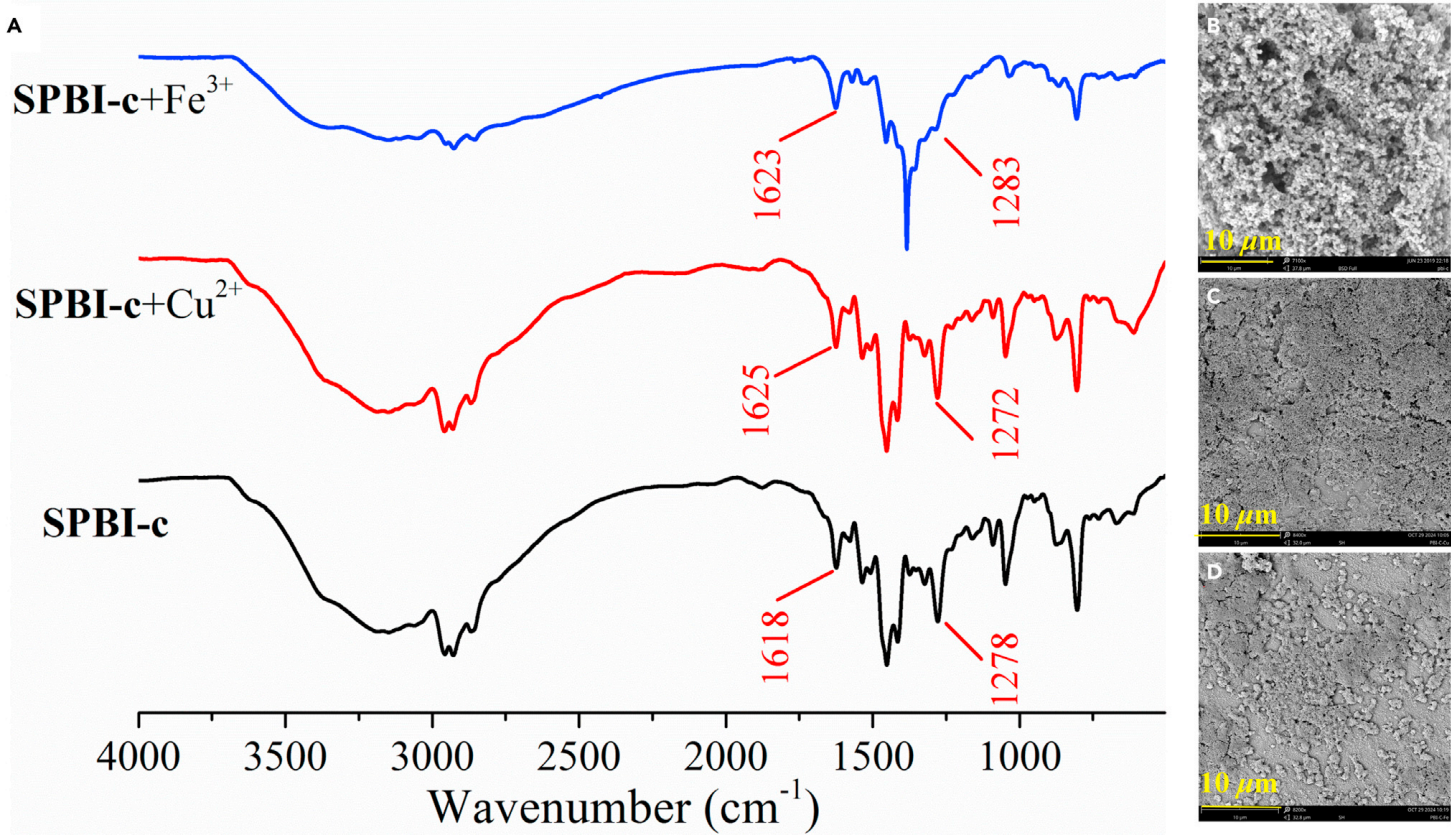
FT-IR spectra, SEM images of **SPBI-c** before and after the addition of Cu^2+^ or Fe^3+^ (A) FT-IR spectra of **SPBI-c** before and after the addition of Cu^2+^ or Fe^3+^. (B) SEM images of **SPBI-c**. (C) SEM images of **SPBI-c** after the addition of Cu^2+^. (D) SEM images of **SPBI-c** after the addition of Fe^3+^.

According to the reported method (Li et al., 2019b; Zhang et al., 2019b; Chen et al., 2021; Chen et al., 2020c; Wang, et al., 2020), the effects of analytes on the FT-IR spectra and morphology of **SPBIs** were determined. Using **SPBI-c** as a representative compound, the changes in FT-IR spectra and SEM images before and after the addition of Cu^2+^ or Fe^3+^ were observed. As depicted in Figure 7, the stretching vibration of C-N and C=N in **SPBI-c** are located at 1278 and 1618 cm^−1^, respectively. After the addition of Cu^2+^, these stretching vibrations moved to 1272 and 1625 cm^−1^, respectively, indicating an interaction between the N atoms in **SPBI-c** and Cu^2+^ (Wu et al., 2016; Li et al., 2019b). Similarly, these stretching vibrations also moved after the addition of Fe^3+^ to **SPBI-c**. In addition, the morphology of **SPBI-c** changed from a network structure to a dense honeycomb structure, confirming an interaction between **SPBI-c** and metal ions (Zeng et al., 2016). The interactions of **SPBI-a** (Figure S48), **SPBI-b** (Figure S49), **SPBI-c** (Figure S50), and **SPBI-g** (Figures S51 and S52) with different analytes (metal ions and NACs) were also examined by FT-IR spectroscopy and SEM.

In particular, for the detection of NACs, the quenching process at low concentrations can be determined from the fluorescence lifetime changes (Figure S53) (Kasthuri et al., 2019; Zhuang et al., 2020b). The fluorescence lifetime decay curves of **SPBI-c** and **SPBI-g** were less affected by NACs. Therefore, the quenching of NACs in the low concentration range can be considered a static quenching process (Jiang et al., 2019; Chen et al., 2020c).

**Figure 8:**
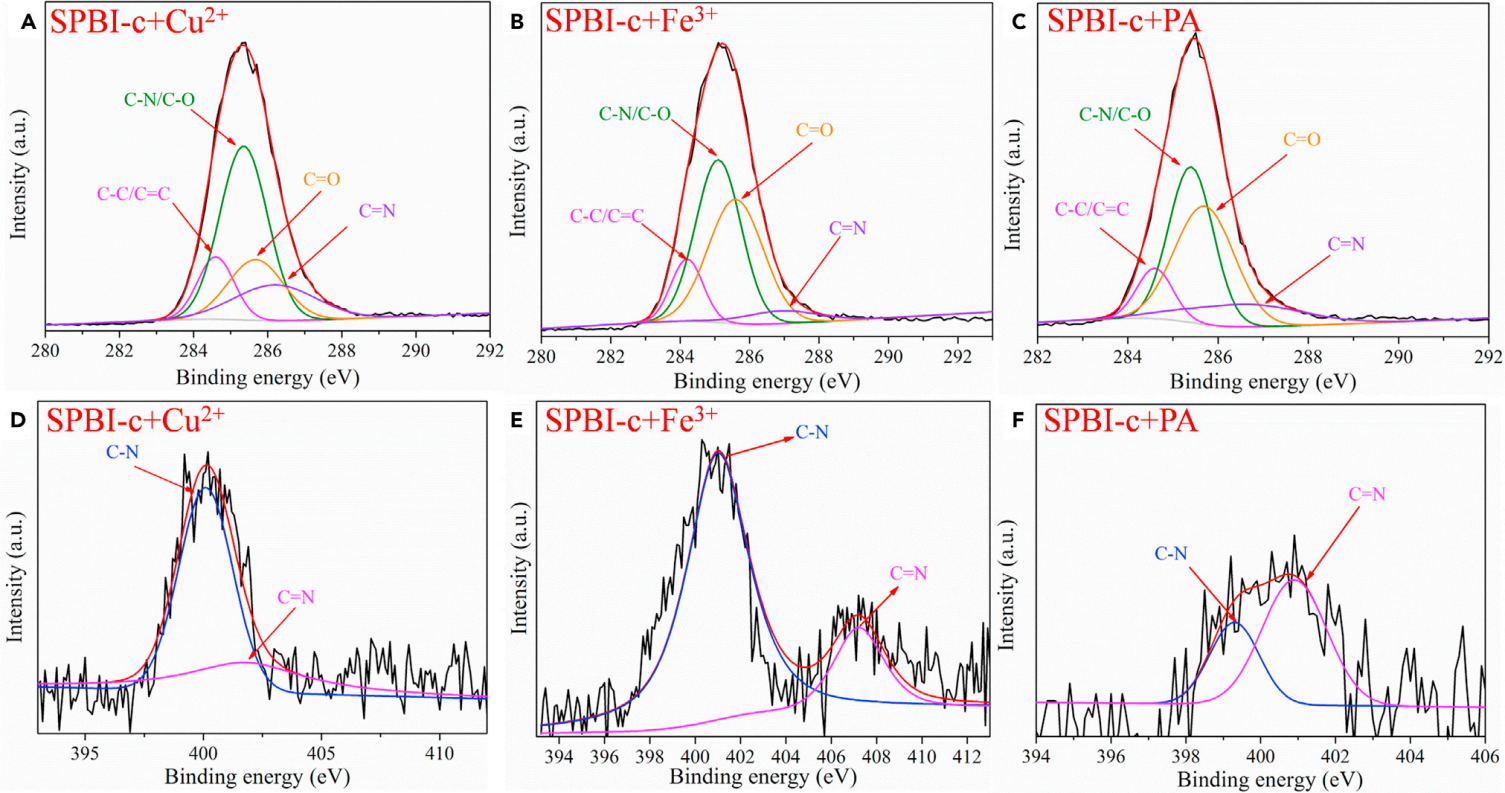
C1s and N1s XPS spectra of **SPBI-c** after the addition of Cu^2+^, Fe^3+^, and PA (A) C1s XPS spectra of **SPBI-c** after the addition of Cu^2+^. (B) C1s XPS spectra of **SPBI-c** after the addition of Fe^3+^. (C) C1s XPS spectra of **SPBI-c** after the addition of PA. (D) N1s XPS spectra of **SPBI-c** after the addition of Cu^2+^. (E) N1s XPS spectra of **SPBI-c** after the addition of Fe^3+^. (F) N1s XPS spectra of **SPBI-c** after the addition of PA.

In addition, the interactions between polymeric sensors and analytes can be measured by XPS analysis (Li et al., 2019b; Zhao et al., 2019; Wang et al., 2020c). Using **SPBI-c** as an example, the C1s and N1s binding energies in the system after the addition of Cu^2+^, Fe^3+^, and PA were determined. As shown in Figures 8A and 8D, upon the addition of Cu^2+^, the C1s binding energy of C=N in **SPBI-c** moved from 286.1 to 286.6 eV, and the N1s binding energy moved from 401.6 to 402.3 eV. These changes demonstrate the presence of a coordination effect between Cu^2+^ and C=N in **SPBI-c** (Li et al., 2019b). Similar interactions were confirmed between **SPBI-c** and Fe^3+^ (Figures 8B and 8E) or PA (Figures 8C and 8F).

The structures of the representative compound **SPBI-c**, its Cu^2+^ complex and PA were optimized by DFT-B3LYP/6-31G in Gaussian 09 software. As shown in Figure S54, the electron cloud of the frontier orbital in polymer is mainly distributed on the benzimidazole unit and the energy of the highest occupied molecular orbital (HOMO) is −0.39 eV. Although the energy of the HOMO orbital for PA is −4.01 eV, which is lower than the HOMO energy level of the polymer. This facilitates the photoinduced electron transfer (PET) effect produced between the polymers and PA, leading to the fluorescence quenching (Zhuang et al., 2020a, 2020b). Meanwhile, the structure of the metal complex also has been optimized by taking the Cu^2+^ complex as a representative. It can be found that, when coordinated with Cu^2+^, the energy gap of HOMO-LUMO orbital for the polymer (**SPBI-c**) is reduced (Figure S54), forming a more stable metal complex (Park and Gong, 2017; Zeng et al., 2016; Pang et al., 2019). This further proves the coordination between the metal ion and the polymer.

**Figure 9:**
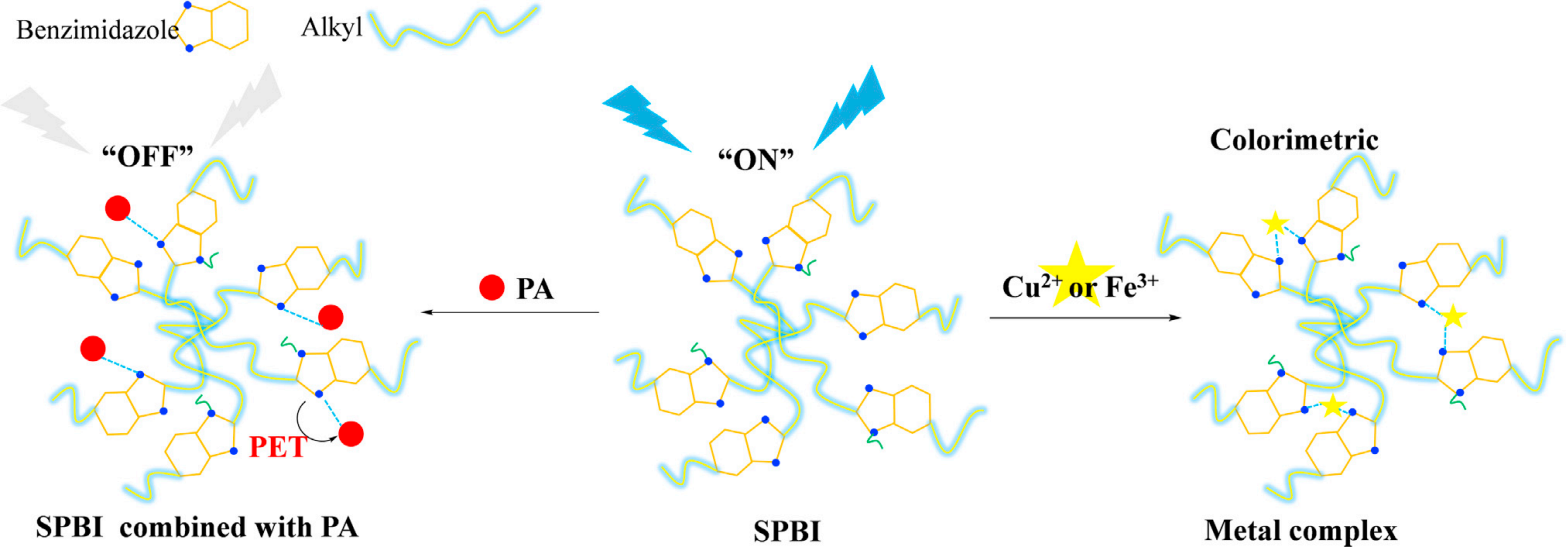
Interaction models for **SPBI** with metal ions (Cu^2+^, Fe^3+^) or PA

Based on the above analysis, interaction models for the **SPBIs** with metal ions (Cu^2+^ or Fe^3+^) or PA were proposed (Figure 9). The metal ions can coordinate with the C=N bonds in the polymer chains to form a stable metal complex, leading to a change in UV-*vis* absorption signal of the system and realizing the colorimetric detection of Cu^2+^ or Fe^3+^ by **SPBIs**. For PA, the -OH group can form hydrogen bonds with multiple C=N groups in the polymer backbone (Jiang et al., 2019; Chen et al., 2020c; Kasthuri et al., 2019), inducing fluorescence quenching of the sensing system by photoinduced electron transfer (PET). Thus, the **SPBIs** can also be used for the recognition of PA.

### Conclusions

In summary, **PBI**, a linear polymer with poor solubility, was prepared by a simple condensation reaction. Subsequently, its solubility was improved *via N*-alkylation, and a series of **SPBI** were developed for the first time as colorimetric and ratiometric sensing materials. **SPBI** with different extents of alkylation were obtained by controlling the feed ratio of C_5_H_11_Br and **PBI**. Furthermore, the morphology of **SPBI** could be adjusted, and a tendency toward nano-/microsphere formation was observed with increased alkylation. It was found that the interactions between polymer molecules decreased and the distance between polymer chains increased at a high degree of alkylation, causing variations in the sensing performance of **SPBI** toward metal ions and NACs. Moreover, the **SPBI** were capable of adsorbing Cu^2+^ in solution, and good cyclability was achieved with the aid of an acid and a strong coordinating agent. **SPBI-c**, obtained at the feed ratio of 1:1, exhibited the best adsorption performance. This work provides a new idea for the facile synthesis of multifunctional materials.

### Limitations of the study

Reversible experiments need to judge the adsorption effect and desorption degree of the material, mainly through the color changes of the material before and after adsorption, and controlling the time of adsorption and desorption for comparison. Among them, the color discrimination has certain subjective factors, and it may affect the adsorption effect and the degree of desorption in the research process. Therefore, the time was controlled to make the adsorption and desorption effect have certain comparability. Of course, since the adsorption capacity of conjugate organic polymers is influenced by the factors like temperature, pH, adsorption time, and so on, the adsorption experiment may be further optimized.

## STAR★Methods

### Key resources table

**Table:** 

REAGENT or RESOURCE	SOURCE	IDENTIFIER
**Chemicals, peptides, and recombinant proteins**		

*3,3′*-diaminobenzidine	Energy chemical technology	CAS: 91-95-2
Glutaric acid	Energy chemical technology	CAS: 110-94-1
Bromopentane (*n*-C_5_H_11_Br)	Energy chemical technology	CAS: 110-53-2
Polyphosphoric acid (PPA)	Macklin biochemical technology	CAS: 8017-16-1
Potassium nitrate (KNO_3_)	Guangzhou Chemical reagent factory	CAS: 7757-79-1
Sodium nitrate (NaNO_3_)	Guangzhou Chemical reagent factory	CAS: 7631-99-4
Silver nitrate (AgNO_3_)	Guangzhou Chemical reagent factory	CAS: 7761-88-8
Barium nitrate [Ba(NO_3_)_2_]	Guangzhou Chemical reagent factory	CAS: 10,022-31-8
Calcium nitrate [Ca(NO_3_)_2_]	Guangzhou Chemical reagent factory	CAS: 10,124-37-5
Manganese nitrate tetrahydrate [Mn(NO_3_)_2_·4H_2_O]	Guangzhou Chemical reagent factory	CAS: 20,694-39-7
Copper Sulfate (CuSO_4_)	Guangzhou Chemical reagent factory	CAS: 7758-98-7
Ferrous chloride [FeCl_2_]	Guangzhou Chemical reagent factory	CAS: 7758-94-3
Lead nitrate [Pb(NO_3_)_2_]	Guangzhou Chemical reagent factory	CAS: 10,099-74-8
Mercury nitrate [Hg(NO_3_)_2_]	Guangzhou Chemical reagent factory	CAS: 10,045-94-0
Zinc nitrate [Zn(NO_3_)_2_]	Guangzhou Chemical reagent factory	CAS: 7779-88-6
Aluminum trichloride (AlCl_3_)	Guangzhou Chemical reagent factory	CAS: 7446-70-0
Ferric chloride (FeCl_3_)	Guangzhou Chemical reagent factory	CAS: 7705-08-0
Chromium chloride hexahydrate (CrCl_3_·6H_2_O)	Guangzhou Chemical reagent factory	CAS: 10,060-12-5

**Deposited data**		

Raw and analyzed data	This paper	NA

**Software and algorithms**		

Gaussian 09	Gaussian Inc.	Yimo Information Technology Co., Ltd

### Resource availability

#### Lead contact

Further requests for resources regarding this study will be fulfilled by the corresponding author, Zhao-Yang Wang (wangzy@scnu.edu.cn).

#### Materials availability

This work did not produce any new unique reagents.

### Experimental model and subject details

This work did not need any unique experimental model.

### Method details

#### Materials

3,3′-Diaminobenzidine, glutaric acid, and bromopentane (*n*-C_5_H_11_Br) were purchased from Energy chemical technology (Shanghai) Co. Ltd. Polyphosphoric acid (PPA) was purchased from Macklin biochemical technology Co. Ltd. All soluble metal ion salts and sodium hydroxide (NaOH) are purchased from Guangzhou Chemical reagent factory. All the nitroaromatic compounds purchased from Guangzhou Chemical reagent factory. Tetrahydrofuran (THF), acetonitrile (MeCN), anhydrous ethanol (EtOH), dimethyl formamide (DMF), dichloro-methane (DCM), and dimethyl sulfoxide (DMSO) were purchased from Tianjin Damao chemical reagent factory. DMSO-*d*_*6*_ and CDCl_3_ were purchased from Energy chemical technology (Shanghai) Co. Ltd. All these reagents were used without further purification.

#### Apparatus

The scanning electron microscopy (SEM) image was obtained on Phenom Pro X Desktop Scanning Electron Microscope and Energy Spectrum Integrated Machine. The thermogravimetric analysis was tested in TG-209 F3 thermogravimetric analyzer. X-ray photoelectron spectroscopic (XPS) analysis was performed by an Axis Ultra-DLD X-ray photoelectron spectrometer. The fluorescent spectra were obtained with a Hitachi F-4600 spectrophotometer at room temperature using the xenon lamp as light source; the slit width was 5 nm for both excitation and emission and voltage was 400 V. The UV-*vis* absorption spectra were carried out with an SHIMADZU UV-2700 UV spectrometer. The pH values were recorded by a PHS-25C meter. Fluorescent lifetime was measured by FLS 920 Fluorescence Spectrometer. The metal ion adsorption detection was carried out by AAS-990 atomic absorption spectrophotometer.

#### Synthesis of intermediate PBI

As the reported method (Wu et al., 2016, 2018; Ge et al., 2018; Jiang et al., 2019; Chen et al., 2020b), 3.0 mmol *3,3′*-diaminobenzidine, 3.0 mmol glutaric acid and 20 mL polyphosphoric acid (PPA) were added into a 50 mL round-bottom flask. The mixture was stirred at 120°C for 2 h, and then heated to 170°C for 48 h. Once the reaction was stopped, the pH of the mixture was adjusted to alkaline with NaOH solution till cooling to room temperature. A crude solid product was obtained by vacuum filtration. Subsequently, the crude product was washed several times with water, ethyl acetate, ethanol and other solvents to remove the unreacted raw materials and some by-products with the lower molecular weight. Finally, the product was dried in a vacuum drying oven at 50°C for 24 h.

#### Synthesis of serial SPBIs

According to reported methods (Wu et al., 2016, 2018; Ge et al., 2018; Jiang et al., 2019; Chen et al., 2020b), 1 mmol **PBI**, different molar *n*-C_5_H_11_Br, 10 mL MeCN and moderate NaOH were added into the reaction flask. After refluxing for 24 h, the solvent was removed by vacuum distillation. The product was washed with water several times to remove the NaOH. Then, the alkylated product was rinsed alternately with dichloromethane and ethanol, collecting the organic phase and removing the solvent to obtain the purified product. Finally, the anticipated product was dried in a vacuum drying oven at 40°C for 24 h.

#### General procedure for optical spectral measurements

The **SPBI** samples were dissolved in DMSO or DMF to acquire a stock solution. Then, the test solution (DMSO/H_2_O or DMF/H_2_O as *V*/*V* = 99/1) of **SPBI** was prepared for the experiments of both UV-*vis* absorption spectra and fluorescence spectra at room temperature. Among them, 1 mg sample was dissolved into 15 mL mixed solvent.

#### Limit of detection

As the reported (Seo et al., 2014; Giri et al., 2018), the limit of detection (LOD) was measured by the equation: LOD = 3*δ*/*K*. Therein, *δ* is the standard deviation of the blank measurements (n = 12), and the *K* is the slope of the calibration curve.

#### SEM analysis

According to the reported method (Zhang et al., 2019a; To et al., 2020), the morphology for **SPBI** and the morphology changes of **SPBI** combined with metal ions or nitroaromatic compounds (NACs) were determined by field emission scanning electron microscope.

#### XPS analysis

As reported method (Wang et al., 2019; Li et al., 2019b), the combining energy of N1s, O1s and C1s in the product and complex were measured by Axis Ultra-DLD X-Ray photoelectron spectrometer

#### Cyclic adsorption and desorption

According to the literature (Wang et al., 2019; Ozay et al., 2020), to realize the recycle adsorption of **SPBI**, the coordinated solid was treated with HCl (pH = 2) and EDTA solution, and the mixture was stirred for 30 min. Once the solid color was changed from blue to colourless, the regenerating **SPBI** solid was obtained by filtering. After neutralizing the acid remained on the surface of **SPBI** with NaOH solution (pH = 10), and rinsing with the deionized water to a neutral environment, the coordinated N atoms in **SPBI** were restored to their original state. The concentrations of samples were tested by AAS.

### Quantification and statistical analysis

The limit of detection was obtained by the formula “LOD = 3σ/k” and σ was the relative standard deviation. Differences were considered significant at p < 0.05. The statistical analyses were performed with Origin software.

### Additional resources

There are no additional resources needed to be declared in this manuscript, additional requests for this can be made by contacting the lead contact.

## Data Availability

All data are published in this manuscript and supplement; additional requests for data can be made by contacting the lead contact, Zhao-Yang Wang (wangzy@scnu.edu.cn).

## Supporting Citations

The following references appear in the Supplemental information: Abbasi et al., 2019; Abuhatab et al., 2020; Ansari et al., 2020; Bora et al., 2019; Chiou et al., 2020; Coldur et al., 2020; Darmayanti et al., 2019; Fang et al., 2020; Feng et al., 2020; Ge et al., 2020; Ghosh et al., 2019; He et al., 2021; Hosseinjani-Pirdehi et al., 2020; Jiang et al., 2020; Jigyasa et al., 2020; Joseph et al., 2020; Kadian and Manik, 2020; Ke et al., 2020; Krishnan and Suneesh, 2019; Landge et al., 2020; Li et al., 2020a; Li et al., 2020b; Ma et al., 2020a; Ma et al., 2020b; Mal et al., 2020; Mathivanan et al., 2020; Miao, 2019; Nan et al., 2020; Podasca et al., 2019; Qian et al., 2019; Qu et al., 2020; Rajalakshmi and Palanisami, 2020; Ran et al., 2020; Roja et al., 2020; Sharma et al., 2019; Tian et al., 2019; Xiao et al., 2020; Xiong et al., 2019; Xu et al., 2019; Zhu et al., 2020.

## Footnotes

Supplemental information can be found online at https://doi.org/10.1016/j.isci.2021.103126.

## References

[bib1] Abbasi F., Akbarinejad A., Alizadeh N. (2019). CdS QDs/N-methylpolypyrrole hybrids as fluorescent probe for ultrasensitive and selective detection of picric acid. Spectrochim. Acta A.

[bib2] Abuhatab S., El-Qanni A., Al-Qalaq H., Hmoudah M., Al-Zerei W. (2020). Effective adsorptive removal of Zn^2+^, Cu^2+^, and Cr^3+^ heavy metals from aqueous solutions using silica-based embedded with NiO and MgO nanoparticles. J. Environ. Manage..

[bib3] Ansari M., Hassan A., Alam A., Jana A., Das N. (2020). Triptycene based fluorescent polymers with azo motif pendants: eflect of alkyl chain on fluorescence, morphology and picric acid sensing. React. Funct. Polym..

[bib4] Aysha T.S., El-Sedik M.S., Mohamed M.B.I., Gaballah S.T., Kamel M.M. (2019). Dual functional colorimetric and turn-off fluorescence probe based on pyrrolinone ester hydrazone dye derivative for Cu^2+^ monitoring and pH change. Dyes Pigm..

[bib5] Bora A., Mohan K., Dolui S.K. (2019). Carbon dots as cosensitizers in dye-sensitized solar cells and fluorescence chemosensors for 2,4,6-trinitrophenol detection. Ind. Eng. Chem. Res..

[bib6] Chen H.F., Zhou Y., Wang J.Y., Lu J., Zhou Y.B. (2020). Polydopamine modified cyclodextrin polymer as efficient adsorbent for removing cationic dyes and Cu^2+^. J. Hazard. Mater..

[bib7] Chen K., Shu Q.H., Schmittel M. (2015). Design strategies for lab-on-a-molecule probes and orthogonal sensing. Chem. Soc. Rev..

[bib8] Chen S.-H., Jiang K., Xiao Y., Cao X.-Y., Arulkumar M., Wang Z.-Y. (2020). Recent endeavors on design, synthesis, fluorescence mechanisms and applications of benzazole- based molecular probes toward miscellaneous species. Dyes Pigm..

[bib9] Chen S.-H., Pang C.-M., Chen X.-Y., Yan Z.-H., Huang S.,-M., Li X.-D., Zhong Y.-T., Wang Z.-Y. (2019). Research progress in design, synthesis and application of multifunctional fluorescent probes. Chin. J. Org. Chem..

[bib10] Chen X.F., Sun C.M., Liu Y., Yu L., Zhang K., Asiri A.M., Marwani H.M., Tan H., Ai Y.J., Wang X.K., Wang S.H. (2020). All-inorganic perovskite quantum dots CsPbX_3_ (Br/I) for highly sensitive and selective detection of explosive picric acid. Chem. Eng. J..

[bib11] Chen X., Huang Z., Luo S.-Y., Zong M.-H., Lou W.-Y. (2021). Multi-functional magnetic hydrogels based on Millettia speciosa Champ residue cellulose and Chitosan: highly efficient and reusable adsorbent for Congo red and Cu^2+^ removal. Chem. Eng. J..

[bib12] Chen X.-B., Qi C.-X., Li H., Ding J.-Y., Yan S., Lei H., Xu L., Liu B. (2019). Highly sensitive and selective Fe^3+^ detection by a water-stable Tb^3+^-doped nickel coordination polymer-based turn-off fluorescence sensor. J. Solid State Chem..

[bib13] Chiou Y.-R., Yan H.B., Wan C.-F., Huang C.Y., Wu A.-T. (2020). A Schiff-based fluorescence sensor for the detection of Cu^2+^ and its application in living cells. J. Photochem. Photobiol. A.

[bib14] Coldur M., Oguzlar S., Ongun M.Z., Oter O., Yıldırım S. (2020). Usage of thiocyanate-based ionic liquid as new optical sensor reagent: absorption and emission based selective determination of Fe (III) ions. Spectrochim. Acta A.

[bib15] Cui X., Si Z.J., Li Y.H., Duan Q. (2020). Synthesis of telechelic PNIPAM ended with 9,10-dihydroacridine group as a recyclable and specific Fe^3+^ detection fluorescent sensor. Dyes Pigm..

[bib16] Cui Y.H., Wang S., Wang D., Liu G., Liu F.X., Liang D., Wang X.D., Yong Z.P., Wang Z. (2021). HT-PEMs based on carbazole grafted polybenzimidazole with high proton conductivity and excellent tolerance of phosphoric acid. J. Membr. Sci..

[bib17] Darmayanti L., Kadja G.T.M., Notodarmojo S., Damanhuri E., Mukti R.R. (2019). Structural alteration within fly ash-based geopolymers governing the adsorption of Cu^2+^ from aqueous environment: Effect of alkali activation. J. Hazard. Mater..

[bib18] Diao H.P., Guo L.X., Liu W., Feng L.H. (2018). A novel polymer probe for Zn(II) detection with ratiometric fluorescence signal. Spectrochim. Acta A.

[bib19] Dou Z.S., Yu J.C., Cui Y.J., Yang Y., Wang Z.Y., Yang D.R., Qian G.D. (2014). Luminescent metal-organic framework films as highly sensitive and fast-response oxygen sensors. J. Am. Chem. Soc..

[bib20] Du T., Wang J., Zhang T.S., Zhang L., Yang C.Y., Yue T.L., Sun J., Li T., Zhou M.G., Wang J.L. (2020). An integrating platform of ratiometric fluorescent adsorbent for unconventional real-time removing and monitoring of copper ions. ACS Appl. Mater. Inter..

[bib21] Dutta B., Hazra A., Dey A., Sinha C., Ray P.P., Banerjee P., Mir M.H. (2020). Construction of a succinate-bridged Cd(II)-based two-dimensional coordination polymer for efficient optoelectronic device fabrication and explosive sensing application. Cryst. Growth Des..

[bib22] Fan C.Y., Wu H., Guan J.Y., You X.D., Yang C., Wang X.Y., Cao L., Shi B.B., Peng Q., Kong Y. (2021). Scalable fabrication of crystalline COF membranes from amorphous polymeric membranes. Angew. Chem. Int. Ed..

[bib23] Fan J., Zhang S.F., Xu Y.S., Wei N., Wan B., Qian L.W., Liu Y. (2020). A polyethylenimine/salicylaldehyde modified cellulose Schiff base for selective and sensitive Fe^3+^ detection. Carbohydr. Polym..

[bib25] Fang X.J., Zhu S.D., Ma J.Z., Wang F.Y., Xu H.H., Xia M.Z. (2020). The facile synthesis of zoledronate functionalized hydroxyapatite amorphous hybrid nanobiomaterial and its excellent removal performance on Pb^2+^ and Cu^2+^. J. Hazard. Mater..

[bib26] Feng B.X., Xu Z., Qi C.G., Guo X.M., Gai L.G. (2020). Fluorescence quenching of photo- luminescent organic polymer nanoflms by ferric ions. Microchem. J..

[bib27] Gao J.M., Chen X.X., Chen S.Q., Meng H., Wang Y., Li C.S., Feng L. (2019). The BODIPY-based chemo-sensor for fluorometric/colorimetric dual channel detection of RDX and PA. Anal. Chem..

[bib28] Ge F.-Y., Sun G.-H., Meng L., Ren S.-S., Zheng H.-G. (2020). Four new luminescent metal- organic frame-works as multi- functional sensors for detecting Fe^3+^, Cr_2_O_7_^2-^ and nitromethane. Cryst. Growth Des..

[bib29] Ge J.Y., Fan L., Zhang K., Ou T., Li Y.H., Zhang C.H., Dong C., Shuang S.M., Wong M.S. (2018). A two-photon ratiometric fluorescent probe for effective monitoring of lysosomal pH in live cells and cancer tissues. Sens. Actuators B.

[bib30] Geng K., Li Y., Xing Y., Wang L.H., Li N.W. (2019). A novel polybenzimidazole membrane containing bulky naphthalene group for vanadium flow battery. J. Membr. Sci..

[bib31] Ghorai P., Dey A., Hazra A., Dutta B., Brandao P., Ray P.P., Banerjee P., Saha A. (2019). Cd(II) based coordination polymer series: fascinating structures, efficient semi-conductors, and promising nitro aromatic sensing. Cryst. Growth Des..

[bib32] Ghosh S., Manna R., Dey S. (2019). Epoxy-based polymer incorporating 1-naphthylamine and sebacic acid moieties: a selective fluorescent sensor for ferric ions. J. Mol. Struct..

[bib134] Giri D., Bankura A., Patra S.K. (2018). Poly(benzodithieno-imidazole- alt-carbazole) based π-conjugated copolymers: highly selective and sensitive turn-off fluorescent probes for Hg2+. Polymer.

[bib33] Goswami R., Seal N., Dash S.R., Tyagi A., Neog S. (2019). Devising chemically robust and cationic Ni(II)-MOF with nitrogen-rich micropores for moisture-tolerant CO_2_ capture: highly regenerative and ultrafast colorimetric sensor for TNP and multiple oxo-anions in water with theoretical revelation. ACS Appl. Mater. Inter..

[bib34] Han T., Yuan Y., Kang H., Zhang Y., Dong L.J. (2019). Ultrafast, sensitive and visual sensing of copper ions by a dual-fluorescent film based on quantum dots. J. Mater. Chem. C..

[bib35] He Y., Gou S.H., Zhou L.H., Tang L., Liu T., Liu L., Duan M. (2021). Amidoxime- functionalized polyacrylamide-modified chitosan containing imidazoline groups for effective removal of Cu^2+^ and Ni^2+^. Carbohydr. Polym..

[bib36] Hosseinjani-Pirdehi H., Mahmoodi N.O.A., Taheri A., Asalemi K.A.A., Esmaeili R. (2020). Selective immediate detection of Cu^2+^ by a pH-sensitive rhodaminebased fluorescence probe in breast cancer cell-line. Spectrochim. Acta A.

[bib37] Huang L.L., Yu K.H., Zhou W.T., Teng Q.Y., Wang Z.Y., Dai Z.H. (2021). Quantitative principal component analysis of multiple metal ions with lanthanide coordination polymer networks. Sens. Actuators B.

[bib38] Huang Y., Liu L.X., Yang X., Zhang X.Y., Yan B., Wu L., Feng P.J., Lou X.D., Xia F., Song Y.L., Li F.Y. (2021). A diverse micromorphology of photonic crystal chips for multianalyte sensing. Small.

[bib39] Imase T., Ohira A., Okoshi K., Sano N., Kawauchi S., Watanabe J., Kunitake M. (2003). AFM study of two-dimensional epitaxial arrays of poly(*γ*-L-glutamates) with long *n*-alkyl side chains on graphite. Macromolecules.

[bib40] Jia T., Fu M., Zhang M.Y., Qiu J.W., Zhu H., Gao Y. (2020). A novel cholesterol conjugated fluorescence probe for Cu^2+^ detection and bioimaging in living cells. Spectrochim. Acta A.

[bib41] Jiang K., Chen S.-H., Luo S.-H., Pang C.-M., Wu X.-Y., Wang Z.-Y. (2019). Concise design and synthesis of water-soluble fluorescence sensor for sequential detection of Zn(II) and picric acid via cascade mechanism. Dyes Pigm..

[bib42] Jiang K., Wu Y.-C., Wu H.-Q., Li S.-L., Luo S.-H., Wang Z.-Y. (2018). A highly selective, pH-tolerable and fast-response fluorescent probe for Fe^3+^ based on star-shape benzothiazole derivative. J. Photochem. Photobiol. A.

[bib43] Jiang S.J., Qiu J.B., Chen S.B., Guo H.Y., Yang F.F. (2020). Double-detecting fluorescent sensor for ATP based on Cu^2+^ and Zn^2+^ response of hydrazono-bis-tetraphenylethylene. Spectrochim. Acta A.

[bib44] Jiao Y., Gao Y.F., Meng Y.T., Lu W.J., Liu Y., Han H., Shuang S.M., Li L., Dong C. (2019). One-step synthesis of label-free ratiometric fluorescence carbon dots for the detection of silver ions and glutathione and cellular imaging applications. ACS Appl. Mater. Inter..

[bib45] Jigyasa, Kumar D., Arora P., Singh H., Rajput J.K. (2020). Polyhydroquinoline nanoaggregates: a dual fluorescent probe for detection of 2,4,6-trinitrophenol and chromium(VI). Spectrochim. Acta A.

[bib46] Jin Y., Gao B., Bian C., Meng X.X., Meng B., Wong S.I., Yang N.T., Sunarso J., Tan X.Y., Liu S.M. (2021). Elevated-temperature H_2_ separation using a dense electron and proton mixed conducting polybenzimidazole-based membrane with 2D sulfonated grapheme. Green. Chem..

[bib47] Joseph R., Asok A., Joseph K. (2020). Quinoline appended pillar[5]arene (QPA) as Fe^3+^ sensor and complex of Fe^3+^ (FeQPA) as a selective sensor for F^-^, arginine and lysine in the aqueous medium. Spectrochim. Acta A.

[bib48] Kadian S., Manik G. (2020). Green synthesis of fluorescent carbon dots using chloroplast dispersions as precursors and application for Fe^3+^ ion sensing. Luminescence.

[bib49] Kasthuri S., Kumar S., Raviteja S., Ramakrishna B., Maji S., Veeraiahd N., Venkatramaiah N. (2019). Influence of alkyl chains on fluoranthene ensembles towards fluorescence based detection of 2,4,6-trinitrophenol. Appl. Surf. Sci..

[bib50] Kaur H., Singh N., Kaur N., Jang D.O. (2019). Nano-aggregate-Fe^3+^ complex based on benzimidazole-modified calix[4] arene for amplified fluorescence detection of ADP in aqueous media. Sens. Actuators B.

[bib51] Ke H.S., Wei W., Yang Y.S., Wu H.P., Zhang Y.-Q., Xie G., Chen S.P. (2020). A trinuclear zinc coordination cluster exhibiting fluorescence, colorimetric sensitivity, and recycling of silver ion and detection of cupric ion. Inorg. Chem..

[bib52] Khairy G.M., Duerkop A. (2019). Dipsticks and sensor microtiterplate for determination of copper (II) in drinking water using reflectometric RGB readout of digital images, fluorescence or eye-vision. Sens. Actuators B.

[bib53] Kim K.H., Yu H., Kang H., Kang D.J., Cho C.H., Cho H.H., Oh J.H., Kim B.J. (2013). Influence of intermolecular interactions of electron donating small molecules on their molecular packing and performance in organic electronic devices. J. Mater. Chem. A.

[bib54] Krishnan S., Suneesh C.V. (2019). Fluorene-triazine conjugated porous organic polymer framework for superamplifed sensing of nitroaromatic explosives. J. Photochem. Photobiol. A.

[bib55] Kumar A., Chae P.S. (2019). Fluorescence tunable thiophene- bis(benzimidazole)-based probes for a cascade trace detection of Hg^2+^ and lysine: a molecular switch mimic. Sens. Actuators, B.

[bib56] Landge S.M., Lazare D.Y., Freeman C., Bunn J., Cruz J.I., Winder D., Padgett C., Aiken K.S., Ghosh D. (2020). Rationally designed phenanthrene derivatized triazole as a dual chemo- sensor for fluoride and copper recognition. Spectrochim. Acta A.

[bib57] Li A.-L., Wang Z.-L., Wang W.-Y., Liu Q.-S., Sun Y., Wang S.-F., Gu W. (2021). A novel dehydroabietic acid-based fluorescent probe for detection of Fe^3+^ and Hg^2+^ ions and its application in live-cell imaging. Microchem. J..

[bib58] Li B., Zhou J., Bai F.Y., Xing Y.H. (2020). Lanthanide-organic framework based on a 4,4-(9,9-dimethyl-9H-fluorene-2,7-diyl) dibenzoic acid: synthesis, structure and fluorescent sensing for a variety of cations and anions simultaneously. Dyes Pigm..

[bib59] Li X.C., Han Y.J., Sun S.S., Shan D.D., Ma X.M., He G.J., Mergu N., Park J.-S., Kim C.-H., Son Y.-A. (2020). A diaminomaleonitrile-appended BODIPY chemosensor for the selective detection of Cu^2+^ via oxidative cyclization and imaging in SiHa cells and zebrafish. Spectrochim. Acta A.

[bib60] Li X.S., An J.D., Zhang H.M., Liu J.J., Li Y., Du G.X., Wu X.X., Fei L., Lacoste J.D., Cai Z. (2019). Cluster-based Ca^II^, Mg^II^ and Cd^II^ coordination polymers based on aminofunctionalized triphenyl tetra-carboxylate: bifunctional photo -luminescent sensing for Fe^3+^ and antibiotics. Dyes Pigm..

[bib61] Li Y.K., He Y.L., Guo F.Y., Zhang S.P., Liu Y.Y., Lustig W.P., Bi S.M., Williams L.J., Hu J., Li J. (2019). NanoPOP: solution-processable fluorescent porous organic polymer for highly sensitive, selective, and fast naked eye detection of mercury. ACS Appl. Mater. Inter..

[bib62] Liang N.Q., Fang J.H., Guo X.X. (2019). A facile approach for preparation of porous polybenzimidazole membranes as a promising separator for lithium ion batteries. J. Mater. Chem. A.

[bib63] Liu H.Q., Wang Y., Mo W.Q., Tang H.L., Cheng Z.Y., Chen Y., Zhang S.T., Ma H.W., Li B., Li X.B. (2020). Dendrimer-based, high-luminescence conjugated microporous polymer films for highly sensitive and selective volatile organic compound sensor arrays. Adv. Funct. Mater..

[bib64] Liu W., An Z., Qin L., Wang M., Liu X., Yang Y. (2021). Construction of a novel ion imprinted film to remove low concentration Cu^2+^ from aqueous solution. Chem. Eng. J..

[bib65] Lochman L., Machacek M., Miletin M., Uhlířová Š., Lang K., Kirakci K., Zimcik P., Novakova V. (2019). Red-emitting fluorescence sensors for metal cations: the role of counter-anions and sensing of SCN^-^ in biological materials. ACS Sensors.

[bib66] Lv B., Yin H., Shao Z.G., Luan Z.J., Huang Z.Y., Sun S.S., Teng Y., Miu C.H., Gao Q. (2021). Novel polybenzimidazole/graphitic carbon nitride nanosheets composite membrane for the application of acid-alkaline amphoteric water electrolysis. J. Energy Chem..

[bib67] Ma J.Z., Xia M.Z., Zhu S.D., Wang F.Y. (2020). A new alendronate doped HAP nanomaterial for Pb^2+^, Cu^2+^ and Cd^2+^ effect absorption. J. Hazard. Mater..

[bib68] Ma L., Han X., Xia L., Qu F.L., Kong R.-M. (2020). A label-free G-quadruplex-based fluorescence assay for sensitive detection of alkaline phosphatase with the assistance of Cu^2+^. Spectrochim. Acta A.

[bib69] Magri D.C. (2021). Logical sensing with fluorescent molecular logic gates based on photoinduced electron transfer. Coord. Chem. Rev..

[bib70] Magri D.C., Brown G.J., McClean G.D., de Silva A. (2006). Communicating chemical congregation: a molecular AND logic gate with three chemical inputs as a “lab-on-a-molecule” prototype. J. Am. Chem. Soc..

[bib71] Mal K., Naskar B., Chaudhuri T., Prodhan C., Goswami S., Chaudhuri K., Mukhopadhyay C. (2020). Synthesis of quinoline functionalized fluorescent chemosensor for Cu(II), DFT studies and its application in imaging in living HEK 293 cells. J. Photochem. Photobiol. A.

[bib72] Mathivanan M., Tharmalingam B., Mani K.S., Thiagarajan V., Murugesapandia B. (2020). Simple C3-symmetric triaminoguanidine- triphenylamine conjugate as an efficient colorimetric sensor for Cu(II) and fluorescent sensor for Fe(III) ions. Spectrochim. Acta A.

[bib73] Miao C.L. (2019). Three water-stable luminescent Zn(II) coordination polymers for highly sensitive and selective sensing of acetylacetone and Fe^3+^ ions. J. Mol. Struct..

[bib74] Nakamitsu M., Oyama K., Imai H., Fujii S., Oaki Y. (2021). Ultrahigh-sensitive compression-stress sensor using integrated stimuli-responsive materials. Adv. Mater..

[bib75] Nan Z.Z., Hao C.C., Zhang X.G., Liu H.Y., Sun R.G. (2020). Carbon quantum dots (CQDs) modified ZnO/CdS nanoparticles based fluorescence sensor for highly selective and sensitive detection of Fe(III). Spectrochim. Acta A.

[bib76] Nandi S.K., Chowdhury S.R., Podder D., Ghorai P.K., Haldar D. (2020). A robust tripeptide for in-field selective naked eye ultratrace detection of 2,4,6-trinitrophenol. Cryst. Growth Des..

[bib77] Ozay H., Gungor Z., Yilmaz B., Ilgin P., Ozay O. (2020). Dual use of colorimetric sensor and selective copper removal from aqueous media with novel p(HEMA-co-TACYC) hydrogels: Cyclen derivative as both monomer and crosslinker. J. Hazard. Mater..

[bib78] Pang C.-M., Luo S.-H., Jiang K., Wang B.-W., Chen S.-H., Wang N., Wang Z.-Y. (2019). A dual-channel sensor containing multiple nitrogen heterocycles for the selective detection of Cu^2+^, Hg^2+^ and Zn^2+^ in same solvent system by different mechanism. Dyes Pigm..

[bib79] Park H., Kim J.W., Hong S.Y., Lee G., Kim D.S., Oh J.H., Yun J.Y., Ha J.S. (2018). Microporous polypyrrole-coated graphene foam for high-performance multifunctional sensors and flexible supercapacitors. Adv. Funct. Mater..

[bib80] Park K.-J., Gong M.-S. (2017). A water durable resistive humidity sensor based on rigid sulfonated polybenzimidazole and their properties. Sens. Actuators B.

[bib81] Podasca V.E., Chibac A.L., Buruiana E.C. (2019). Fluorescence quenching study of a block copolymer with uracil end units by means of nitroaromatic derivatives and metal cations. J. Mol. Struct..

[bib82] Qian J.N., Wu D., Cai P., Xia J.B. (2019). Nitrogen atom free polythiophene derivative as an efficient chemosensor for highly selective and sensitive Cu^2+^ and Ag^+^ detection. Spectrochim. Acta A.

[bib83] Qu P., Li Y.C., Huang H.Y., Chen J.J., Yu Z.B., Huang J., Wang H.L., Gao B. (2020). Urea formaldehyde modified alginate beads with improved stability and enhanced removal of Pb^2+^, Cd^2+^ and Cu^2+^. J. Hazard. Mater..

[bib84] Rabbani M.G., Islamoglu T., El-Kaderi H.M. (2017). Benzothiazole-and benzoxazole-linked porous polymers for carbon dioxide storage and separation. J. Mater. Chem. A.

[bib85] Rajak R., Saraf M., Verma S.K., Kumar R., Mobin S.M. (2019). Dy(III)-based metal- organic framework as a fluorescent probe for highly selective detection of picric acid in aqueous medium. Inorg. Chem..

[bib86] Rajalakshmi A.V., Palanisami N. (2020). Y-shaped ferrocene/non-ferrocene conjugated quino- xalines for colorimetric and fluorimetric detection of picric acid. Spectrochim. Acta A.

[bib87] Ran Y., Wang S.Y., Yin Q.Y., Wen A.L., Peng X.X., Long Y.F., Chen S. (2020). Green synthesis of fluorescent carbon dots using chloroplast dispersions as precursors and application for Fe^3+^ ion sensing. Luminescence.

[bib88] Roja S.S., Shylaja A., Kumar R.R. (2020). Phenothiazine-tethered 2-aminopyridine-3-carbo- nitrile: fluorescent turn-off chemosensor for Fe^3+^ ions and picric acid. ChemistrySelect.

[bib89] Schmittel M., Shu Q.H. (2012). A lab-on-a-molecule for anions in aqueous solution: using Kolbe electrolysis and radical methylation at iridium for sensing. Chem. Commun..

[bib135] Seo S., Kim J., Jang G., Kim D., Lee T.S. (2014). Aggregation-deaggregation-triggered, tunable fluorescence of an assay ensemble composed of anionic conjugated polymer and polypeptides by enzymatic catalysis of trypsin. ACS Appl. Mater. Interfaces.

[bib90] Seo J.-M., Noh H.-J., Jeong H.Y., Baek J.-B. (2019). Converting unstable imine-linked network into stable aromatic benzoxazole-linked one *via* post-oxidative cyclization. J. Am. Chem. Soc..

[bib91] Shan M.X., Liu X.L., Wang X.R., Liu Z.L., Iziyi H., Ganapathy S., Gascon J., Kapteijn F. (2019). Novel high performance poly(p-phenylene benzobisimidazole) (PBDI) membranes fabricated by interfacial polymerization for H_2_ separation. J. Mater. Chem. A.

[bib92] Sharma A.K., Priya, Kaith B.S., Isha, Singh A., Chandel K., Vipula (2019). Riboflavin functionalized dextrin-sodium alginate based fluorescent sensor: detoxification of Cu^2+^ and Ni^2+^ ions. ACS Appl. Polym. Mater..

[bib93] Shia W.G., Lu X.Y., Zhang S.S., Li H.W., Liu M., Dong B. (2020). C=N based PAMAM polymer dots: fluorescent property and Cu^2+^ sensing application. Colloids Surf. A.

[bib94] Slenders E., Castello M., Buttafava M., Villa F., Tosi A., Lanzanò L., Koho S.V., Vicidomini G. (2021). Confocal-based fluorescence fluctuation spectroscopy with a SPAD array detector. Light Sci. Appl..

[bib95] Sun R.X., Feng S.Y., Zhou B.Y., Chen Z.X., Wang D.X., Liu H.Z. (2020). Flexible cyclosiloxane-linked fluorescent porous polymers for multifunctional chemical sensors. ACS Macro Lett..

[bib96] Thanzeel F.Y., Balaraman K., Wolf C. (2020). Quantitative chirality and concentration sensing of alcohols, diols, hydroxy acids, amines and amino alcohols using chlorophosphite sensors in a relay assay. Angew. Chem. Int. Ed..

[bib97] Tian X.-M., Yao S.-L., Wu J., Xie H.M., Zheng T.-F., Jiang X.-J., Wu Y.Q., Mao J.G., Liu S.-J. (2019). Two benzothiadiazole-based fluorescent sensors for selective detection of Cu^2+^ and OH^-^ ions. Polyhedron.

[bib98] To K.C., Ben-Jaber S., Parkin I.P. (2020). Recent developments in the field of explosive trace detection. ACS Nano.

[bib99] Wang B.-W., Jiang K., Li J.-X., Luo S.-H., Wang Z.-Y., Jiang H.F. (2020). 1,1-Diphenylvinylsulfide as a functional AIEgen derived from the aggregation-caused- quenching molecule 1,1-diphenylethene through simple thioetherification. Angew. Chem. Int. Ed..

[bib100] Wang Q.R., Zheng C.L., Cui W., He F., Zhang J.Y., Zhang T.C., He C. (2020). Adsorption of Pb^2+^ and Cu^2+^ ions on the CS_2_-modified alkaline lignin. Chem. Eng. J..

[bib101] Wang X.R., Shan M.X., Liu X.L., Wang M., Doherty C.M., Osadchii D., Kapteijn F. (2019). High-performance polybenzimidazole membranes for helium extraction from natural gas. ACS Appl. Mater. Inter..

[bib102] Wang Y., Zhang L., Yang L., Chang G.J. (2020). An indole-based smart aerogel for simultaneous visual detection and removal of trinitrotoluene in water *via* synergistic effect of dipole-π and donor-acceptor interactions. Chem. Eng. J..

[bib103] Wei C., Ding P., Nie X.R., Stuart M.C.A., Wang J.Y. (2020). Europium based coordination polyelectrolytes enable core-shell-corona micelles as luminescent probes. Soft Matter.

[bib105] Wu K., Hu J.S., Cheng X.F., Li J.X., Zhou C.H. (2020). A superior luminescent metal-organic framework sensor for sensing trace Al^3+^ and picric acid *via* disparate charge transfer behaviors. J. Lumin..

[bib108] Wu Z.-F., Velasco E., Shan C., Tan K., Zhang Z.-Z., Hu Q.-Q., Xing K., Huang X.-Y., Li J. (2020). Robust fluorescent calcium coordination polymers as Cu^2+^ sensors with high sensitivity and fast response. J. Mater. Chem. C.

[bib106] Wu Y.-C., Huo J.-P., Cao L., Ding S., Wang L.-Y., Cao D.R., Wang Z.-Y. (2016). Design and application of tribenzimidazolyl star-shape molecules as fluorescent chemosensors for the fast-response detection of fluoride ion. Sens. Actuators, B.

[bib107] Wu Y.-C., You J.-Y., Jiang K., Wu H.-Q., Xiong J.-F., Wang Z.-Y. (2018). Novel benzimidazole-based ratiometric fluorescent probes for acidic pH. Dyes Pigm..

[bib109] Xiao L.Q., Shi J., Nan B.F., Chen W.L., Zhang Q., Zhang E.D., Lu M.G. (2020). Highly sensitive detection of Fe^3+^ ions using waterborne polyurethane-carbon dots self-healable fluorescence film. Macromol. Mater. Eng..

[bib110] Xie Y.J., Ge Y.W., Peng Q., Li C.G., Li Q.Q., Li Z. (2017). How the molecular packing affects the room temperature phosphorescence in pure organic compounds: the ingeniously molecular design, detailed crystal analysis, and rational theoretical calculations. Adv. Mater..

[bib111] Xiong S.Y., Marin L., Duan L., Cheng X.J. (2019). Fluorescent chitosan hydrogel for highly and selectively sensing of pnitrophenol and 2,4,6-trinitrophenol. Carbohydr. Polym..

[bib112] Xu T.-Y., Wang H., Li J.-M., Zhao Y.-L., Han Y.-H., Wang X.-L., He K.-H., Wang A.-R., Shi Z.-F. (2019). A water-stable luminescent Zn(II) coordination polymer based on 5-sulfosalicylic acid and 1,4-bis(1H-imidazol-1-yl)benzene for highly sensitive and selective sensing of Fe^3+^ ion. Inorg. Chim. Acta.

[bib113] Xu X.-Y., Lian X., Hao J.-N., Zhang C., Yan B. (2017). A double-stimuli-responsive fluorescent center for monitoring of food spoilage based on dye covalently modified EuMOFs: from sensory hydrogels to logic devices. Adv. Mater..

[bib114] Yang J., Ren Z.C., Xie Z.L., Wang C., Xie Y.J., Peng Q., Chi Z.G., Li Q.Q., Li Z. (2017). The first AIEgen with fluorescence-phosphorescence dual Mechanoluminescent at room temperature. Angew. Chem. Int. Ed..

[bib115] Yin G.-X., Niu T.-T., Gan Y.-B., Yu T., Yin P., Chen H.-M., Zhang Y.-Y., Li H.-T., Yao S.-Z. (2018). A multi-signal fluorescent probe with multiple binding sites for simultaneous sensing of cysteine, homocysteine, and glutathione. Angew. Chem. Int. Ed..

[bib116] Yu S.J., Li W., Fujii Y.K., Omuraa T., Minami H. (2019). Fluorescent spherical sponge cellulose sensors for highly selective and semiquantitative visual analysis: detection of Hg^2+^ and Cu^2+^ ions. ACS Sustain. Chem. Eng..

[bib117] Zeng G., Xing S.H., Wang X.R., Yang Y.L., Ma D.X., Liang H.W., Gao L., Hua J., Li G.H., Shi Z. (2016). 3d-4f Metal-organic framework with dual luminescent centers that efficiently discriminates the isomer and homologues of small organic molecules. Inorg. Chem..

[bib120] Zhang H., Dong X.Z., Wang J.H., Guan R.F., Cao D.X., Chen Q.F. (2019). Fluorescence emission of polyethylenimine-derived polymer dots and its application to detect copper and hypochlorite ions. ACS Appl. Mater. Inter..

[bib123] Zhang M.L., Zheng Y.J., Liu M., Ren Y.X., Wang Z.X., Cao J., Wang J.J. (2019). Two Cd(II)/Mn(II) coordination polymers showing dual responsive fluorescence sensing for Fe^3+^ and *o*-NAL. J. Solid State Chem..

[bib122] Zhang L., Yu S., Liang X.Y., Meng X.M., Zhang Q., Li P.W., Zhou Y. (2020). Cysteamine triggered “turn-on” fluorescence sensor for total detection of fumonisin B_1_, B_2_ and B_3_. Food Chem..

[bib124] Zhang R.Q., Hu L.P., Xu Z.X., Song Y.X., Li H.Q., Zhang X., Gao X.C., Wang M.X., Xian C.Y. (2020). A highly selective probe for fluorescence turn-on detection of Fe^3+^ ion based on a novel spiropyran derivative. J. Mol. Struct..

[bib126] Zhang Y.-M., Zhu W., Huang X.-J., Qu W.-J., He J.-X., Fang H., Yao H., Wei T.-B., Lin Q. (2018). Supramolecular AIE gels based on pillar[5]arene for ultrasensitive detection and separation of multi-analytes. ACS Sustain. Chem. Eng..

[bib127] Zhao Y.J., Peng D.F., Bai G.X., Huang Y.Q., Xu S.Q., Hao J.H. (2021). Multiresponsive emissions in luminescent ions doped quaternary piezophotonic materials for mechanical -to-optical energy conversion and sensing applications. Adv. Funct. Mater..

[bib128] Zhao Y.T., Ouyang H., Feng S., Luo Y.N., Shi Q.R., Zhu C.Z., Chang Y.-C., Li L., Du D., Yang H.P. (2019). Rapid and selective detection of Fe(III) by using a smartphone-based device as a portable detector and hydroxyl functionalized metal organic frameworks as the fluorescence probe. Anal. Chim. Acta.

[bib129] Zheng L.B., Qi P., Zhang D. (2018). Identification of bacteria by a fluorescence sensor array based on three kinds of receptors functionalized carbon dots. Sens. Actuators B.

[bib130] Zheng Y.T., Wang H.L., Jiang J.Z. (2020). A porous tetraphenylethylene-based polymer for fast-response fuorescence sensing of Fe(III) ion and nitrobenzene. Dyes Pigm..

[bib131] Zhu H., Fu L.S., Liu D., Li Y.-H., Dong G.-Y. (2020). Three water-stable luminescent Zn(II) coordination polymers for highly sensitive and selective sensing of acetylacetone and Fe^3+^ ions. J. Solid State Chem..

[bib132] Zhuang X.R., Zhang N.X., Zhang X., Wang Y., Zhao L.Y., Yang Q.F. (2020). A stable Cu- MOF as a dual function sensor with high selectivity and sensitivity detection of picric acid and CrO_4_^2-^ in aqueous solution. Microchem. J..

[bib133] Zhuang Y.P., Yao J.Y., Zhuang Z.Y., Ni C.J., Yao H.M., Su D.L., Zhou J., Zhao Z.J. (2020). Influence of alkyl chains on fluoranthene ensembles towards fluorescence based detection of 2,4,6-trinitrophenol. AEE-active conjugated polymers based on di(naphthalen-2- yl)-1,2-diphenylethene for sensitive fluorescence detection of picric acid. Dyes Pigm..

