# Supplementary material for: N-alkylation briefly constructs tunable multifunctional sensor materials: Multianalyte detection and reversible adsorption
Source: iScience. 2021 Sep 14;24(10):103126. doi: 10.1016/j.isci.2021.103126 (PMC8487030; doi:10.1016/j.isci.2021.103126)
Supplement: Document S1. Figures S1–S54 and Tables S1–S9 [file mmc1.pdf]

iScience, Volume *24*

Supplemental information

N-alkylation briefly constructs tunable multifunctional sensor materials: Multianalyte detection and reversible adsorption

Chu-Ming Pang, Xi-Ying Cao, Ying Xiao, Shi-He Luo, Qi Chen, Yong-Jun Zhou, and Zhao-Yang Wang

Supplemental Information

N-Alkylation Briefly Constructs Tunable Multifunctional Sensor Materials: Multianalyte Detection and Reversible Adsorption

Chu-Ming Pang[#1,2], Xi-Ying Cao[#1], Ying Xiao[1], Shi-He Luo[*1,3], Qi Chen[1], Yong-Jun Zhou[1] and Zhao-Yang Wang[*1,3,4]

[1] *School of Chemistry, South China Normal University, Key Laboratory of Theoretical Chemistry of Environment, Ministry of Education; Guangzhou Key Laboratory of Analytical Chemistry for Biomedicine, Guangzhou 510006, P. R. China. Email: wangzy@scnu.edu.cn; pinky_r@163.com*

[2] *School of Health Medicine, Guangzhou Huashang College, Guangzhou 511300, P. R. China*

[3] *Key Laboratory of Functional Molecular Engineering of Guangdong Province, School of Chemistry and Chemical Engineering, South China University of Technology, Guangzhou 510641, P.R. China*

[4] *Lead Contact*

[#] *The authors contributed equally to this work.*

Contents

1. Characterization spectra of **PBI** and serial **SPBIs** (**Figures S1-S8**)..3
2. The ^{1}H NMR spectra of **SPBIs** with different molar feed ratios (**Figure S9**)......................................11
3. The FT-IR spectra of **SPBIs** with different molar feed ratios (**Figure S10**)..12
4. ^{1}HNMR and FT-IR data of **PBI** and serial **SPBIs** (**Tables S1-S2**)…..........……………………………13
5. The full XPS spectra of **SPBI-c** and its C1s, N1s peaks (**Figure S11**)...15
6. The effects of different feed ratios on serial **SPBIs** (**Table S3**)..16
7. The XRD analysis of **SPBIs** with different molar feed ratios (**Figure S12**)......................................17
8. TGA analysis of serial **SPBIs** with different molar feed ratios (**Figure S13**).....................................18
9. The thermal decomposition temperatures of **SPBI** with different feed ratios (**Table S4**)...................19
10. UV-*vis* absorption and fluorescent spectra of **SPBI** in different solvents (**Figures S14-S16**)...........20
11. The competitive experiments of **SPBI-a** for Cu^{2+} or Fe^{3+} (**Figure S17**)..22
12. UV-*vis* absorption spectra of **SPBI-a** and absorbance changes at 275, 303 and 605 nm upon the addition of Cu^{2+} (**Figure S18**)..23
13. The plot of sensor **SPBI-a** *vs* different concentration of Cu^{2+} (**Figure S19**)....................................24
14. The selective experiments of **SPBI-b** towards metal ions (**Figure S20**)...25

15. The competitive experiments of **SPBI-b** for Cu^{2+} or Fe^{3+} (**Figures S21-S22**)..................................26
16. UV-*vis* absorption spectra of probe **SPBI-b** upon the addition of Cu^{2+} (**Figure S23**)........................28
17. The plot of A_{605}/A_{303} of probe **SPBI-b** *vs* different concentration of Cu^{2+} (**Figure S24**)..................29
18. UV-*vis* absorption spectra of probe **SPBI-b** upon the addition of Fe^{3+} (**Figure S25**)........................30
19. The plot of A_{385} of probe **SPBI-b** *vs* different concentration of Fe^{3+} (**Figure S26**)...........................31
20. The selective experiments of **SPBI-c** towards metal ions (**Figure S27**)...32
21. The competitive experiments of **SPBI-c** for Cu^{2+} or Fe^{3+} (**Figures S28-S29**)..................................33
22. The titration experiments of **SPBI-c** to Cu^{2+} or Fe^{3+} (**Figure S30**)..35
23. The absorbance changes of **SPBI-c** with the addition of Cu^{2+} or Fe^{3+} (**Figure S31**).........................36
24. The fluorescence titration experiment of **SPBI-c** towards Cu^{2+} (**Figure S32**)..................................37
25. The plot of absorbance of probe **SPBI-c** *vs* concentration of Cu^{2+} or Fe^{3+} (**Figure S33**)..................38
26. The selective experiments of **SPBI-g** towards metal ions (**Figure S34**)...39
27. The competitive experiments of **SPBI-g** for Cu^{2+} or Fe^{3+} (**Figures S35-S36**)..................................40
28. UV-*vis* absorption spectra of **SPBI-g** upon the addition of Cu^{2+} or Fe^{3+} (**Figures S37-S38**)...........41
29. The plot of the absorbance of **SPBI-g** *vs* concentrations of Cu^{2+} or Fe^{3+} (**Figure S39**)....................42
30. The reversible adsorption capacity of **SPBI** to Cu^{2+} (**Figure S40** and **Table S5**)..............................43
31. Structures of the tested nitroaromatic compounds (**Figure S41**)..45
32. The selective experiments of **SPBI-c** towards NACs (**Figure S42**)..46
33. The plots of **SPBI-c** *vs* concentration of DNP or NP (**Figure S43**)..47
34. The selective and titration experiments of **SPBI-g** towards NACs (**Figures S44-S45**)....................48
35. The linear plots and Stern-Volmer plots of **SPBI-g** *vs* concentration of NACs (**Figures S46-S47**)..49
36. The changes of FT-IR spectra and morphology before or after the combination with analytes (**Figures S48-S52**)..50
37. TCSPC plots for **SPBI-c** and **SPBI-g** interacted with NACs (**Figure S53**).......................................53
38. The optimized geometries of **SPBI-c**, its Cu^{2+} complex and PA (**Figure S54**)..................................54
39. Comparison of **SPBI** with Cu^{2+}, Fe^{3+} and PA probes available in the literature (**Tables S6-S8**)......55
40. The adsorption comparison of **SPBI** with the reported Cu^{2+} adsorption material (**Table S9**)...........61

1. Characterization spectra of PBI and serial SPBIs

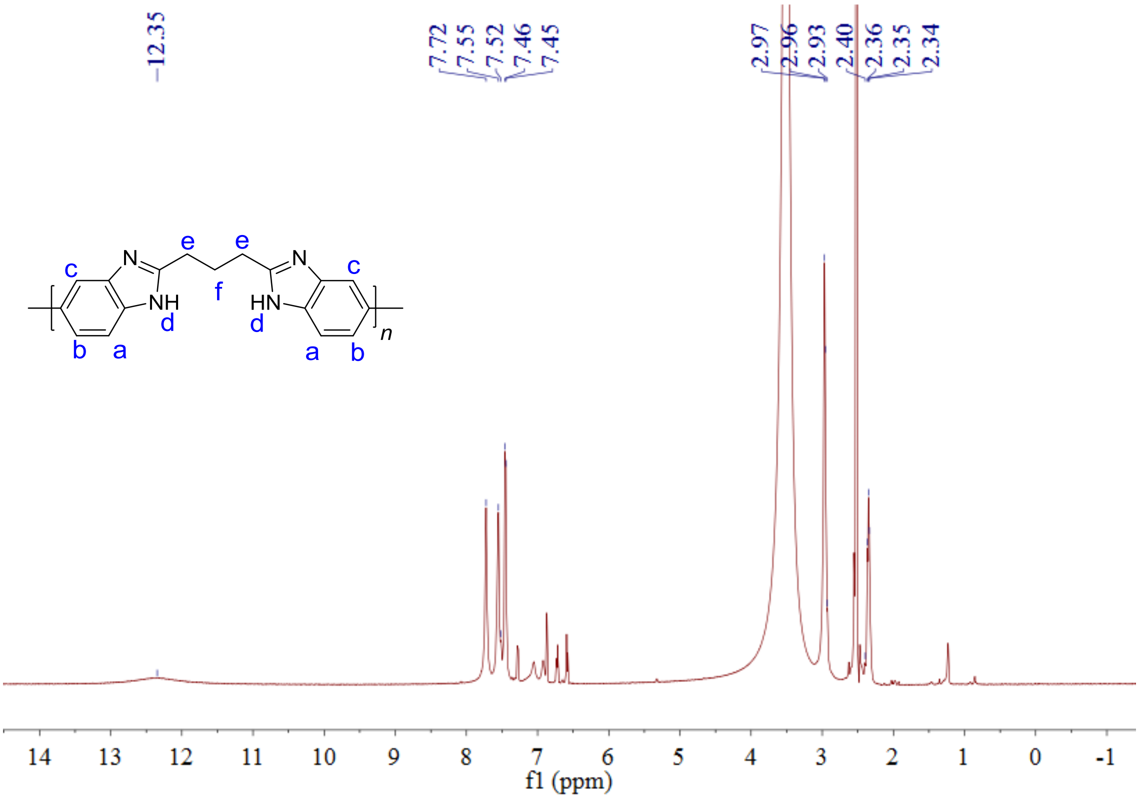

Figure S1. ^{1}H NMR spectrum of **PBI**. Related to **Figure 2** and **Table 1**.

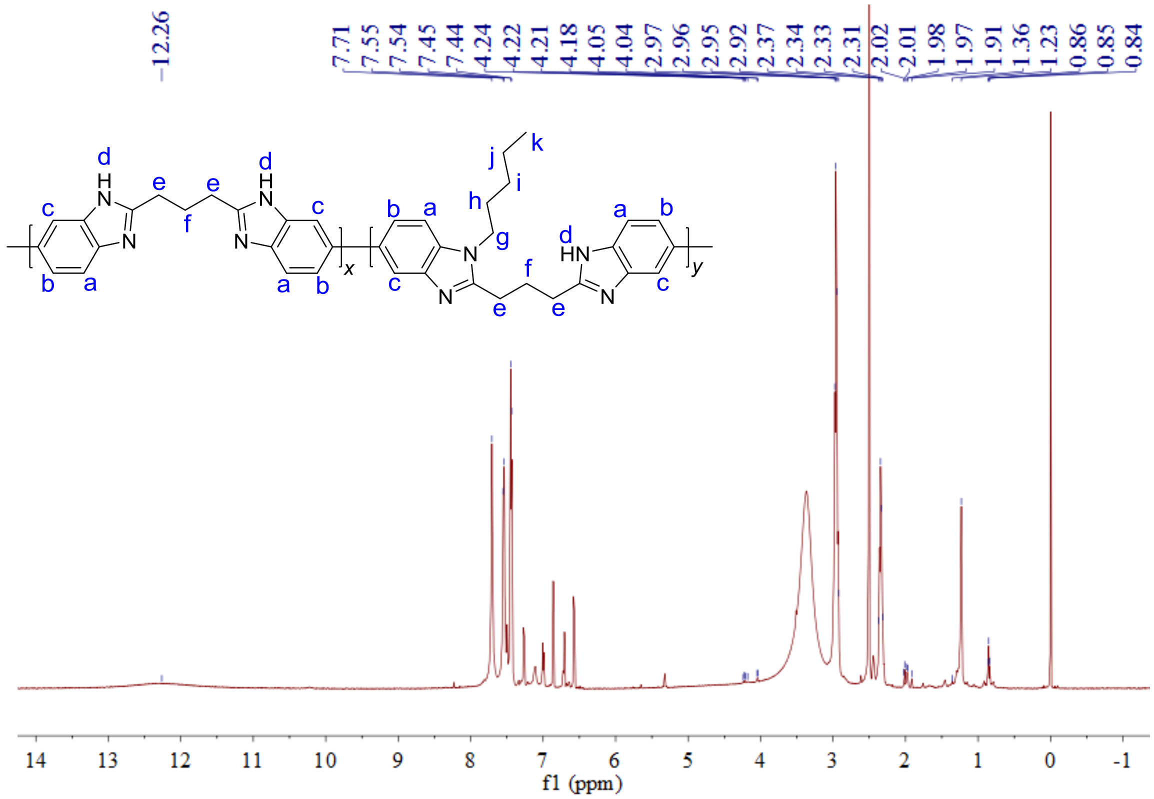

Figure S2. ^{1}H NMR spectrum of **SPBI-a**. Related to **Figure 2** and **Table 1**.

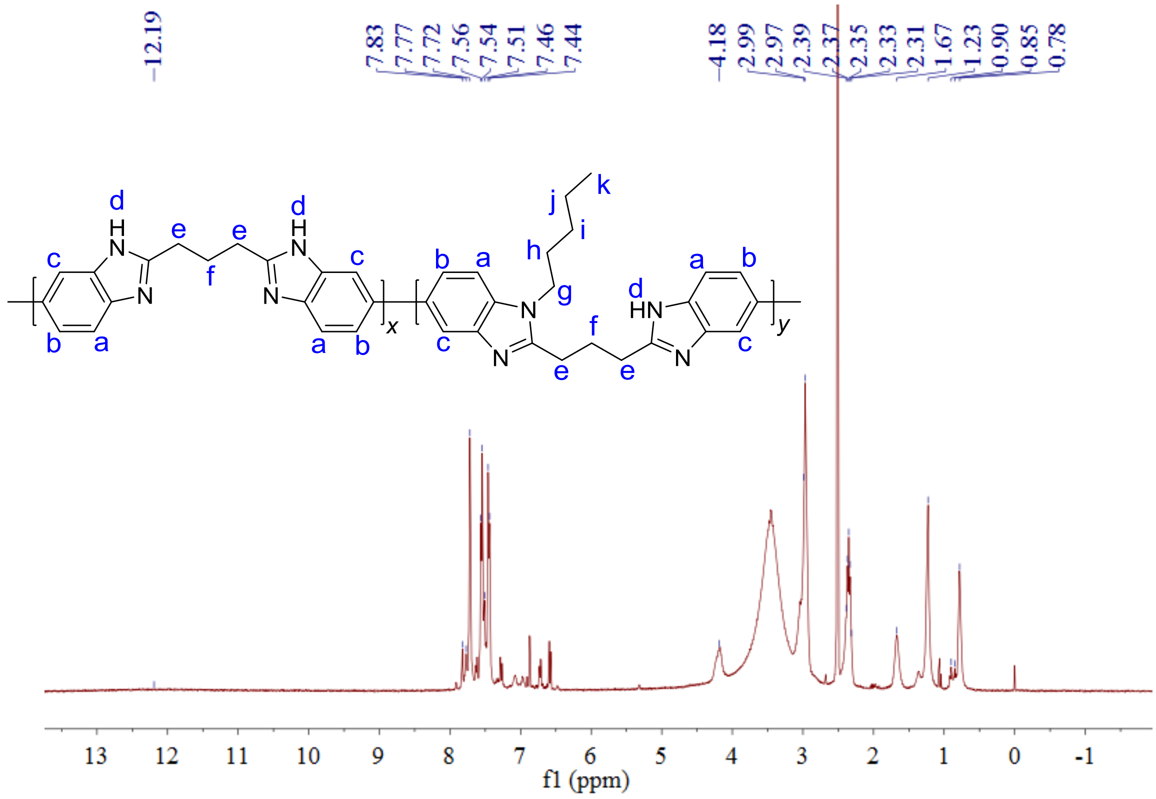

Figure S3. ^{1}H NMR spectrum of **SPBI-b**. Related to **Figure 2** and **Table 1**.

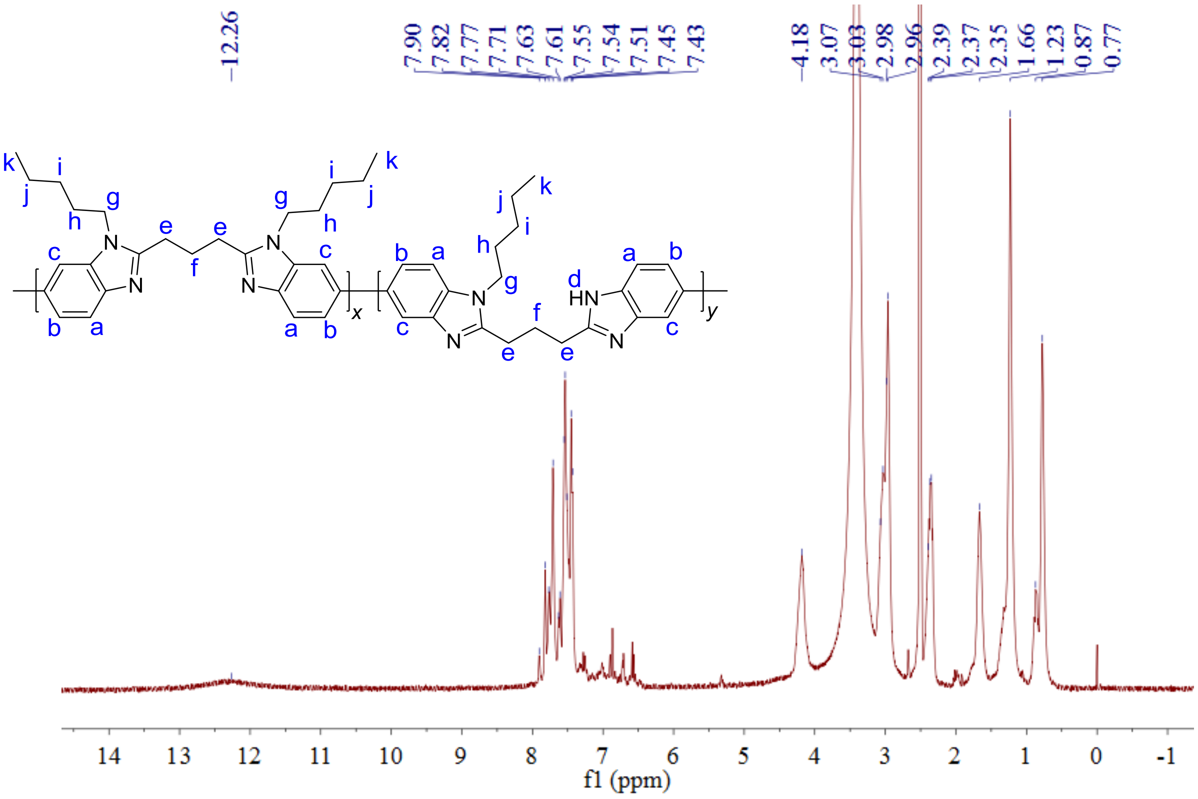

Figure S4. ^{1}H NMR spectrum of **SPBI-c**. Related to **Figure 2** and **Table 1**.

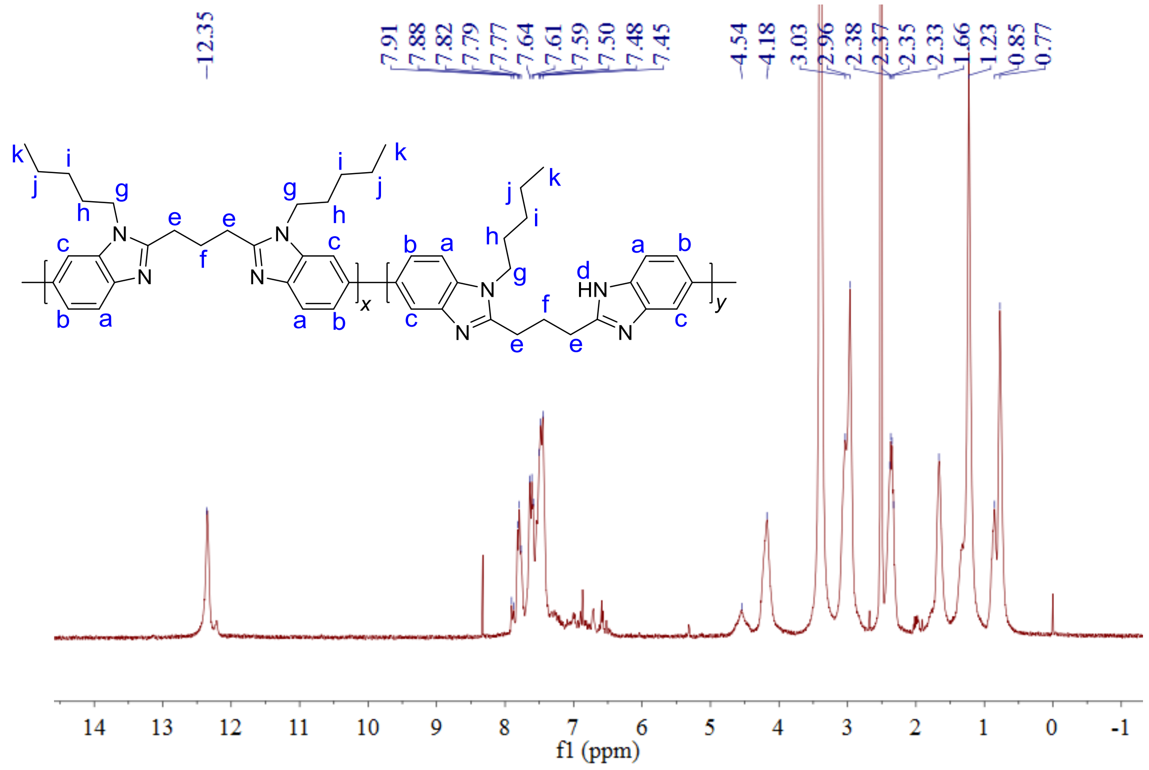

Figure S5. ^{1}H NMR spectrum of **SPBI-d**. Related to **Figure 2** and **Table 1**.

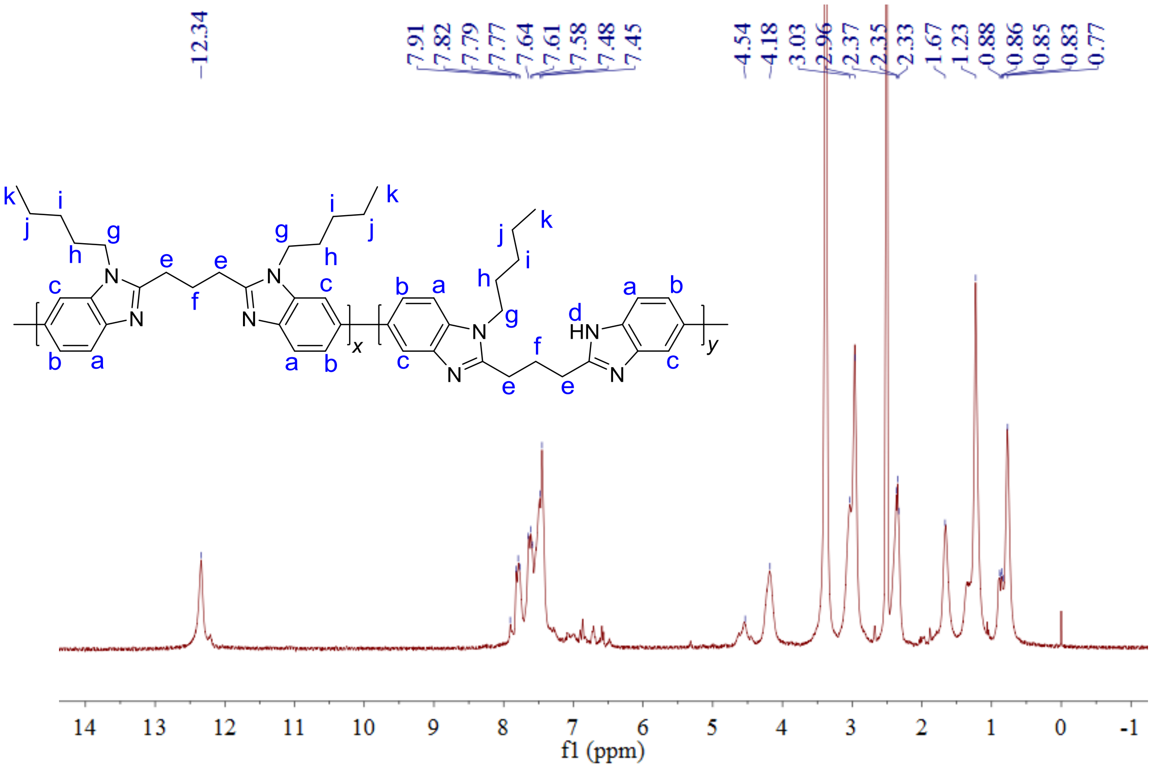

Figure S6. ^{1}H NMR spectrum of **SPBI-e**. Related to **Figure 2** and **Table 1**.

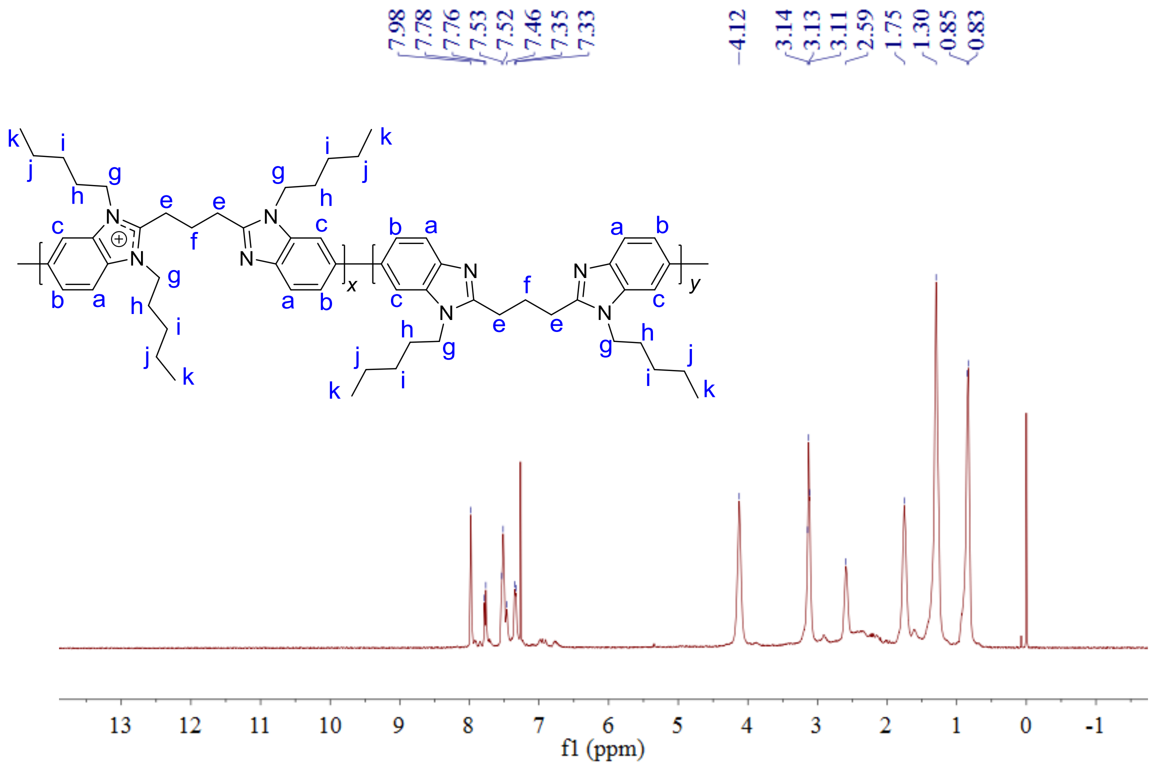

Figure S7. ^{1}H NMR spectrum of **SPBI-f**. Related to **Figure 2** and **Table 1**.

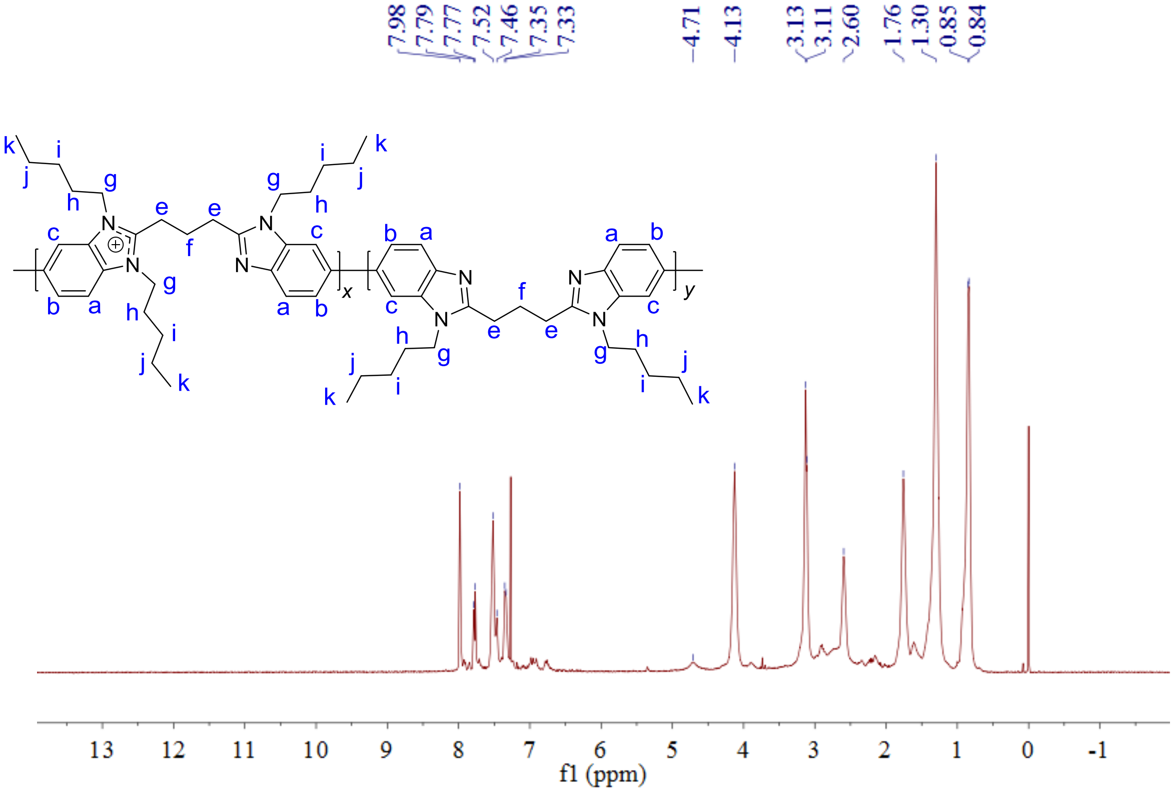

Figure S8. ^{1}H NMR spectrum of **SPBI-g**. Related to **Figure 2** and **Table 1**.

2. The ^{1}H NMR spectra of SPBIs with different molar feed ratios

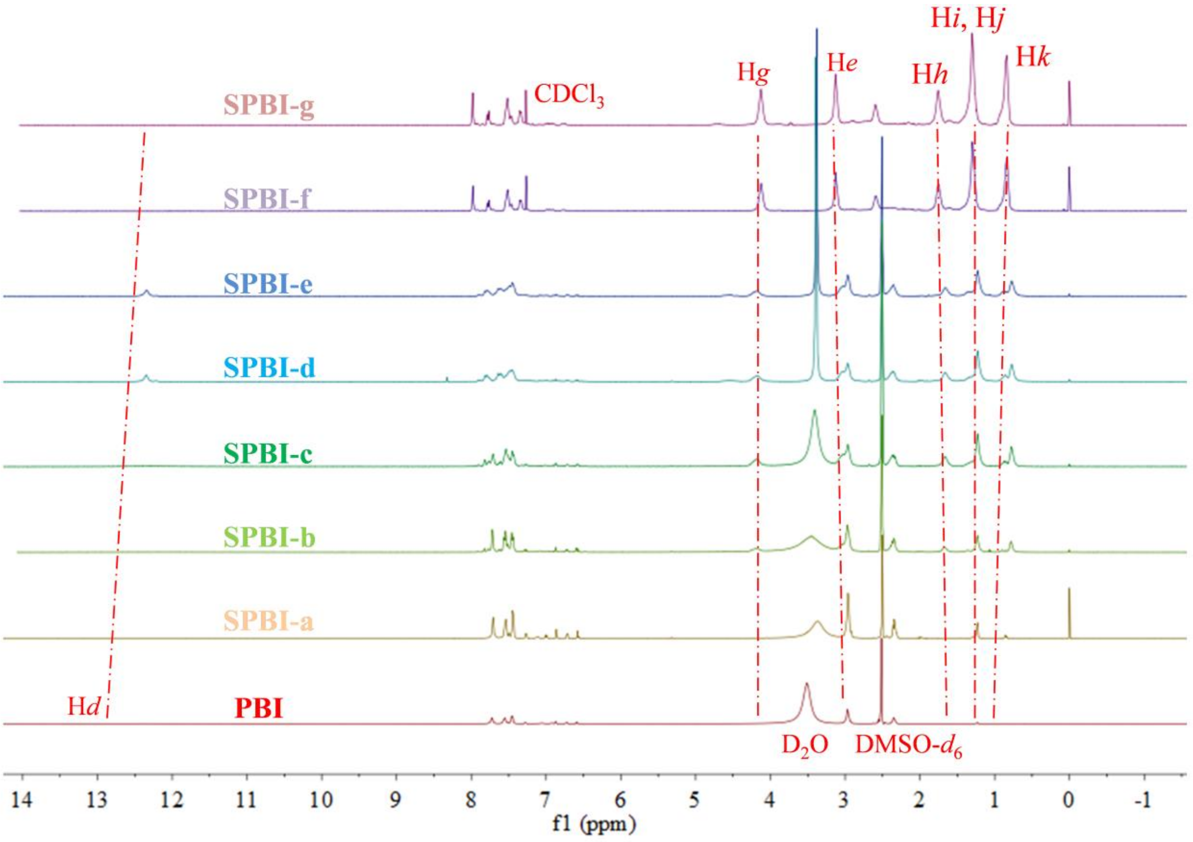

Figure S9. The changes of ^{1}H NMR spectra of **PBI**, and **SPBIs** with different molar feed ratios [$n(C_5H_{11}Br)/n$(**PBI**)]. Related to **Figure 2** and **Table 1**.

3. The FT-IR spectra of SPBIs with different molar feed ratios

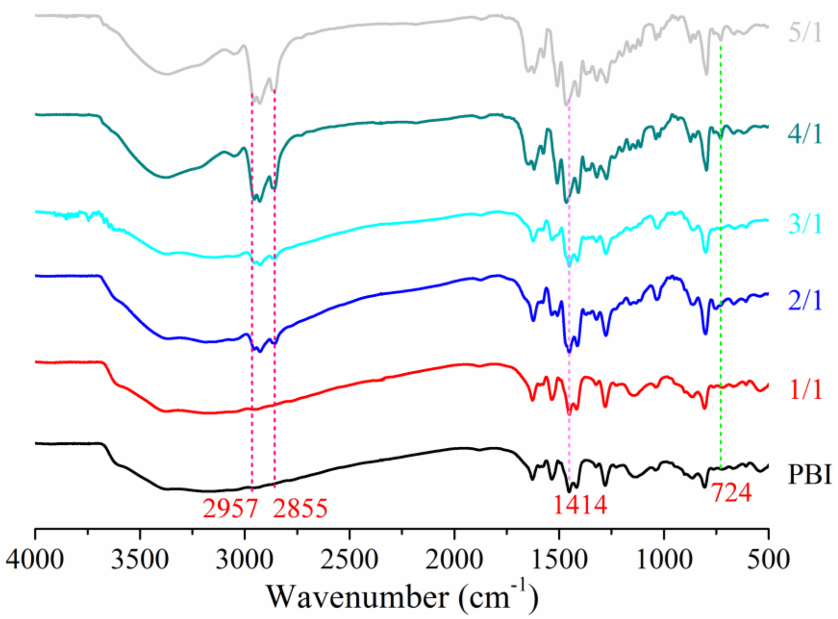

Figure S10. The FT-IR spectra of **SPBIs** with different molar feed ratios [$n(C_5H_{11}Br)/n$(**PBI**)]. Related to **Figure 2** and **Table 1**.

4. ^{1}H NMR and FT-IR data of PBI and serial SPBIs

Table S1. The ^{1}H NMR data of **PBI** and serial **SPBIs**. Related to **Figure 2** and **Table 1**.

Sample	δ (ppm)									
	**H$_k$**	**H$_{i,j}$**	**H$_h$**	**H$_f$**	**H$_e$**	**H$_g$**	**H$_a$**	**H$_b$**	**H$_c$**	**H$_d$**
**PBI**	-	-	-	2.31-2.36	2.92-2.98	-	7.41-7.48	7.51-7.61	7.65-7.82	12.35
**SPBI-a**	0.76-0.92	1.16-1.30	1.89-2.02	2.31-2.36	2.94-2.98	4.03-4.26	7.43-7.46	7.51-7.57	7.67-7.74	12.26
**SPBI-b**	0.68-0.90	1.13-1.31	1.60-1.75	2.30-2.43	2.93-3.05	4.07-4.32	7.39-7.49	7.51-7.63	7.68-7.84	12.19
**SPBI-c**	0.69-0.90	1.10-1.32	1.57-1.75	2.26-2.43	2.94-3.11	4.08-4.28	7.36-7.50	7.52-7.65	7.69-7.90	12.26
**SPBI-d**	0.64-0.93	1.11-1.32	1.53-1.81	2.25-2.44	2.91-3.12	4.10-4.50	7.31-7.47	7.49-7.63	7.65-7.98	12.35
**SPBI-e**	0.60-0.93	0.99-1.36	1.51-1.81	2.30-2.44	2.91-3.06	4.10-4.52	7.39-7.49	7.53-7.65	7.68-7.93	12.34
**SPBI-f**	0.72-0.97	1.20-1.42	1.64-1.95	2.50-2.67	3.03-3.28	3.91-4.32	7.30-7.46	7.48-7.64	7.73-8.01	-
**SPBI-g**	0.71-1.00	1.18-1.42	1.64-1.95	2.50-2.65	3.05-3.22	4.10-4.72	7.30-7.48	7.49-7.62	7.71-7.98	-

Table S2. The FT-IR data of **PBI** and **SPBIs**. Related to **Figure 2** and **Table 1**.

**Sanples**	**IR** (KBr), *v*, cm^{-1}
**PBI**	3376, 3045, 1628, 1537, 1450, 1415, 1278, 1127, 862, 807
**SPBI-a**	3361, 3045, 2955, 2910, 2839, 1624, 1590, 1538, 1442, 1442, 1409, 1281, 857, 804, 721
**SPBI-b**	3361, 3032, 2961, 2929, 2852, 1622, 1577, 1532, 1448, 1448, 1409, 1275, 856, 792, 728
**SPBI-c**	3366, 3030, 2941, 2871, 1624, 1570, 1532, 1451, 1451, 1414, 1278, 866, 803, 724
**SPBI-d**	3373, 3051, 2961, 2935, 2858, 1610, 1538, 1506, 1448, 1448, 1416, 1275, 869, 798, 721
**SPBI-e**	3367, 3039, 2961, 2935, 2845, 1622, 1564, 1532, 1436, 1436, 1409, 1281, 850, 798, 728
**SPBI-f**	3045, 2955, 2916, 2858, 1648, 1622, 1564, 1506, 1455, 1455, 1409, 1320, 1268, 869, 792, 728
**SPBI-g**	3045, 2957, 2931, 2855, 1652, 1620, 1578, 1507, 1464, 1464, 1410, 1276, 875, 796, 729

5. The full XPS spectra of SPBI-c and its C1s, N1s peaks

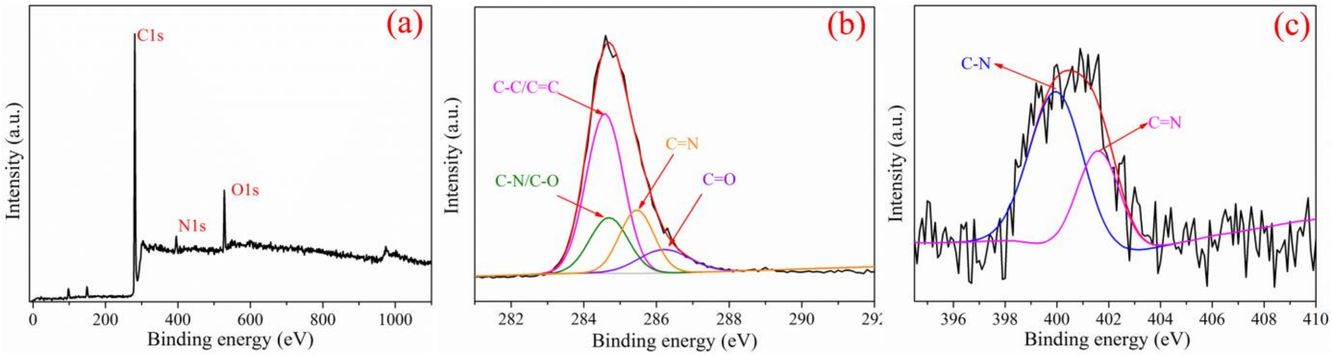

Figure S11. The full XPS spectra of **SPBI-c** (a) and its C1s (b), N1s (c) peaks. Related to **Figure 2** and **Table 1**.

6. The effects of different feed ratios on serial SPBIs

Table S3. The effects of different feed ratios on yield, color and actual alkylation rate of serial **SPBIs**. Related to **Figure 2** and **Table 1**.

Sample No.	Feed ratio (RBr/PBI)	Yield (%)	Appearance of product	Actual alkylation rate (%)	Mn (Da)
**SPBI-a**	0.1:1	25.6	Gray powder	6.0	1700
**SPBI-b**	0.5:1	26.2	Brown powder	17.0	1900
**SPBI-c**	1:1	51.0	Brown powder	28.5	2100
**SPBI-d**	2:1	54.6	Brown-red powder	34.5	2200
**SPBI-e**	3:1	62.9	Orange-red powder	38.0	2300
**SPBI-f**	4:1	65.3	Orange-yellow powder	59.3	2600
**SPBI-g**	5:1	86.1	Brown-red powder	65.0	2700

7. The XRD analysis of SPBIs with different molar feed ratios

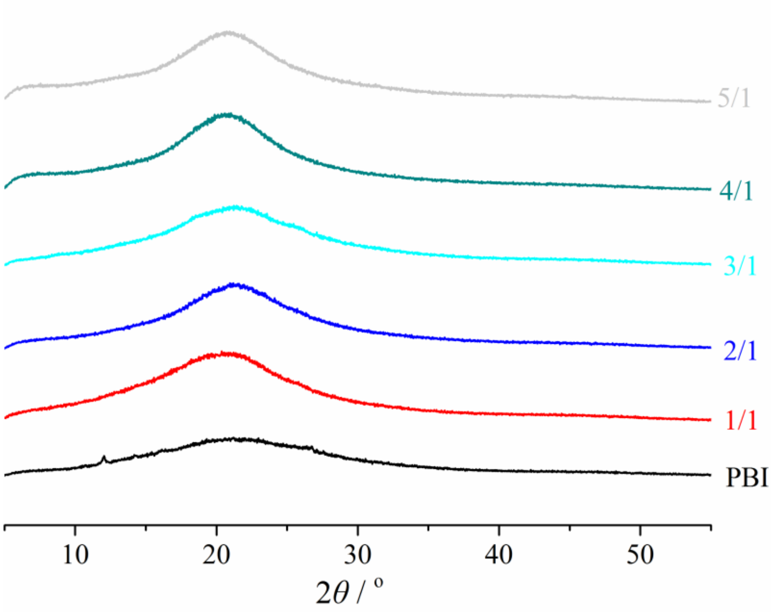

Figure S12. The XRD analysis of **SPBIs** with different molar feed ratios [$n(C_5H_{11}Br)/n$(**PBI**)].

Related to **Figure 2** and **Table 1**.

8. TGA analysis of serial SPBIs with different molar feed ratios

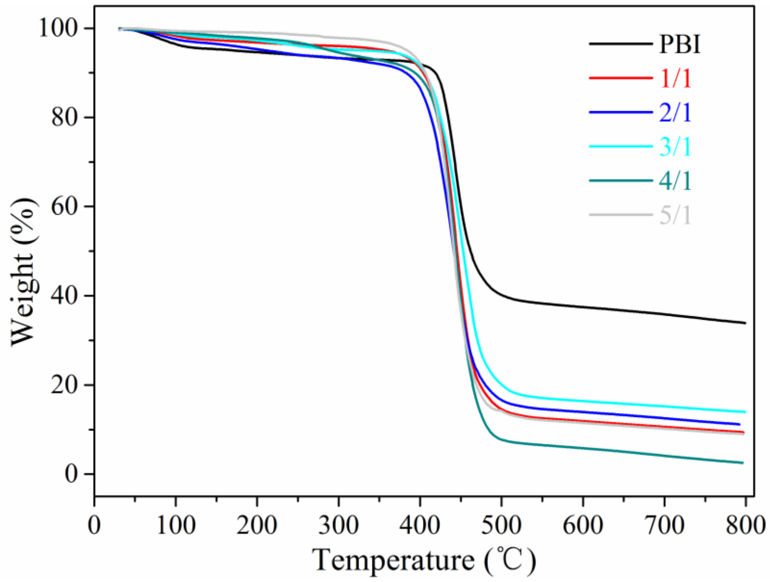

Figure S13. TGA analysis of serial **SPBIs** with different molar feed ratios [*n*($C_5H_{11}Br$)/*n*(**PBI**)]. Related to **Figure 2** and **Table 1**.

9. The thermal decomposition temperatures of SPBIs with different feed ratios

Table S4. The thermal decomposition temperatures of **SPBIs** with different feed ratios. Related to **Figure 2** and **Table 1**.

Feed ratio [$n(C_5H_{11}Br)/n$(**PBI**)]	Initial temperature (℃)	Terminal temperature (℃)	Thermo-gravimetric rate (%)
**PBI**	424.7	446.8	59.01
1:1	419.6	470.0	86.34
2:1	416.6	474.1	83.56
3:1	417.7	477.7	81.45
4:1	419.8	470.8	89.06
5:1	422.7	439.3	80.45

10. UV-*vis* absorption and fluorescent spectra of SPBI in different solvents

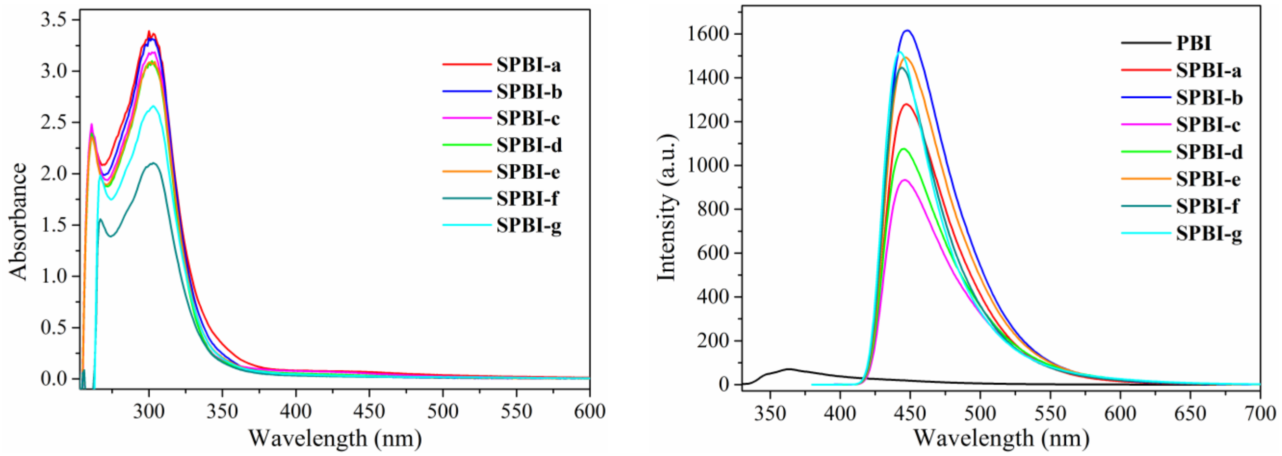

Figure S14. The UV-*vis* absorption spectra (left) and fluorescent spectra (right) of serial **SPBIs** with different molar feed ratios [$n(C_5H_{11}Br)/n$(**PBI**)] [With the increase of alkylation, the solubility of **SPBI** in polar solvent DMSO decreases. Thus, **PBI**, **SPBI-a** ~ **SPBI-e** are tested in DMSO, while the solvent for **SPBI-f** and **SPBI-g** is DMF instead; λ_{ex} = 328 nm, 339 nm, 336 nm, 331 nm, 329 nm, 332 nm, 327 nm, 329 nm for **SPBI-a** to **SPBI-g** respectively; λ_{em} = 447 nm (**SPBI-a**), 448 nm (**SPBI-b**), 447 nm (**SPBI-c**), 445 nm (**SPBI-d**), 447 nm (**SPBI-e**), 447 nm (**SPBI-f**), 443 nm (**SPBI-g**)]. Related to **Figure 4**.

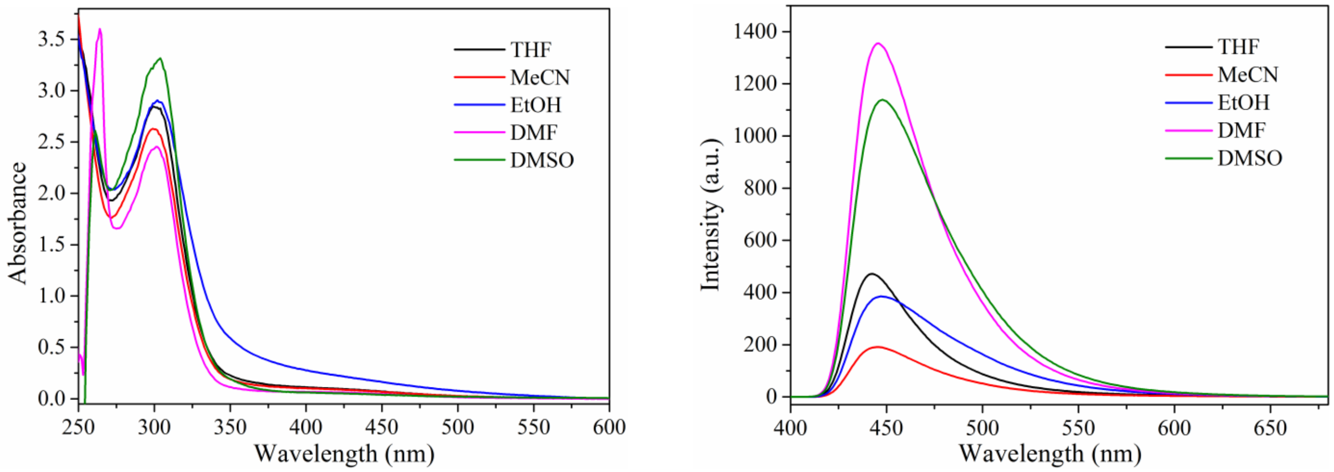

Figure S15. UV-*vis* absorption (left) and fluorescent (right) spectra of **SPBI-c** (1 mg / 15 mL) in solvents with different polarity (λ_{ex} = 331 nm, λ_{em} = 447 nm). Related to **Figure 4**.

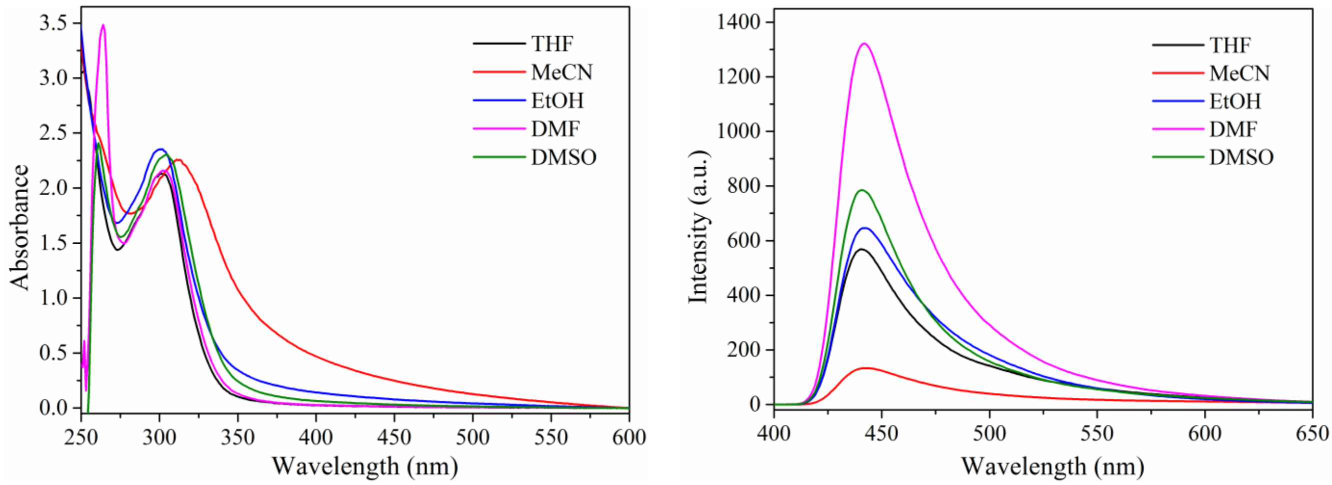

Figure S16. UV-*vis* absorption (left) and fluorescence (right) spectra of **SPBI-g** (1 mg / 15 mL) in solvents with different polarity (λ_{ex} = 329 nm, λ_{em} = 443 nm). Related to **Figure 4**.

11. The competitive experiments of SPBI-a for Cu^{2+} or Fe^{3+}

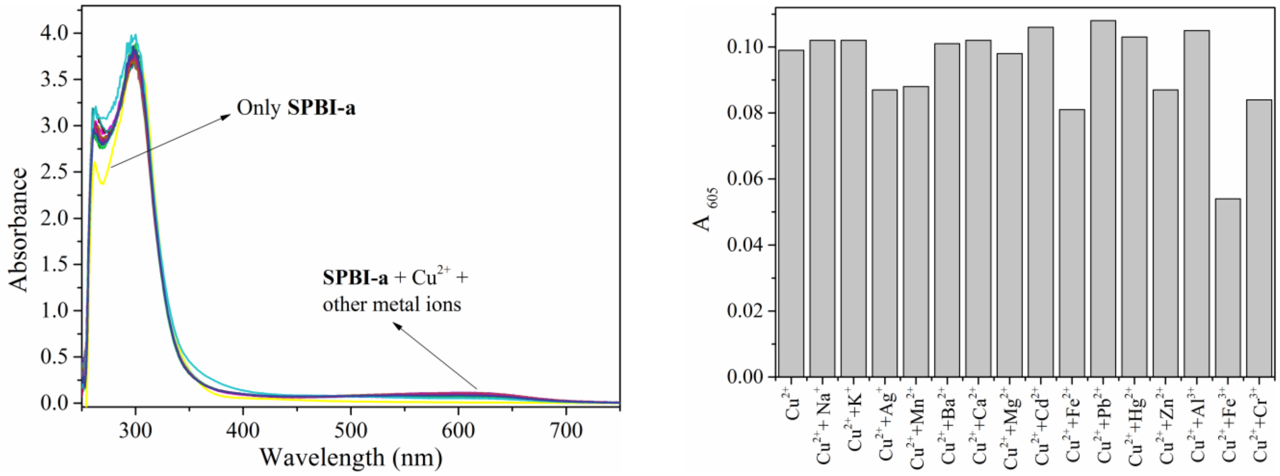

Figure S17. The changes of UV-*vis* absorption spectra (left) and absorbance of the system at 605 nm (right) of probe **SPBI-a** (DMSO/H_2O, V/V = 99/1) after the addition of Cu^{2+} and different metal ions (50 μM). Related to **Figure 4**.

12. UV-*vis* absorption spectra of SPBI-a and absorbance changes at 275, 303 and 605 nm upon the addition of Cu^{2+}

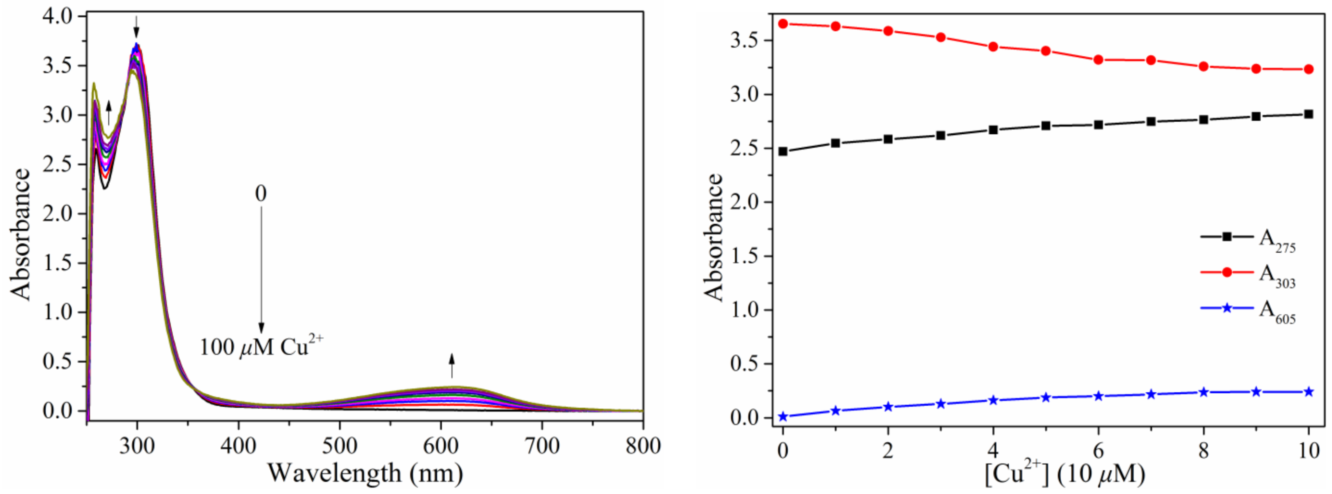

Figure S18. UV-*vis* absorption spectra (left) of **SPBI-a** (1 mg / 15 mL in DMSO/H_2O, *V*/*V*, 99/1) upon the addition of different concentration of Cu^{2+} and the absorbance changes (A_{275}, A_{303} and A_{605}) of sensing system (right) at 275, 303 and 605 nm *vs* concentration of Cu^{2+}. Related to **Figure 4**.

13. The plot of sensor SPBI-a *vs* different concentration of Cu^{2+}

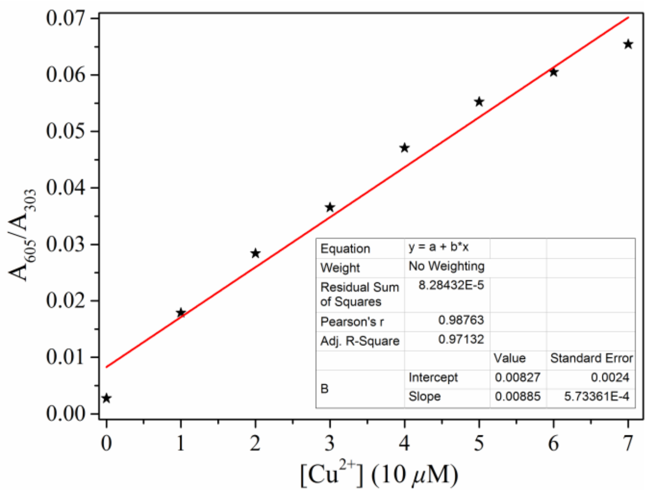

Figure S19. The plot of A_{605}/A_{303} of sensor **SPBI-a** (DMSO/H_2O, *V*/*V* = 99/1) upon the addition of different concentration of Cu^{2+} dissolved in water. Related to **Figure 4**.

14. The selective experiments of SPBI-b towards metal ions

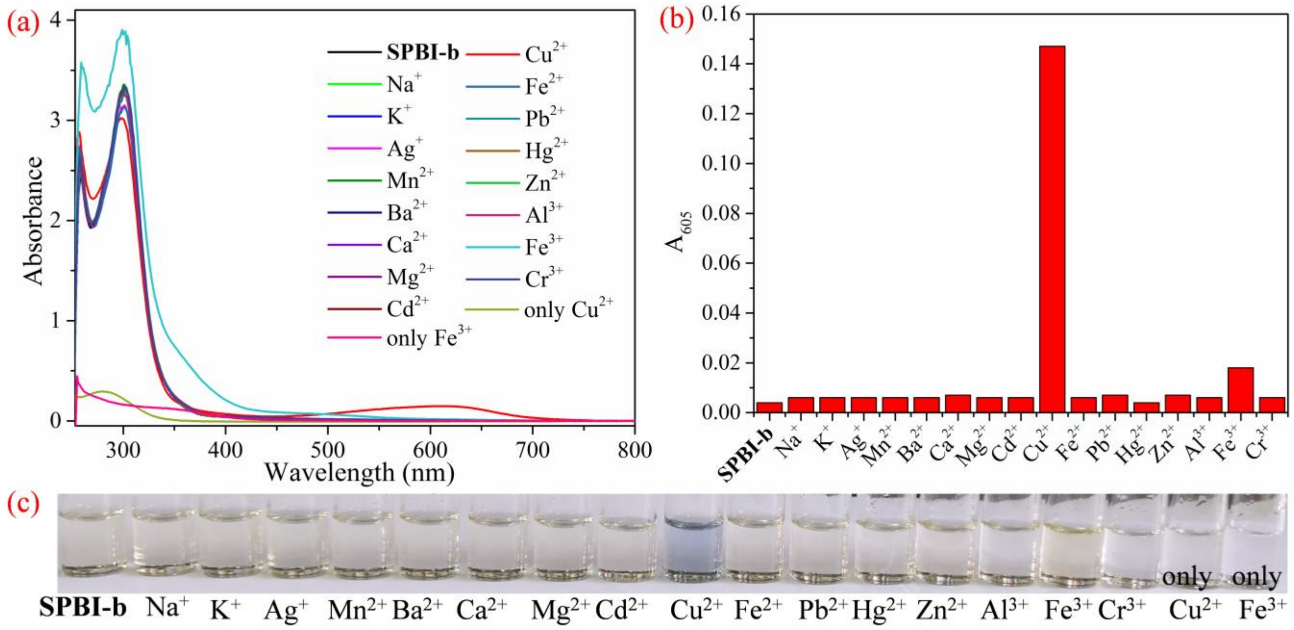

Figure S20. The UV-vis absorption spectra (a), absorbance changes at 605 nm (b) and color changes (c) of **SPBI-b** (1 mg / 15 mL in DMSO/H_2O, *V*/*V*, 99/1) after the addition of various metal ions (50 μM) respectively. Related to **Figure 4**.

15. The competitive experiments of SPBI-b for Cu^{2+} or Fe^{3+}

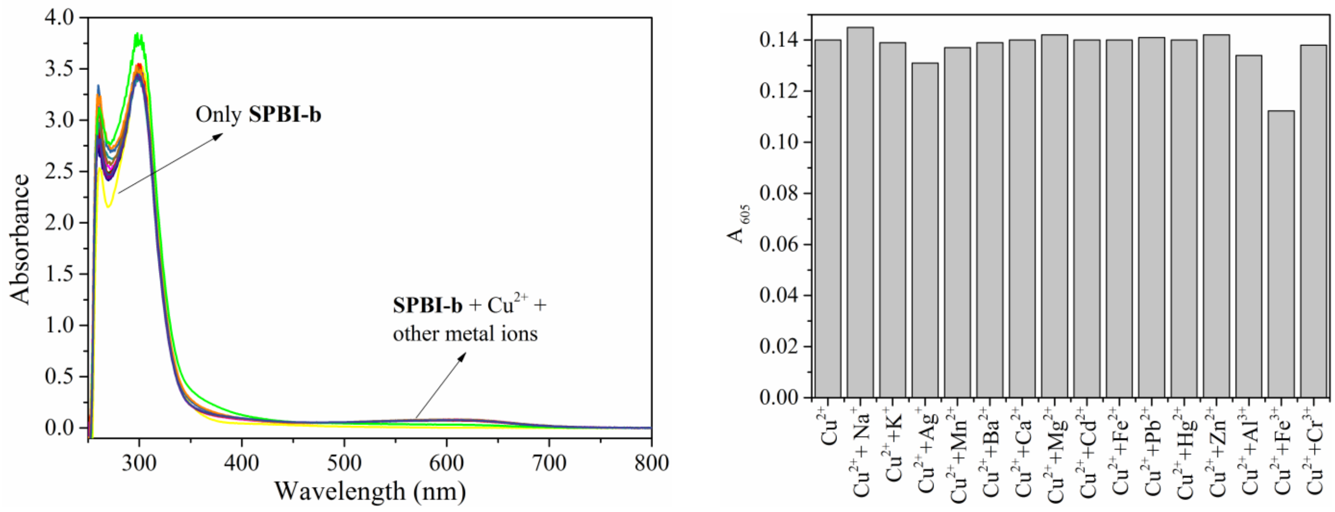

Figure S21. The changes of UV-*vis* absorption spectra (left) and absorbance of system at 605 nm (right) of probe **SPBI-b** (1 mg / 15 mL in DMSO/H_2O, *V*/*V*, 99/1) after the addition of Cu^{2+} (50 μM) and different metal ions (50 μM). Related to **Figure 4**.

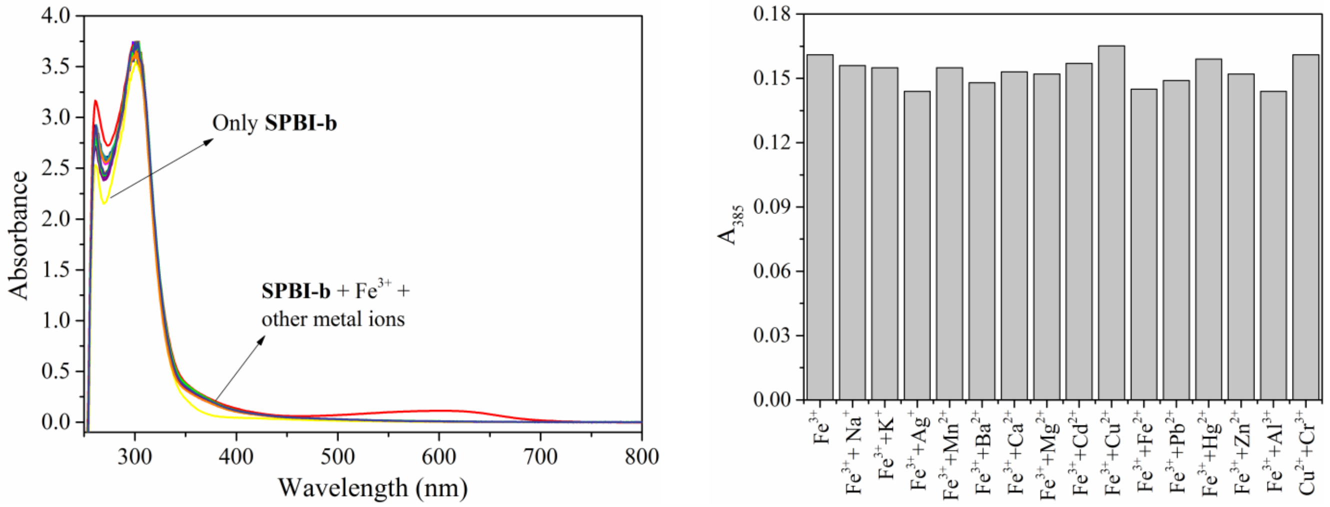

Figure S22. The changes of UV-*vis* absorption spectra (left) and absorbance of system at 385 nm (right) of probe **SPBI-b** (1 mg / 15 mL in DMSO/H_2O, *V*/*V*, 99/1) after the addition of Fe^{3+} (50 μM) and different metal ions. Related to **Figure 4**.

16. UV-*vis* absorption spectra of probe SPBI-b upon the addition of Cu^{2+}

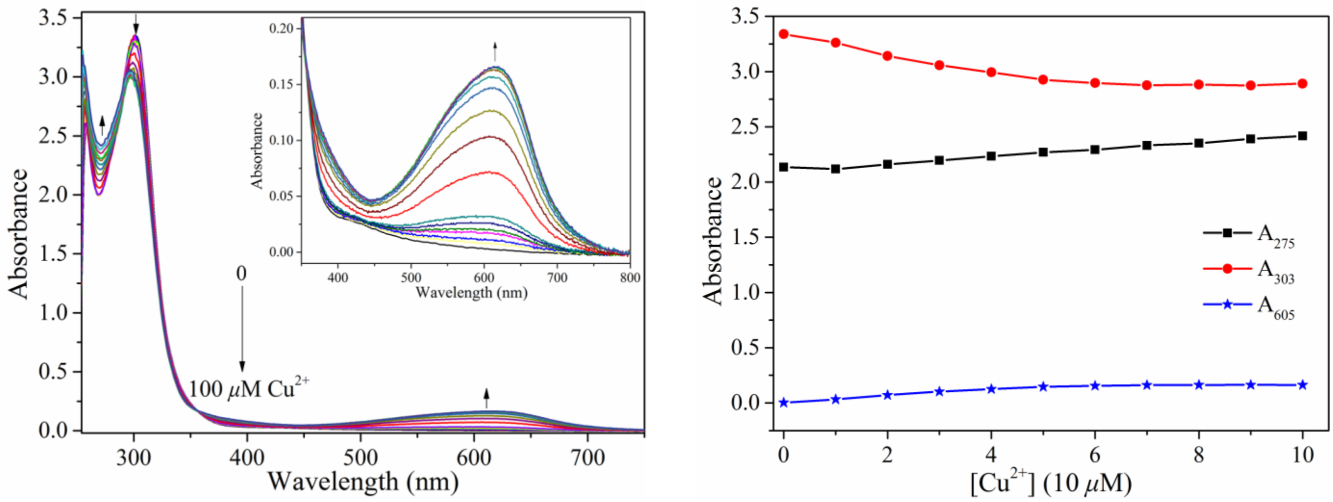

Figure S23. UV-*vis* absorption spectra (left) of probe **SPBI-b** (1 mg / 15 mL in DMSO/H_2O, *V*/*V*, 99/1) upon the addition of different concentration of Cu^{2+} and the absorption changes (A_{275}, A_{303} and A_{605}) of sensing system (right) at 275, 303 and 605 nm *vs* concentration Cu^{2+}. Related to **Figure 4**.

17. The plot of A_{605}/A_{303} of probe SPBI-b *vs* different concentration of Cu^{2+}

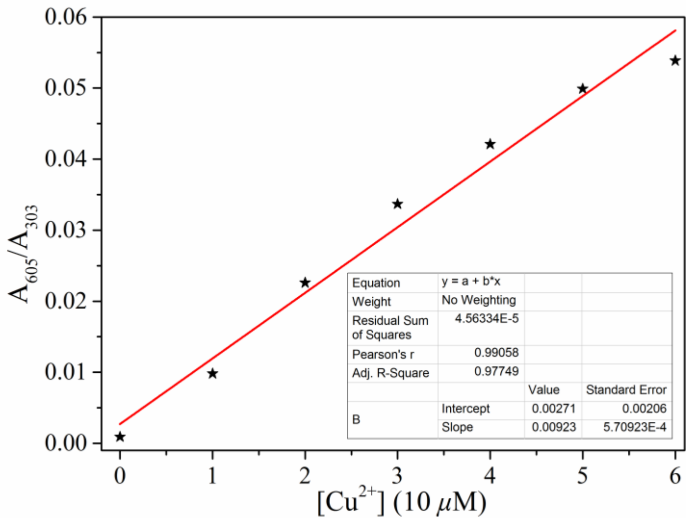

Figure S24. The plot of A_{605}/A_{303} of the probe **SPBI-b** (1 mg / 15 mL in DMSO/H_2O, *V*/*V*, 99/1) upon the addition of different concentration of Cu^{2+} dissolved in water. Related to **Figure 4**.

18. UV-*vis* absorption spectra of probe SPBI-b upon the addition of Fe^{3+}

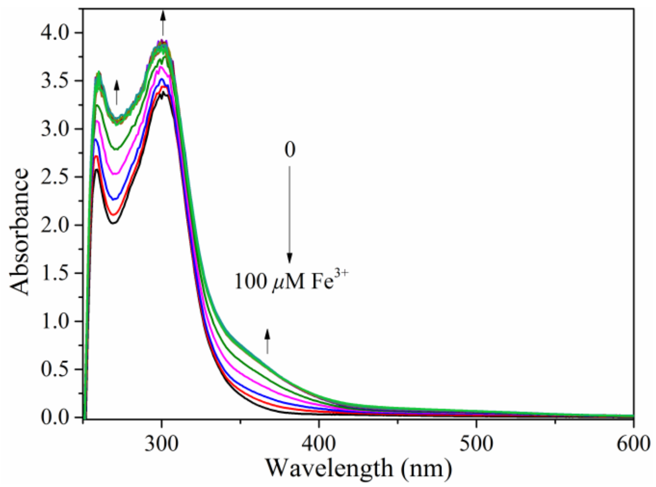

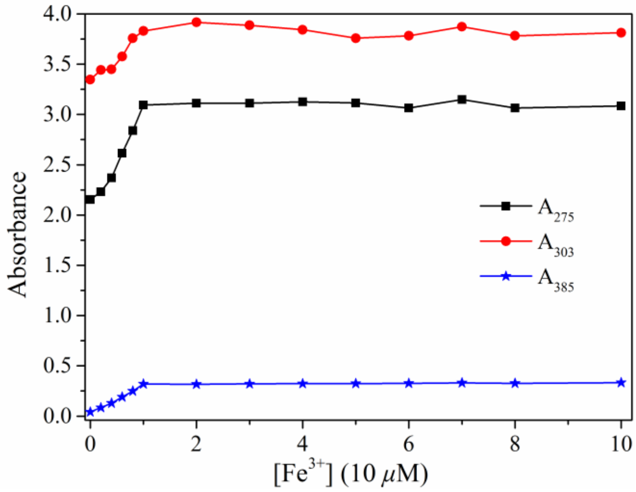

Figure S25. UV-*vis* absorption spectra (left) of probe **SPBI-b** (1 mg / 15 mL in DMSO/H_2O, *V*/*V*, 99/1) upon the addition of different concentration of Fe^{3+} and the absorption changes (A_{275}, A_{303} and A_{385}) of sensing system (right) at 275, 303 and 385 nm *vs* concentration of Fe^{3+}. Related to **Figure 4**.

19. The plot of A_{385} of probe SPBI-b *vs* different concentration of Fe^{3+}

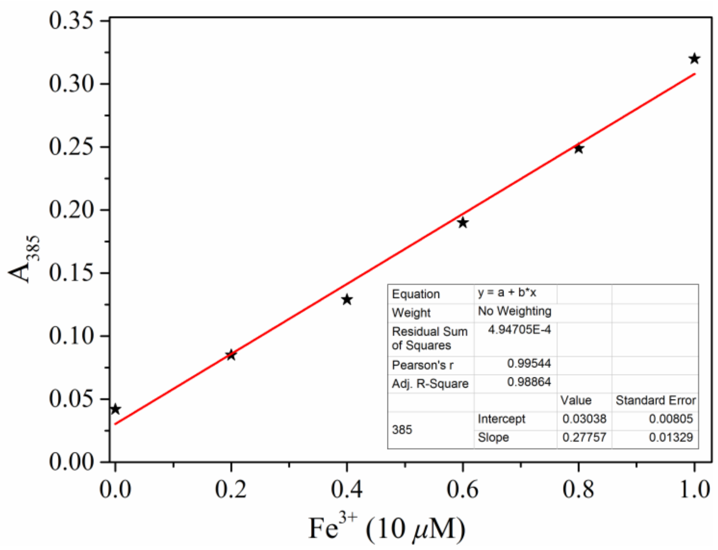

Figure S26. The plot of A_{385} of the probe **SPBI-b** (1 mg / 15 mL in DMSO/H_2O, *V*/*V*, 99/1) upon the addition of different concentration of Fe^{3+} dissolved in water. Related to **Figure 4**.

20. The selective experiments of SPBI-c towards metal ions

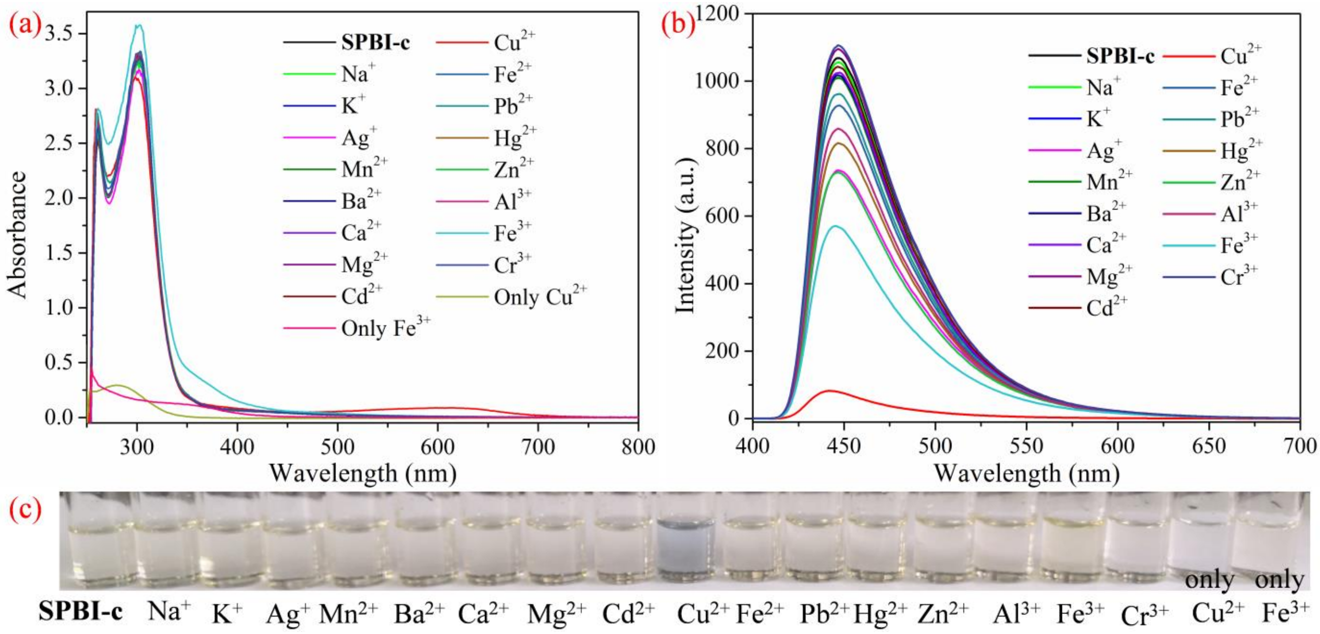

Figure S27. UV-*vis* absorption (a), fluorescence spectra (b) and color changes (c) of **SPBI-c** (1 mg / 15 mL in DMSO/H_2O, *V*/*V*, 99/1) after the addition of different metal ions (λ_{ex} = 331 nm, λ_{em} = 445 nm). Related to **Figure 4**.

21. The competitive experiments of SPBI-c for Cu^{2+} or Fe^{3+}

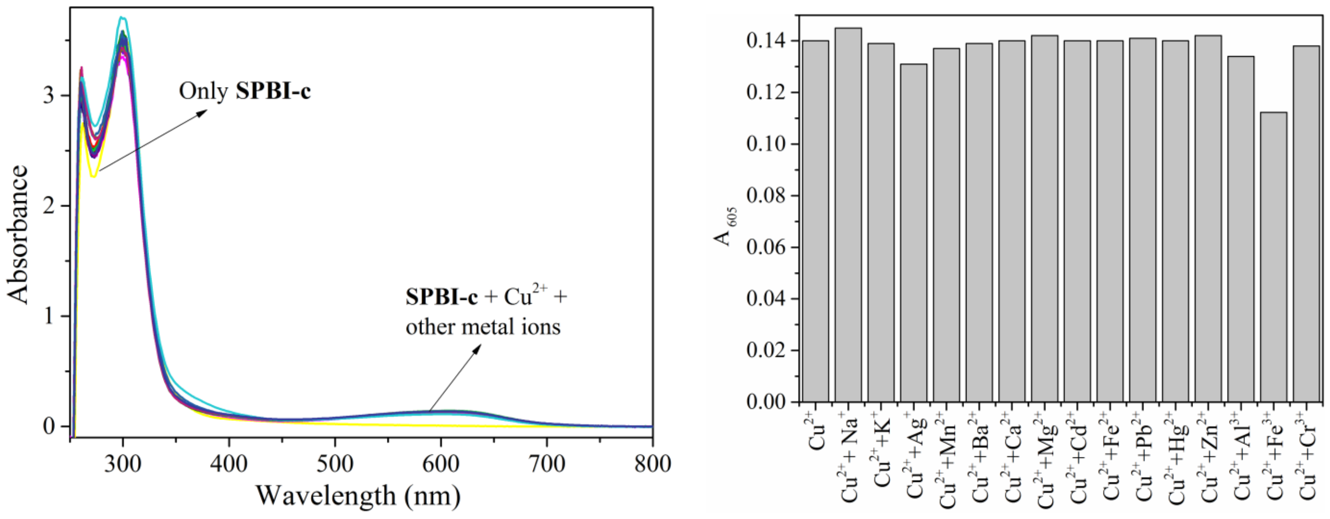

Figure S28. The changes of UV-*vis* absorption spectra (left) and the absorbance of system at 605 nm (right) of probe **SPBI-c** (1 mg / 15 mL in DMSO/H_2O, *V*/*V*, 99/1) after the addition of Cu^{2+} (50 μM) and different metal ions (50 μM). Related to **Figure 4**.

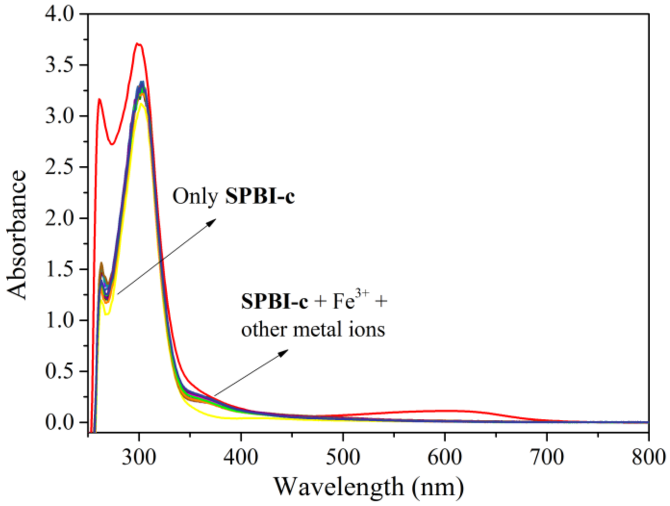

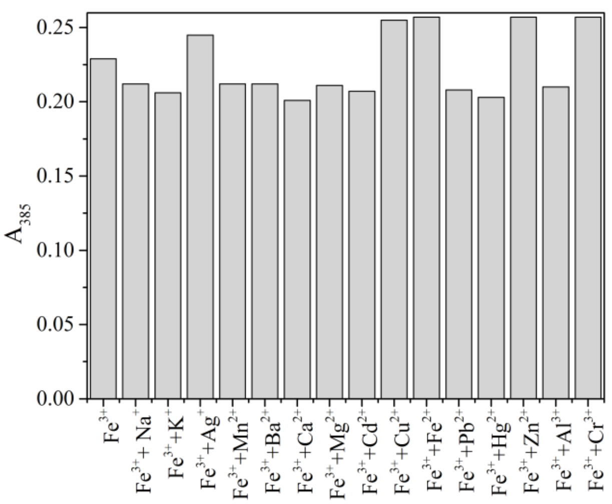

Figure S29. The changes of UV-*vis* absorption spectra (left) and the absorbance of system at 385 nm (right) of probe **SPBI-c** (1 mg / 15 mL in DMSO/H_2O, *V*/*V*, 99/1) after the addition of Fe^{3+} (50 μM) and different metal ions (50 μM). Related to **Figure 4**.

22. The titration experiments of SPBI-c to Cu^{2+} or Fe^{3+}

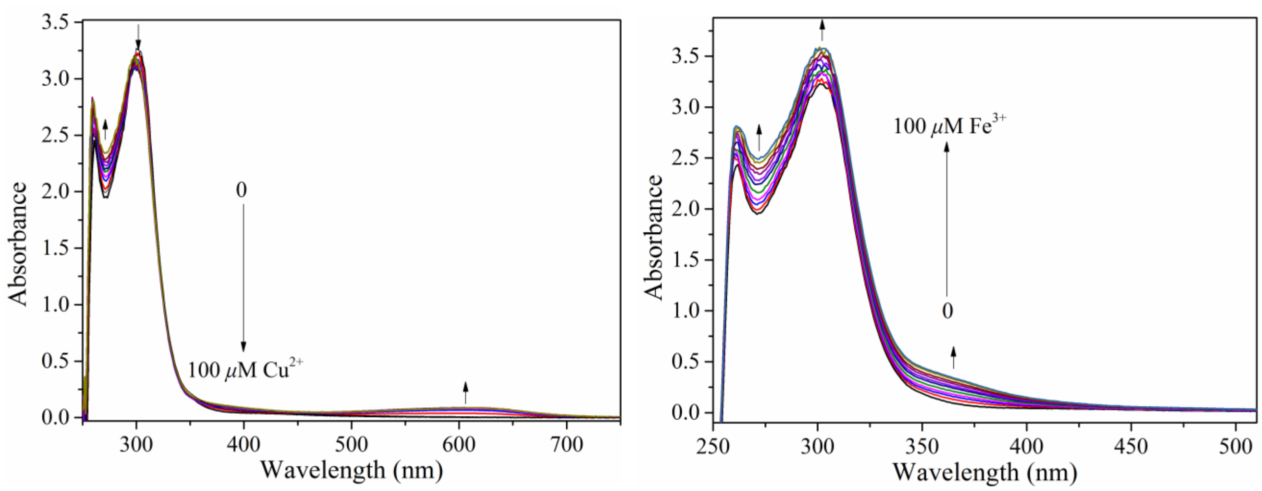

Figure S30. UV-*vis* absorption of probe **SPBI-c** (DMSO/H_2O, V/V = 99/1) upon the addition of different concentration of Cu^{2+} (left) or Fe^{3+} (right). Related to **Figure 4**.

23. The absorbance changes of SPBI-c with the addition of Cu^{2+} or Fe^{3+}

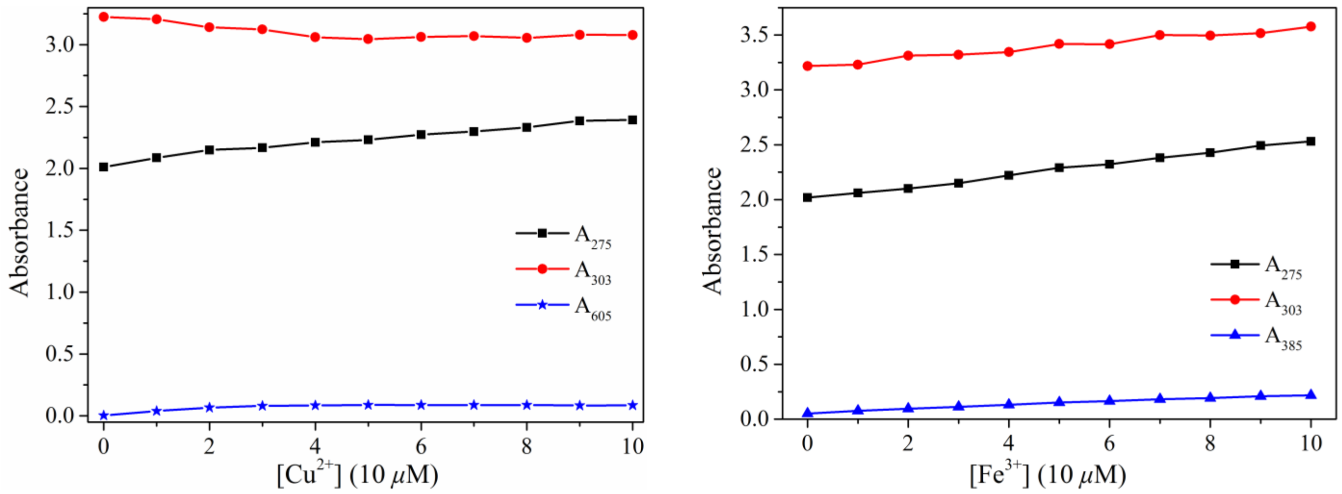

Figure S31. The absorption changes (A_{275}, A_{303} and A_{605}) of sensing system *vs* concentration of Cu^{2+} (left) and A_{275}, A_{303} and A_{385} of sensing system *vs* concentration of Fe^{3+} (right). Related to **Figure 4**.

24. The fluorescence titration experiment of SPBI-c towards Cu^{2+}

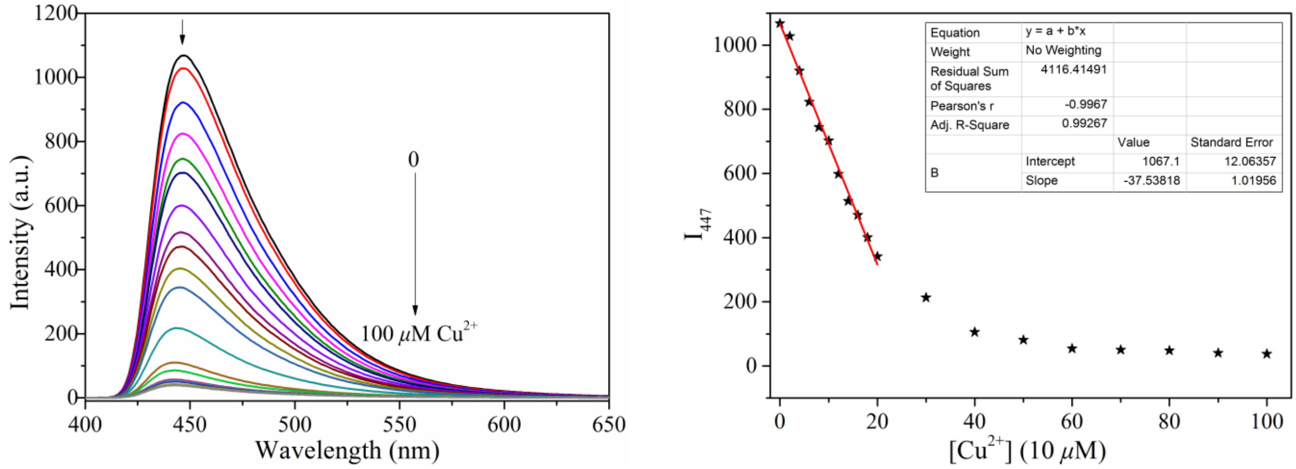

Figure S32. Fluorescent spectra (left) of probe **SPBI-c** (1 mg / 15 mL in DMSO/H_2O, *V*/*V*, 99/1) upon the addition of different concentration of Cu^{2+} and fluorescence changes of sensing system (right) at 447 nm *vs* concentration of Cu^{2+} (λ_{ex} = 331 nm). Related to **Figure 4**.

25. The plot of absorbance of probe SPBI-c *vs* concentration of Cu^{2+} or Fe^{3+}

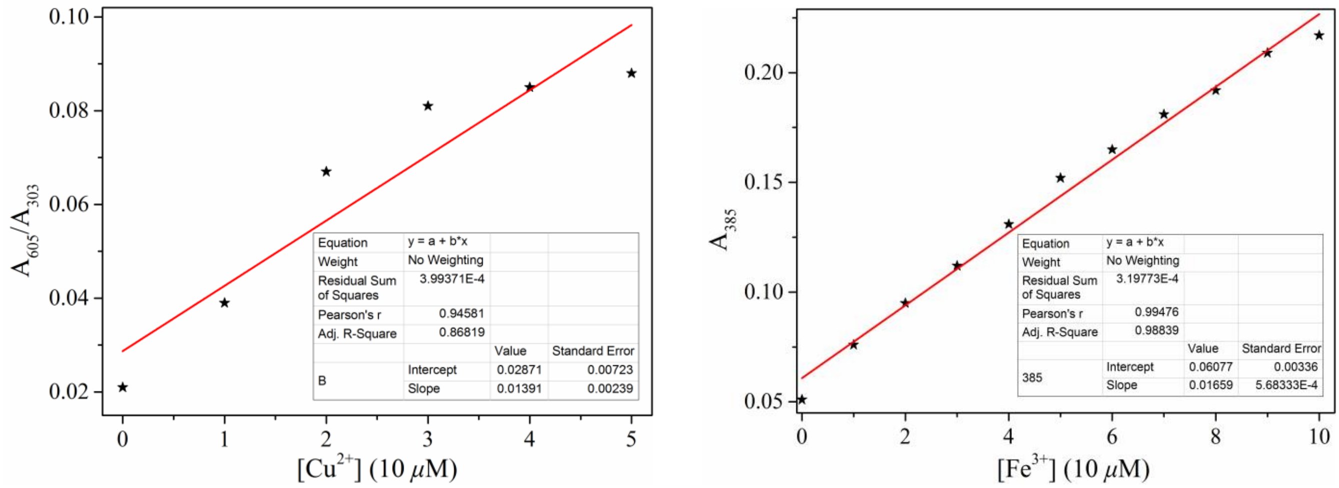

Figure S33. The plot of A_{605}/A_{303} (left) and A_{385} (right) of **SPBI-c** (1 mg / 15 mL in DMSO/H_2O, *V*/*V*, 99/1) *vs* different concentration of Cu^{2+} or Fe^{3+} dissolved in water. Related to **Figure 4**.

26. The selective experiments of SPBI-g towards metal ions

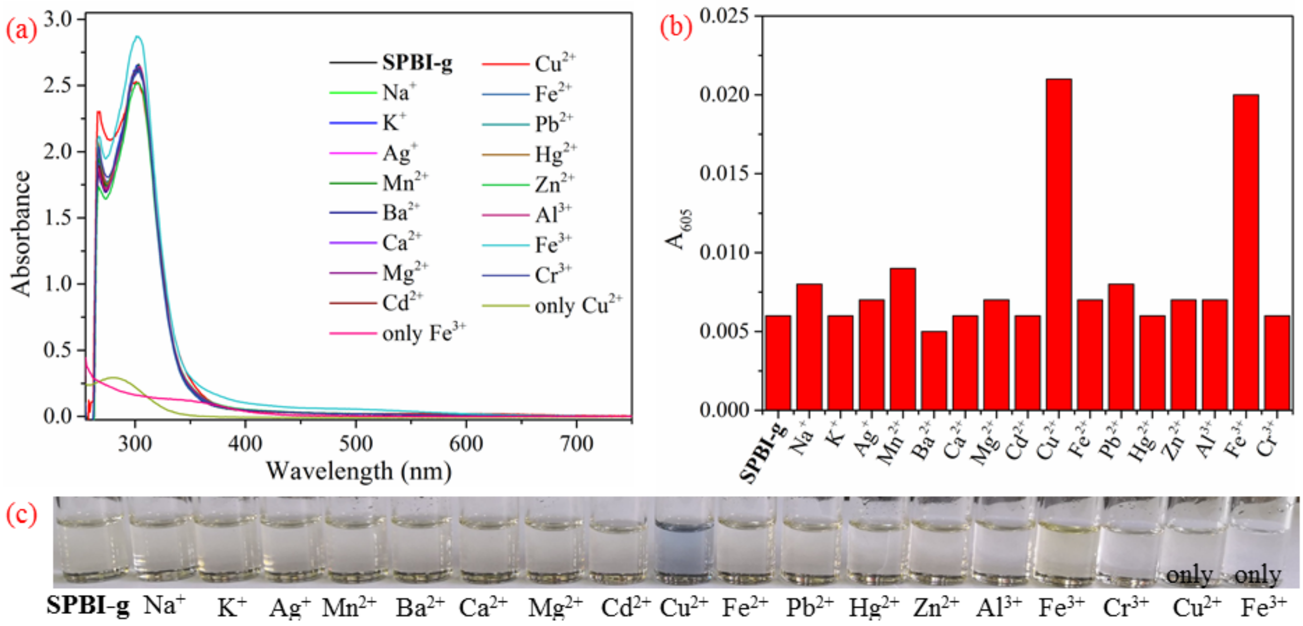

Figure S34. The UV-*vis* absorption spectra (left) and absorbance changes at 605 nm (right) of **SPBI-g** (1 mg / 15 mL in DMF/H_2O, *V*/*V*, 99/1) after the addition of various metal ions (50 μM) respectively. Related to **Figure 4**.

27. The competitive experiments of SPBI-g for Cu^{2+} or Fe^{3+}

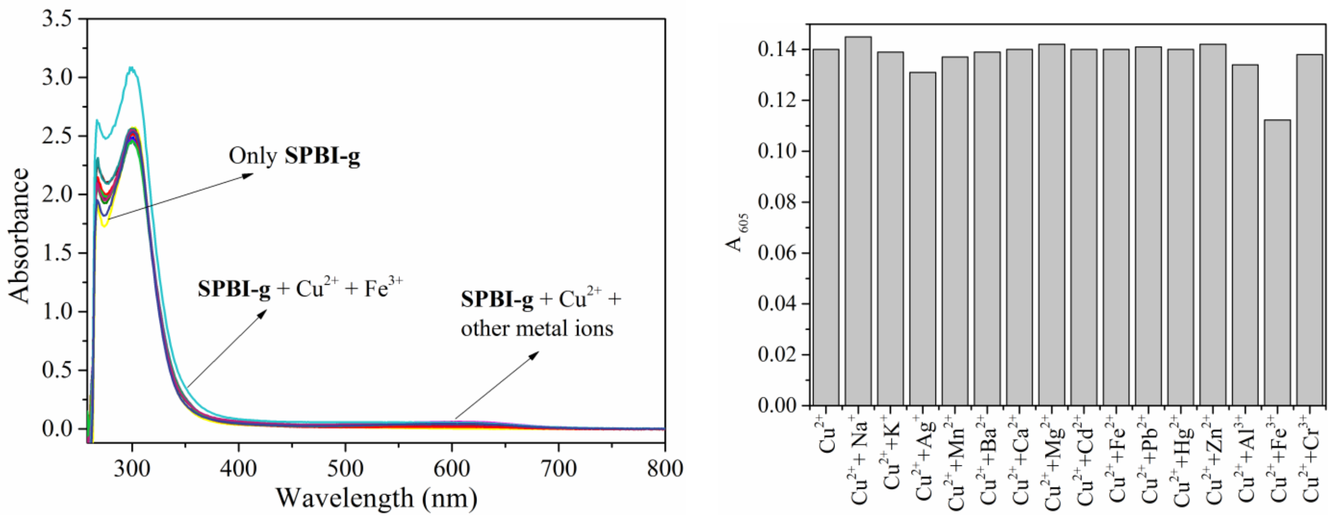

Figure S35. The changes of UV-*vis* absorption spectra (left) and absorbance of the system at 605 nm (right) of probe **SPBI-g** (1 mg / 15 mL in DMF/H_2O, *V*/*V*, 99/1) after the addition of Cu^{2+} (50 μM) and different metal ions. Related to **Figure 4**.

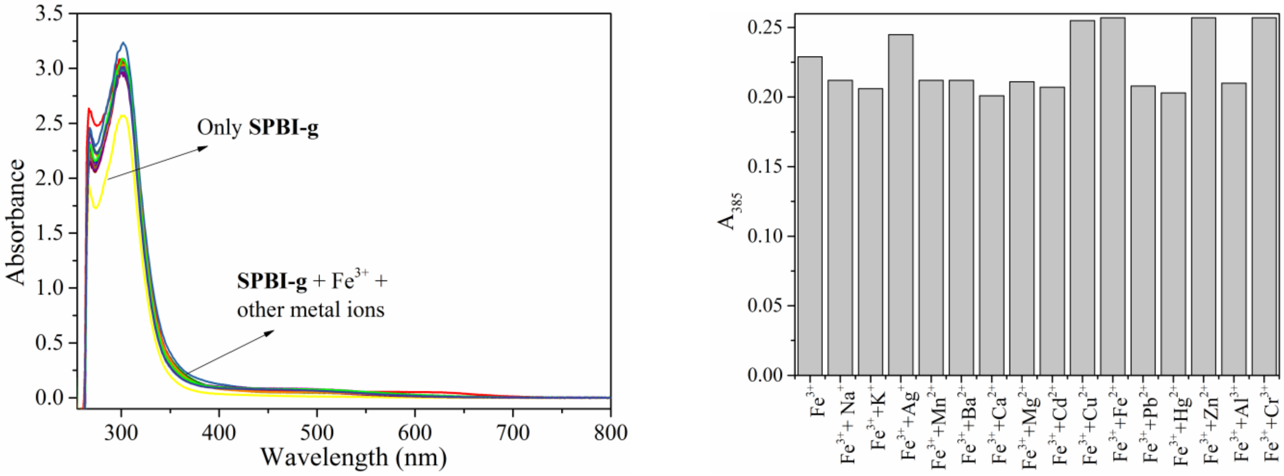

Figure S36. The changes of UV-*vis* absorption spectra (left) and absorbance of the system at 385 nm (right) of probe **SPBI-g** (1 mg / 15 mL in DMF/H_2O, *V*/*V*, 99/1) after the addition of Fe^{3+} (50 μM) and different metal ions. Related to **Figure 4**.

28. UV-*vis* absorption spectra of SPBI-g upon the addition of Cu^{2+} or Fe^{3+}

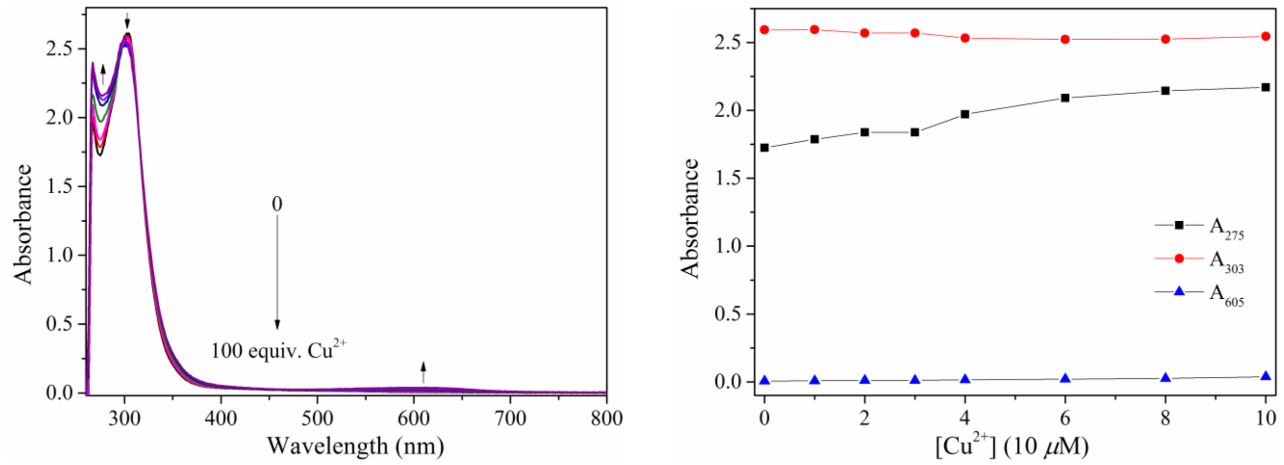

Figure S37. UV-*vis* absorption spectra (left) of **SPBI-g** (1 mg / 15 mL in DMF/H_2O, *V*/*V*, 99/1) upon the addition of different concentration of Cu^{2+} and absorbance changes (A_{275}, A_{303} and A_{385}) of sensing system (right) at 275, 303 and 605 nm *vs* concentration of Cu^{2+}. Related to **Figure 4**.

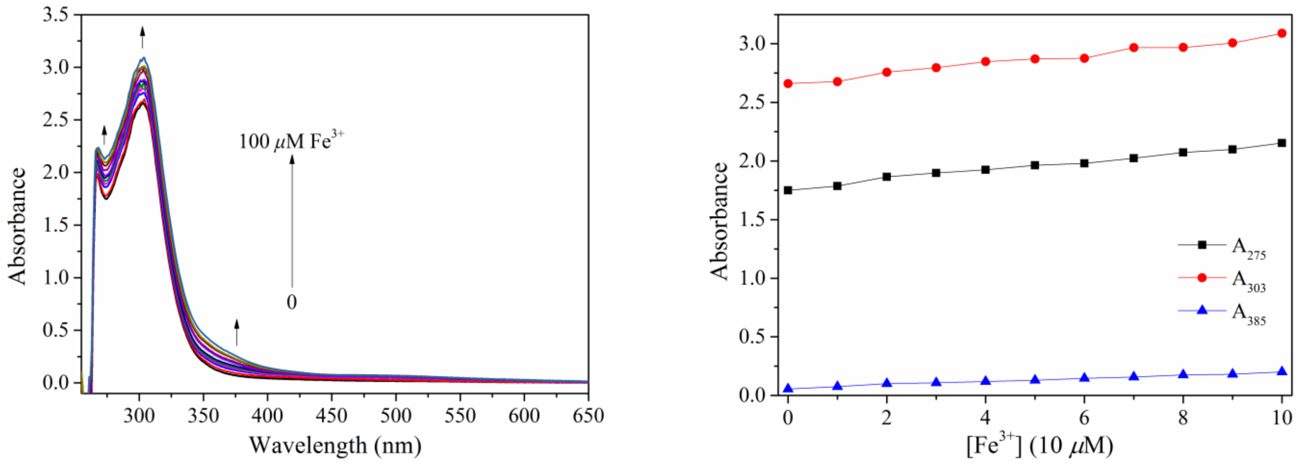

Figure S38. UV-*vis* absorption spectra (left) of **SPBI-g** (1 mg / 15 mL in DMF/H_2O, *V*/*V*, 99/1) upon the addition of different concentration of Fe^{3+} and the absorption changes (A_{275}, A_{303} and A_{385}) of sensing system (right) at 275, 303 and 385 nm *vs* concentration of Fe^{3+}. Related to **Figure 4**.

29. The plot of the absorbance of SPBI-g *vs* concentrations of Cu^{2+} or Fe^{3+}

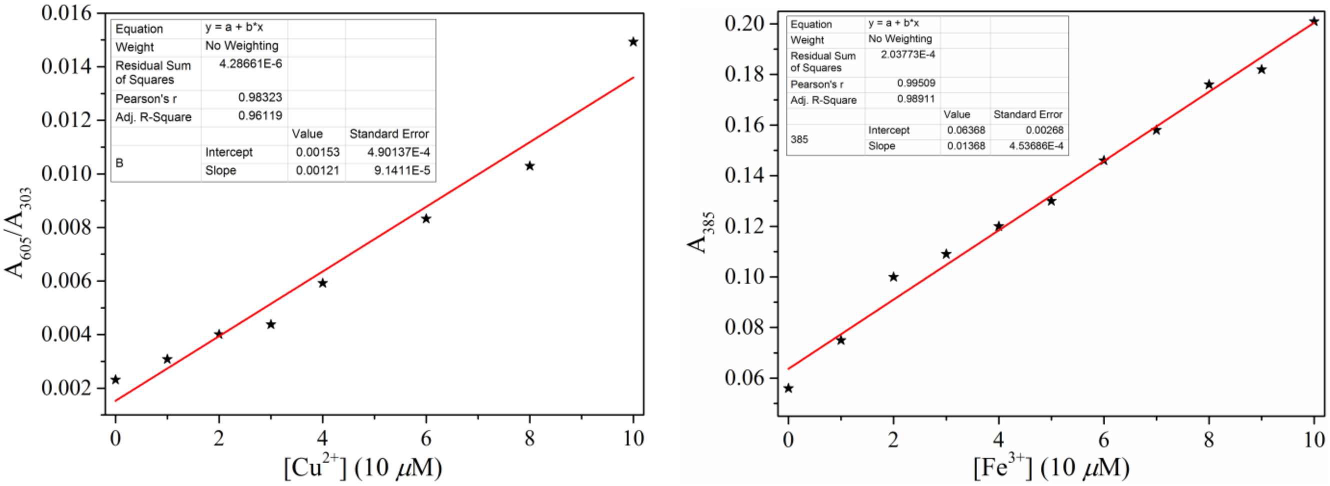

Figure S39. The plot of A_{605}/A_{303} (left) and A_{385} (right) of **SPBI-g** (1 mg / 15 mL in DMF/H_2O, *V*/*V*, 99/1) upon the addition of different concentration of Cu^{2+} or Fe^{3+} dissolved in water. Related to **Figure 4**.

30. The reversible adsorption capacity of SPBI to Cu^{2+}

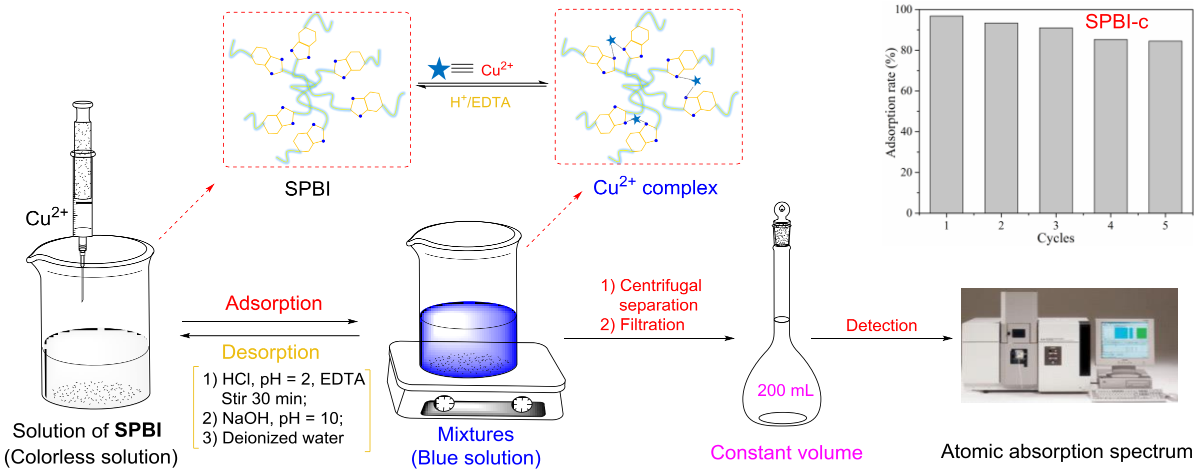

Figure S40. The adsorption and desorption of **SPBIs** to Cu^{2+}. Related to **Figure 5**.

Table S5. The cyclic adsorption result of **SPBI** with different feed ratios to Cu^{2+}. Related to **Figure 5**.

Samples	Cycles	c (Cu^{2+}/ppm) after	c (Cu^{2+}/ppm) before	Adsorption rate (%)	Adsorption capacity (mg/g)
**SPBI-a**	1	6.2312	25.000	75.07	211.72
	2	8.6101	25.000	65.56	184.90
	3	11.105	25.000	55.58	156.75
	4	14.0821	25.000	43.67	123.16
	5	19.0413	25.000	23.83	67.21
**SPBI-c**	1	0.7985	25.000	96.81	273.04
	2	1.6553	25.000	93.38	263.36
	3	2.2465	25.000	91.01	256.68
	4	3.6712	25.000	85.31	240.60
	5	3.8758	25.000	84.49	238.29
**SPBI-g**	1	6.6413	25.000	73.43	207.10
	2	7.7156	25.000	69.14	195.00
	3	11.5972	25.000	53.61	151.20
	4	14.9053	25.000	40.38	113.89
	5	18.0512	25.000	27.79	78.38

31. Structures of the tested nitroaromatic compounds

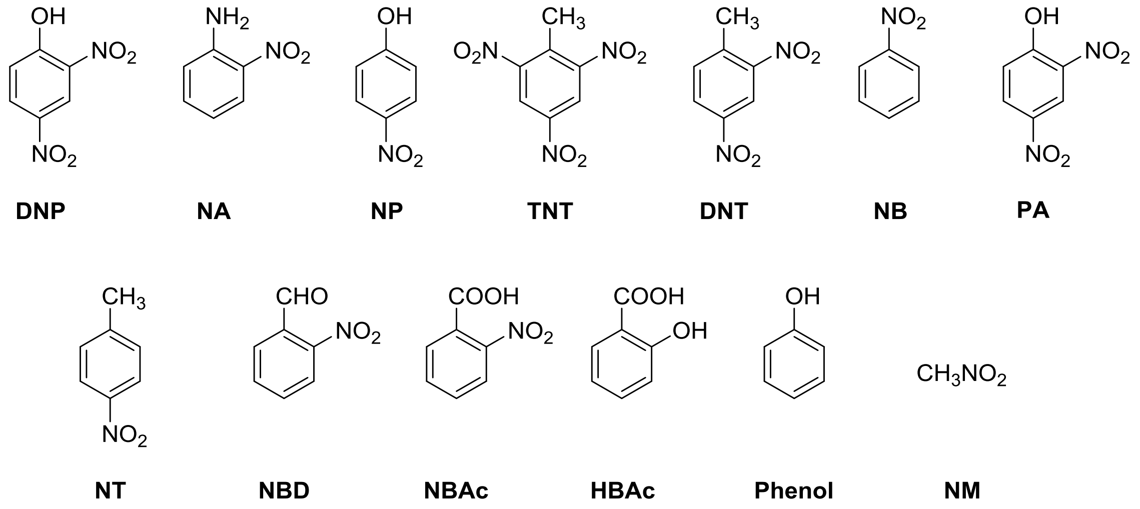

Figure S41. Structures of the tested nitroaromatic compounds (including their reference substances). Related to **Figure 6**.

32. The selective experiments of SPBI-c towards NACs

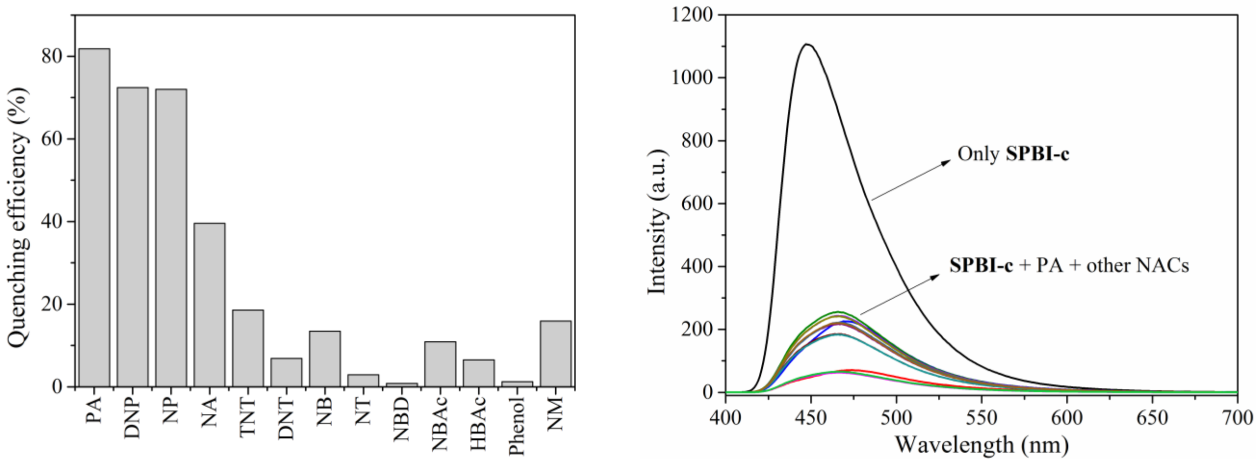

Figure S42. The quenching efficiency (left) and competitive experiments (right) of the system of probe **SPBI-c** (DMSO/H_2O, *V*/*V* = 99/1) after the addition of picric acid (50 μM) and different NACs (λ_{ex} = 331 nm). Related to **Figure 6**.

33. The plots of SPBI-c *vs* concentration of DNP or NP

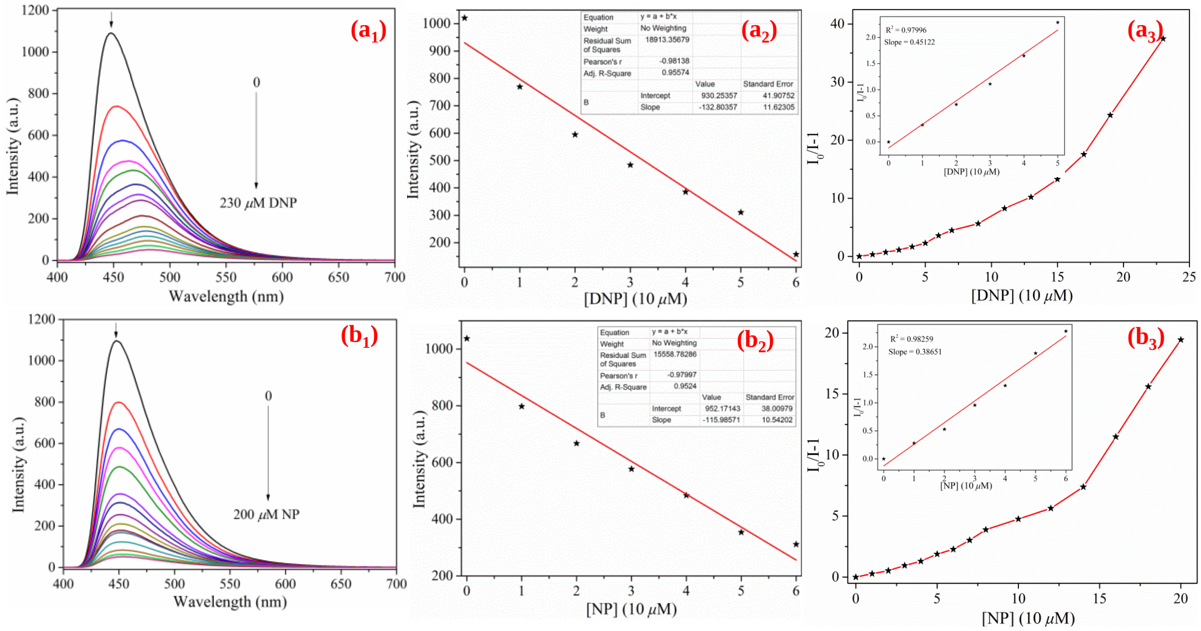

Figure S43. The titration experiments, plot of fluorescence intensity at 447 nm (I_{447}) and Stern-Volmer plot of **SPBI-c** (1 mg / 15 mL in DMSO/H_2O, *V*/*V*, 99/1, λ_{ex} = 331 nm) upon the addition of different concentration of DNP (a) or NP (b). Related to **Figure 6**.

34. The selective and titration experiments of SPBI-g towards NACs

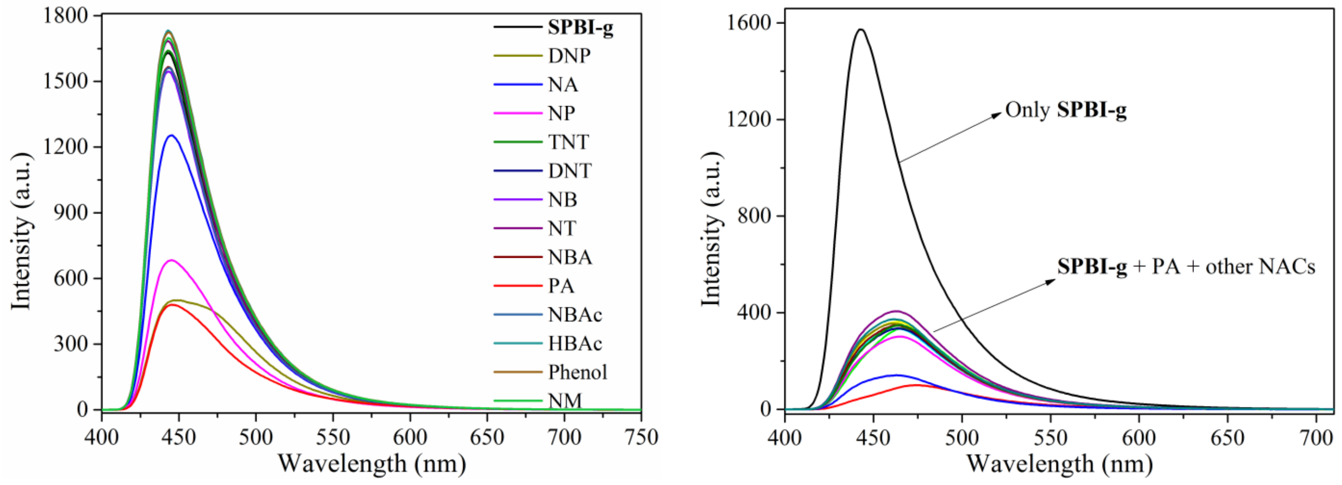

Figure S44. The changes of fluorescence spectra (left) and competitive experiments (right) of the system of probe **SPBI-g** (1 mg/15 mL in DMF/H_2O, *V*/*V*, 99/1, λ_{ex} = 329 nm) after the addition of picric acid (50 μM) and different NACs. Related to **Figure 6**.

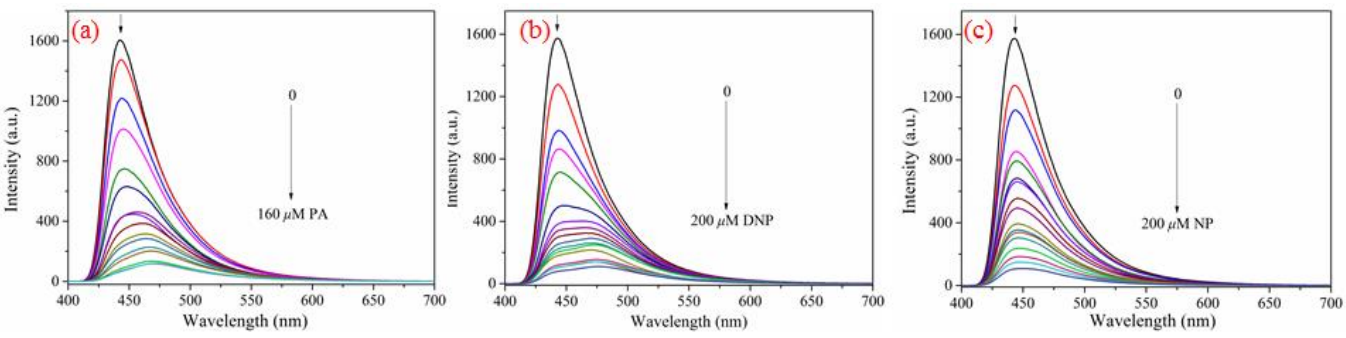

Figure S45. The fluorescent spectra of **SPBI-g** (1 mg / 15 mL in DMF/H_2O, *V*/*V*, 99/1, λ_{ex} = 329 nm) upon the addition of different concentration of PA (a), DNP (b) or NP (c). Related to **Figure 6**.

35. The linear plots and Stern-Volmer plots of SPBI-g *vs* concentration of NACs

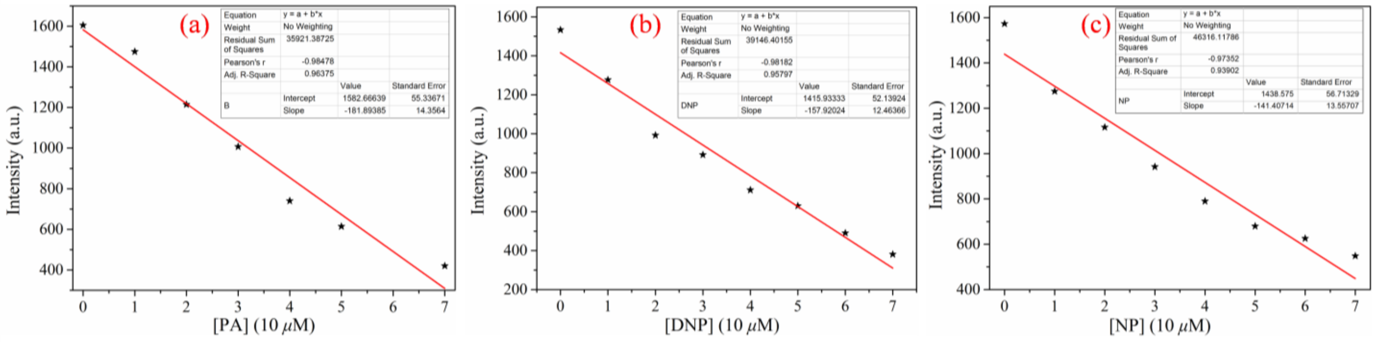

Figure S46. The plot of fluorescence intensity at 447 nm of **SPBI-g** (1 mg/15 mL in DMF/H_2O, *V*/*V*, 99/1) upon the addition of different concentration of PA (a), DNP (b) or NP (c). Related to **Figure 6**.

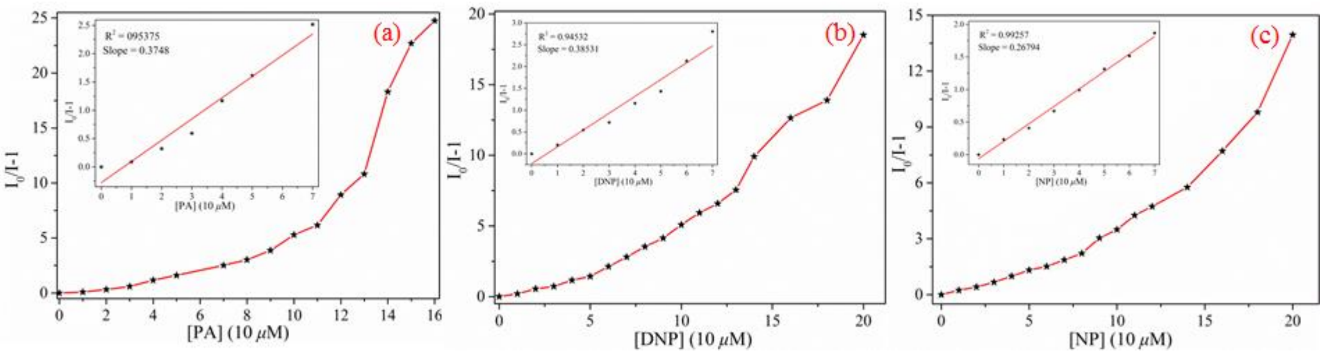

Figure S47. Stern-Volmer plots of **SPBI-g** (1 mg/15 mL in DMF/H_2O, *V*/*V*, 99/1) in response to different concentrations of PA (a), DNP (b) and NP (c); Inset: Stern-Volmer linear plots obtained at lower concentration. Related to **Figure 6**.

36. The changes of FT-IR spectra and morphology before or after the combination with analytes

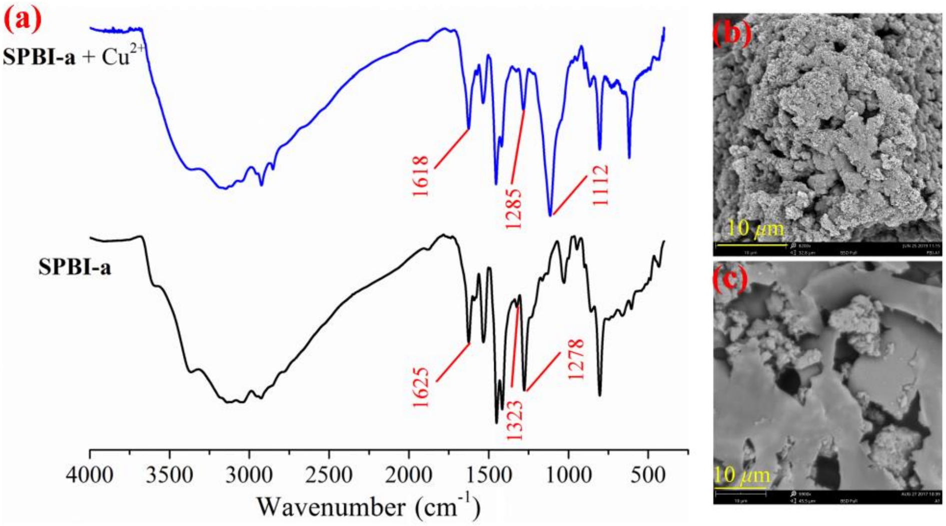

Figure S48. The changes of FT-IR spectra (a) and morphology changes before (b) or after (c) the combination of **SPBI-a** with Cu^{2+} (b: **SPBI-a**; c: **SPBI-a**+Cu^{2+}). Related to **Figure 7** and **Figure 8**.

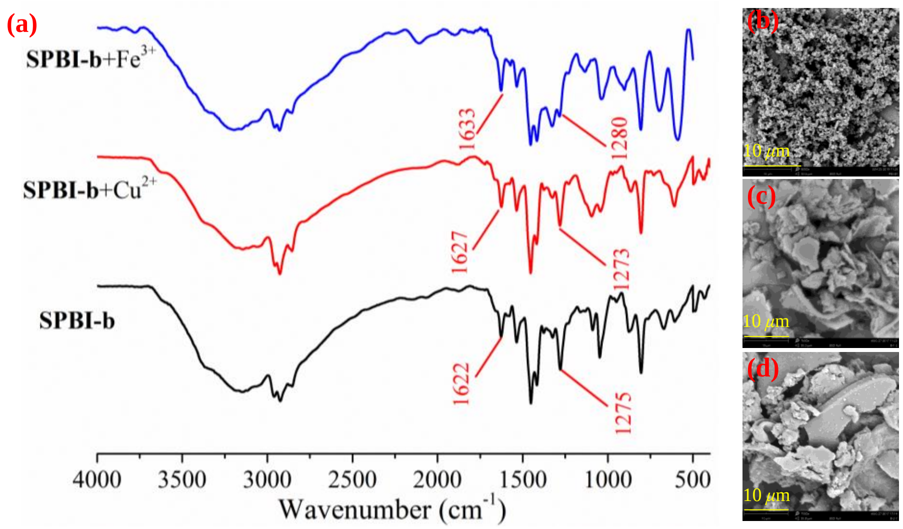

Figure S49. The changes of FT-IR spectra (a) and morphology changes before or after the combination of **SPBI-b** with Cu^{2+} or Fe^{3+} (b: **SPBI-b**; c: **SPBI-b**+Cu^{2+}; d: **SPBI-b**+Fe^{3+}). Related to **Figure 7** and **Figure 8**.

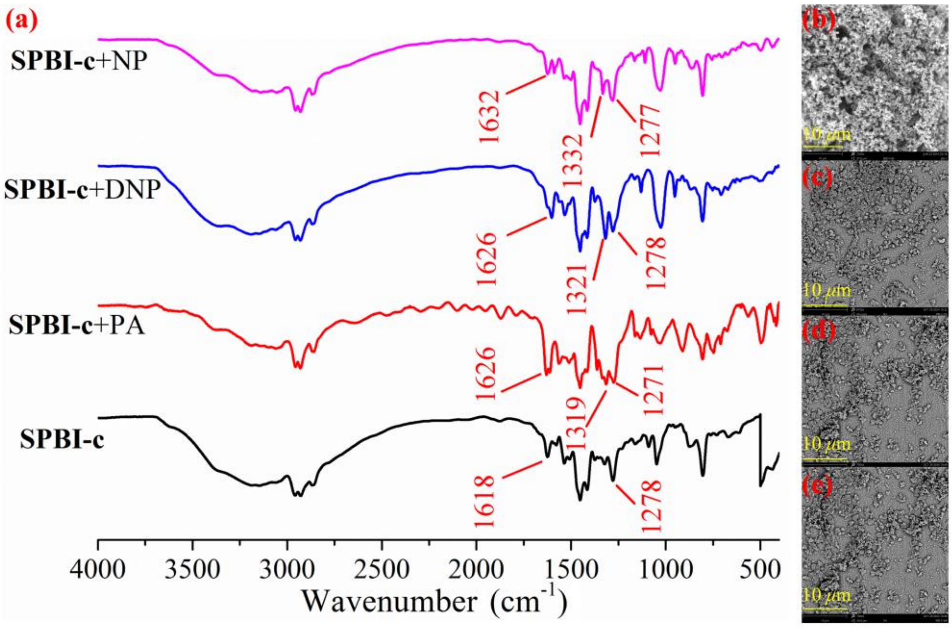

Figure S50. The changes of FT-IR spectra (a) and morphology changes before or after the combination of **PBI-c** with PA, DNP or NP (b: **SPBI-c**; c: **SPBI-c**+PA; d: **SPBI-c**+DNP; e: **SPBI-c**+PA). Related to **Figure 7** and **Figure 8**.

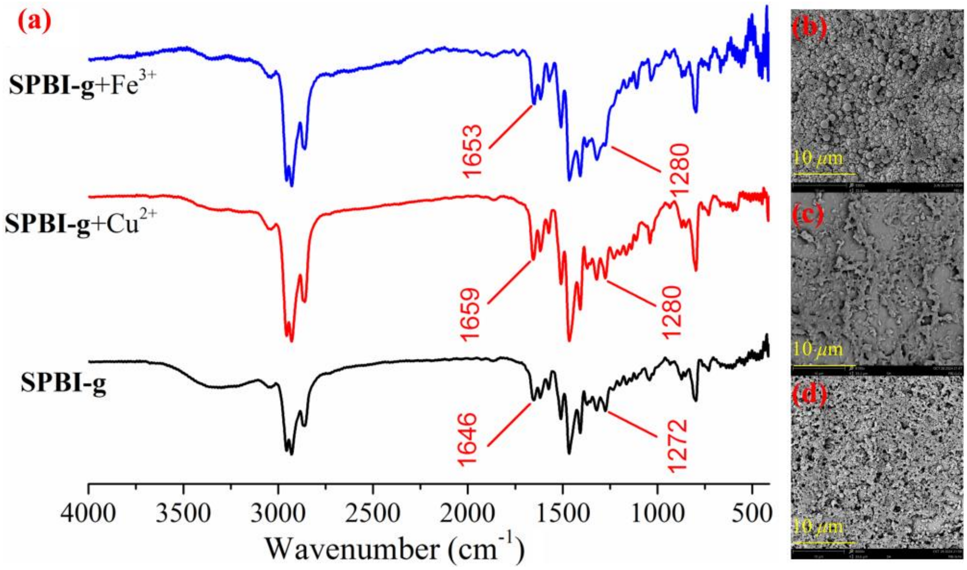

Figure S51. The changes of FT-IR spectra (a) and morphology changes before or after the combination of **SPBI-g** with Cu^{2+} or Fe^{3+} (b: **SPBI-g**; c: **SPBI-g** + Cu^{2+}; d: **SPBI-g** + Fe^{3+}). Related to **Figure 7** and **Figure 8**.

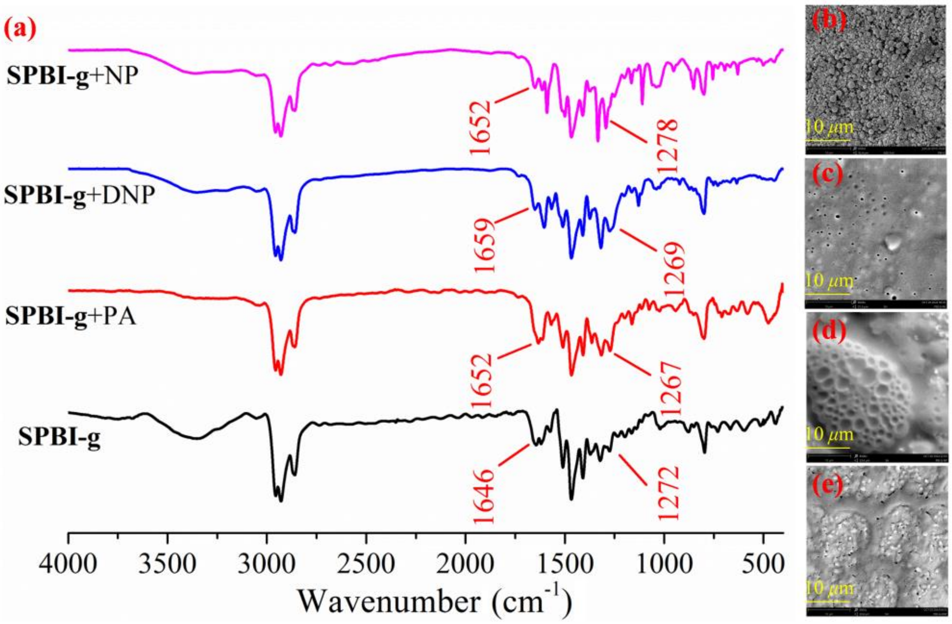

Figure S52. The changes of FT-IR spectra (a) and morphology changes before or after the combination of **SPBI-g** with PA, DNP or NP (b: **SPBI-g**; c: **SPBI-g**+PA; d: **SPBI-g**+DNP; e: **SPBI-g**+PA). Related to **Figure 7** and **Figure 8**.

37. TCSPC plots for SPBI-c and SPBI-g interacted with NACs

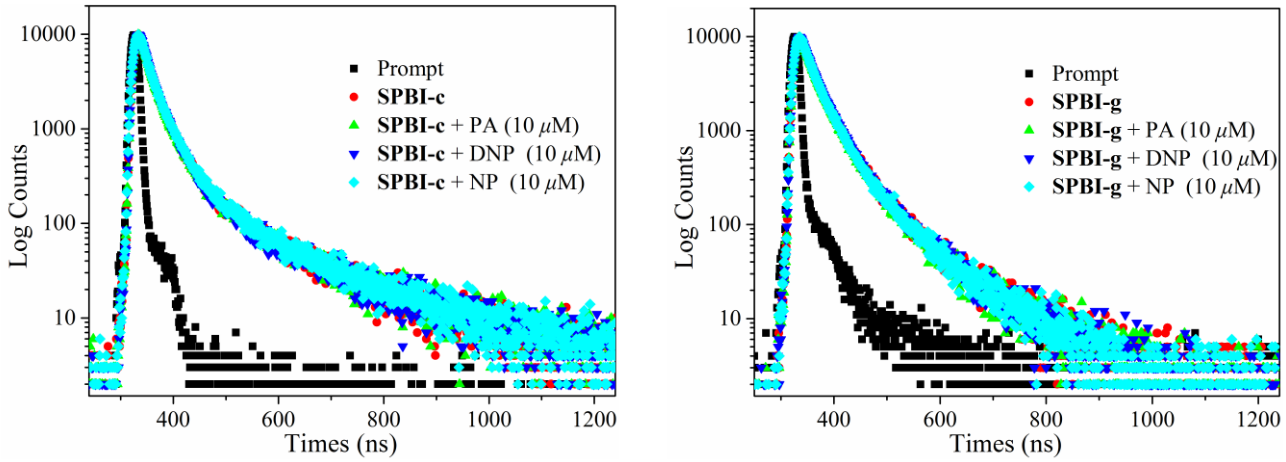

Figure S53. TCSPC plots for **SPBI-c** (left) and **SPBI-g** (right) interacted with PA, NP or DNP. Related to **Figure 6**.

38. The optimized geometries of SPBI-c, its Cu^{2+} complex and PA

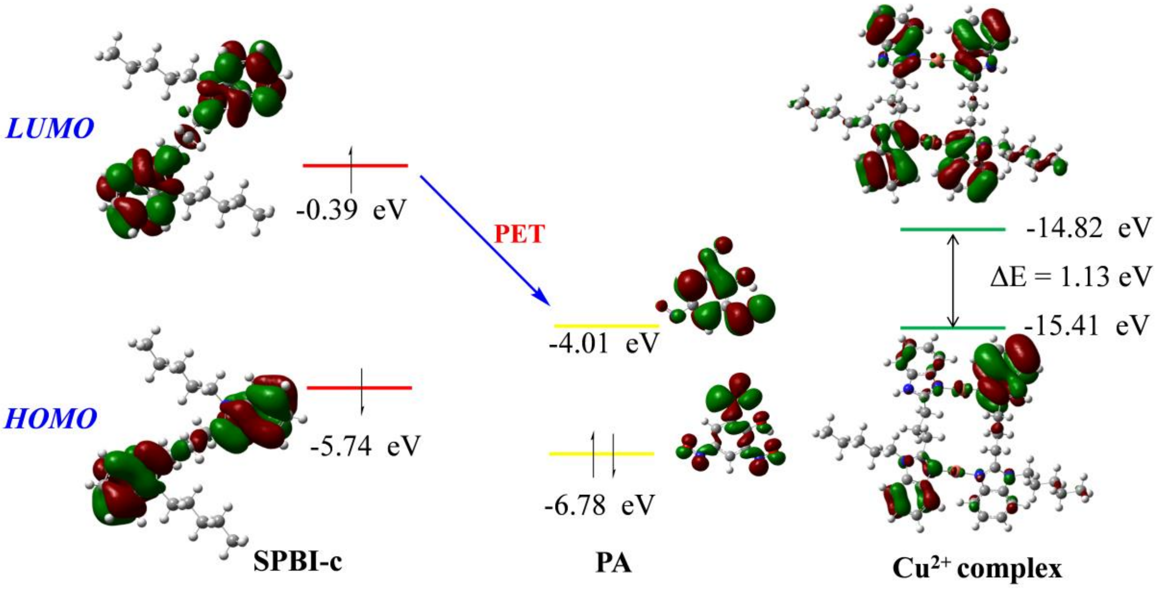

Figure S54. The optimized geometries of **SPBI-c**, its Cu^{2+} complex and PA (B3LYP/6-31G basis set **SPBI-c**, PA and B3LYP/6-31G basis set for C, H, N; LanL2DZ for Cu^{2+} in complex). Related to **Figure 4**, **Figure 6** and **Figure 7**.

39. Comparison of SPBI with Cu^{2+}, Fe^{3+} and PA probes available in the literature

Table S6. The comparison of probe **SPBI** with the reported Cu^{2+} probes in solution. Related to **Figure 4**.

No.	Probe type	Solvent	LOD	Signal types	Ref.
1	Europium based coordination polyelectrolytes	Tris-HCl buffer (pH = 7)	3.4 μM	Turn-off	Wei et al., 2020
2	Calcium coordination polymers	Aqueous solution (pH = 7)	1.0 μM	Turn-off	Wu et al., 2020
3	Quantum dots	-	2.0 μM	Fluorescence ratiometric	Han et al., 2019
4	Naphthopyrandiaminomaleonitrile dyad	CH_3CN	4.03 μM	Turn-off and absorption ratiometric	Zhang et al., 2020
5	Rhodamine B integrated ZIF-8	H_2O	0.191 μM	Fluorescence ratiometric	Du et al., 2020
6	Cholesterol conjugated probe	DMF/Tris HCl (*V*/*V*, 7/3)	5.7 μM	Turn-on	Jia et al., 2020
7	Hydrazono-bridged bis-TPE	THF/H_2O (*V*/*V*, 5/95)	0.485 μM	Turn-off	Jiang et al., 2020
8	Phenanthrene derivatized triazole	CH_3CN	0.54 μM	Turn-off and absorption ratiometric	Landge et al., 2020
9	Rhodamine based	Acetone/H_2O (*V*/*V*, 1/2)	0.69 μM	Turn-off	Hosseinjani-Pirdehi et al., 2020
10	C=N based PAMAM polymer dots	Aqueous solution	7.4 μM	Turn-off	Shia et al., 2020
11	Trinuclear Zinc coordination cluster	EtOH	0.0226 μM	Turn-off	Ke et al., 2020

12	Quinoline-based	DMSO/H_2O (*V*/*V*, 2/8, pH = 7.4)	0.301 μM	Turn-off	Mal et al., 2020
13	Schiff-based	DMSO/H_2O (*V*/*V*, 2/8)	1.09 μM	Fluorescent and absorption ratiometric	Chiou et al., 2020
14	Benzothiadiazole-based	DMF	0.11 μM	Turn-off	Tian et al., 2019
15	Polythiophene-based material	DMSO	0.45 μM	Turn-off	Qian et al., 2019
16	G-quadruplex-based	HEPES buffer	0.3 μM	Turn-off	Ma et al., 2020
17	4,5-quinolimide-based	CH_3CN/H_2O (*V*/*V*, 4/1)	0.44 μM	Turn-off	Zhang et al., 2020
18	Diaminomaleonitrile-appended BODIPY	CH_3CN	0.292 μM	Absorption ratiometric	Li et al., 2020
19	Sponge cellulose fluorescence spherical	Aqueous solution	0.11 μM	Turn-off	Yu et al., 2020
20	Riboflavin functionalized dextrin-sodium alginate based	Aqueous solution	0.445 μM	Turn-off	Sharma et al., 2019
21	Triaminoguanidine-triphenylamine conjugate	MeOH/tris-HCl (*V*/*V*, 6/4)	0.188 μM	Colorimetric	Mathivanan et al., 2020
This work	**SPBI-a**<br>**SPBI-b**<br>**SPBI-c**	DMSO/H_2O (*V*/*V*, 99/1)	0.876 μM<br>0.45 μM<br>0.34 μM	Colorimetric	
	**SPBI-g**	DMF/H_2O (*V*/*V*, 99/1)	3.93 μM	Colorimetric	

Table S7. The comparison of probe **SPBI** with the reported Fe^{3+} probes in solution. Related to **Figure 4**.

No.	Probe type	Solvent	LOD	Signal types	Ref.
1	Cyclosiloxane-linked porous polymers	EtOH	0.13 μM	Turn-off	Sun et al., 2020
2	Polyethylenimine / salicylaldehyde modified cellulose Schiff base	Aqueous solution	0.025 μM	Turn-off and colorimetric	Fan et al., 2020
3	Coordination polymers	Aqueous solution	5.91 μM	Turn-off	Li et al., 2020
4	Phenothiazine-tethered 2-aminopyridine-3-carbonitrile	DMSO	4.9 μM	Turn-off	Roja et al., 2020
5	{[Zn(L)(dcdps)]}n	Aqueous solution	62.1 μM	Turn-off	Ge et al., 2020
6	Ln-MOFs	Aqueous solution	0.945 μM	Turn-off	Li et al., 2020
7	PNIPAM ended with 9,10-dihydroacridine	PBS (pH = 6.86)	1.32 μM	Turn-off	Cui et al., 2020
8	Tetraphenylethylene-based polymer	MeOH	4.5 μM	Turn-off	Zheng et al., 2020
9	Zn(II) coordination polymer	H_2O	1.59 μM	Turn-off	Xu et al., 2019
10	Epoxy-based polymer	THF/H_2O (*V/V*, 3/1)	10 μM	Turn-off	Ghosh et al., 2019
11	Pb(II) coordination polymer	DMF	-	Turn-off	Miao et al., 2019
12	Mn(II) coordination polymers	Aqueous solution	0.291 μM	Turn-off	Zhang et al., 2019
13	Tb^{3+}-doped nickel coordination polymer	Aqueous solution	2.82 μM	Turn-off	Chen et al., 2019
14	Zn(II) coordination polymers	DMSO/H_2O (*V/V*, 1/1)	0.65 μM	Turn-off	Zhu et al., 2020

15	Carbon dots based chloroplast	Aqueous solution	0.3 μM	Turn-off	Ran et al., 2020
16	Polyurethane-carbon dots	Aqueous solution	2.23 μM	Turn-off	Xiao et al., 2020
17	Organic polymer nanofilms-based	Aqueous solution	44 nM	Turn-off	Feng et al., 2020
18	Thiocyanate-based ionic liquid	Aqueous solution	24.8 μM	Absorption changes	Coldur et al., 2020
19	Quinoline appended pillar[5]arene	MeCN/H_2O (V/V, 1/1)	337 μM	Turn-off	Joseph et al., 2020
20	Carbon quantum dots modified ZnO/CdS nanoparticles	H_2O	0.172 μM	Turn-off	Nan et al., 2020
21	Spiropyran derivative	DMF/H_2O (V/V, 9/1)	0.193 μM	Turn-on	Zhang et al., 2020
This work	**SPBI-b**<br>**SPBI-c**	DMSO/H_2O (V/V, 99/1)	0.0149 μM<br>0.286 μM	Absorption changes	
	**SPBI-g**	DMF/H_2O (V/V, 99/1)	0.347 μM	Absorption changes	

Table S8. The comparison of probe **SPBI** with the reported PA probes in solution. Related to **Figure 6**.

No.	Probe type	Solvent	LOD	Signal types	Ref.
1	Chitosan hydrogel	HAc/H_2O (*V/V*, 99/1)	0.27 μM	Turn-off	Xiong et al., 2019
2	Tripeptide for in-field	THF	0.361 μM	Turn-off	Nandi et al., 2019
3	Alkyl chains with fluoranthene based	$CHCl_3$	0.089 μM	Turn-off	Kasthuri et al., 2019
4	Conjugated polymers based on di(naphthalen-2-yl)-1,2-diphenylethene	THF/H_2O (*V/V*, 1/9)	0.181 μM	Turn-off	Zhuang et al., 2020
5	Cu-MOF	MeCN	1.09 μM	Turn-off	Wu et al., 2019
6	Block copolymer	DMF	0.8 μM	Turn-off	Podasca et al., 2019
7	Fluorene-triazine conjugated polymer	$CHCl_3$	0.388 μM	Turn-off	Krishnan et al., 2019
8	Triphenylamine-based AIE materials	$DMSO/H_2O$ (*V/V*, 1/9)	1.9 μM	Turn-off	Wu et al., 2020
9	Cu-MOF	H_2O	2.71 μM	Turn-off	Zhuang et al., 2020
10	Triptycene based polymers	THF	0.46 μM	Turn-off	Ansari et al., 2020
11	CdS QDs/*N*-methyl polypyrrole hybrids	H_2O	0.46 μM	Turn-off	Abbasi et al., 2019
12	Non-ferrocene conjugated quinoxalines	MeCN	1.37 μM	Turn-off	Rajalakshmi and Palanisami 2020
13	Lanthanide MOF	H_2O	0.26 μM	Turn-off	Zhang et al., 2020
14	Polyhydroquinoline nanoaggregates	DMF/H_2O (*V/V*, 5/95)	0.66 μM	Turn-off	Jigyasa et al., 2020
15	Cationic Ni(II)-MOF	H_2O	0.26 μM	Turn-off	Goswami et al., 2019

16	BODIPY-Based	$MeCN/H_2O$ (*V*/*V*, 8/2)	0.44 μM	Fluorometric and colorimetric	Gao et al., 2019
17	Cd(II) based coordination polymer	MeCN	0.3 μM	Turn-off	Ghorai et al., 2019
18	Succinate-bridged Cd(II)-based coordination polymer	MeCN	0.91 μM	Turn-off	Dutta et al., 2020
19	Carbon dots	DMF	0.75 μM	Turn-off	Bora et al., 2019
20	Dy(III)-based MOF	Aqueous solution	0.71 μM	Turn-off	Rajak et al., 2019
21	Sulfur-doped graphene quantum dots	Aqueous solution	0.093 μM	Turn-off	Kadian et al., 2020
This work	**SPBI-c**	$DMSO/H_2O$ (*V*/*V*, 99/1)	0.181 μM	Turn-off	
	**SPBI-g**	DMF/H_2O (*V*/*V*, 99/1)	0.168 μM	Turn-off	

40. The adsorption comparison of SPBI with the reported Cu^{2+} adsorption material

Table S9. The adsorption comparison of **SPBI** with the reported Cu^{2+} adsorption materials. Related to **Figure 5**.

No.	Adsorbents	Temperature (℃)	pH	Regeneration	Adsorption capacity (mg/g)	Ref.
1	Alkaline lignin (AL)-based modified material	25		Yes	56.19	Wang et al., 2020
2	3D porous tubular network-structured chitosan-based beads	25	6.0	-	240.90	Fan et al., 2020
3	Amidoxime-functionalized polyacrylamide-modified chitosan	25	6.0	-	190.7	He et al., 2021
4	Zoledronate functionalized hybrid nanobiomaterial	25	7.0	-	226.33	Fang et al., 2020
5	Alendronate doped HAP nanomaterial	25	7.0	-	226.6	Ma et al., 2020
6	Silica-based embedded with NiO, MgO nanoparticles	25	7.0	-	69.8	Abuhatab et al., 2020
7	p(HEMA-co-TACYC) hydrogels	25	5.0	Yes	17.24	Ozay et al., 2020
8	Polydopamine modified cyclodextrin polymer	25	6.0	Yes	73.64	Chen et al., 2020
9	Ash-based geopolymers	25	7.0	-	40.0	Darmayanti et al., 2019
10	Urea formaldehyde modified alginate	25	7.0	-	104.32	Qu et al., 2020
This work	**SPBI-a**<br>**SPBI-c**<br>**SPBI-g**	25	7.0	Yes	211.71<br>273.04<br>207.10	
